# Hydrostannylation of Red Phosphorus: A Convenient Route to Monophosphines

**DOI:** 10.1002/chem.202202456

**Published:** 2022-10-06

**Authors:** Jose Cammarata, Daniel J. Scott, Robert Wolf

**Affiliations:** ^1^ Institute of Inorganic Chemistry University of Regensburg 93040 Regensburg Germany; ^2^ Department of Chemistry University of Oxford OX1 3TA Oxford UK

## Abstract

The preparation of valuable and industrially relevant organophosphorus compounds currently depends on indirect multistep procedures involving difficult‐to‐handle white phosphorus as a common P atom source. Herein, we report a practical and versatile method for the synthesis of a variety of monophosphorus compounds directly from the bench‐stable allotrope red phosphorus (P_red_). The relatively inert P_red_ was productively functionalised by using the cheap and readily available radical reagent tri‐*n*‐butyltin hydride, and subsequent treatment with electrophiles yields useful P_1_ compounds. Remarkably, these transformations require only modest inert‐atmosphere techniques and use only reagents that are inexpensive and commercially available, making this a convenient and practical methodology accessible in most laboratory settings.

## Graphical abstract

**Valuable, industrially and academically relevant** monophosphorus compounds are accessible directly from red phosphorus by using commonly available reagents. Only modest inert‐atmosphere techniques are needed, making this a convenient and practical methodology for the preparation of P_1_ compounds from elemental, red phosphorus.

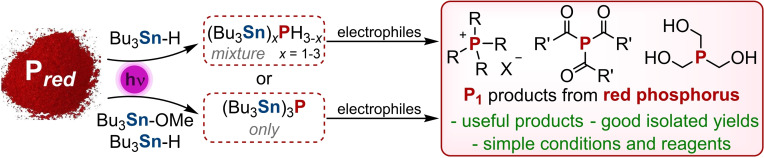

Organophosphorus products are ubiquitous throughout both industry and academia, with a wide range of applications in areas such as medicinal chemistry, materials science, catalysis and coordination chemistry, among many others. In essentially all cases, these compounds are ultimately prepared using elemental phosphorus – particularly white phosphorus (P_4_) – as their common P atom source.^[1]^ However, the transformation of elemental phosphorus into these useful organophosphorus compounds currently relies on undesirable, multistep processes involving extremely hazardous, toxic and/or corrosive reactants and intermediates such as chlorine gas (Cl_2_), phosphorus chlorides (PCl_3_/PCl_5_/POCl_3_) and phosphine gas (PH_3_).[^1^, ^2^] Consequently, much effort has been invested over several decades into the development of alternative, *direct* transformations of elemental phosphorus into useful organophosphorus compounds, which would bypass these hazards while also improving step economy.^[3]^ However, while the past few years have begun to see some progress in this area,^[4]^ direct organofunctionalisation of elemental phosphorus remains in its infancy.^[5]^

The vast majority of studies in this area have focused on the chemistry of white phosphorus (P_4_) due to its molecular nature and industrial relevance.^[6]^ However, while P_4_ may be the principal allotrope used for large‐scale industrial applications, in most other contexts it is a highly undesirable starting material due to its notoriously pyrophoric character and significant toxicity. As a result, its safe handling and productive use typically require specific expertise and equipment (e. g., gloveboxes). These hazards also make P_4_ very difficult to acquire commercially,^[7]^ and collectively these factors render P_4_ an impractical, unattractive or simply impossible precursor for many synthetic laboratories.

An alternative and much more attractive P atom source for academic and other laboratory‐scale chemistry would be the allotrope red phosphorus (P_red_). P_red_ is produced by thermolysis of P_4_ at 200–300 °C,^[1]^ and therefore less attractive from a strictly industrial standpoint. More relevant to most laboratory chemists, however, is that unlike P_4_ (and, notably, other P atom sources relied on by synthetic laboratories such as PCl_3_), P_red_ is both readily and inexpensively available, and bench‐stable.^[8]^ Unfortunately, this bench stability reflects much the lower reactivity of P_red_ in general when compared to P_4_.

P_red_ therefore usually requires much harsher conditions and/or reagents for its activation, which can counteract is attractiveness for laboratory use, and which has contributed to the activation of P_red_ being far less studied than that of P_4_.[^1^, ^8^]

Those few systems that have been reported for the direct transformation of P_red_ typically depend on the use of harsh conditions and extremely strong reagents such as alkali metals. For example, significant contributions in this field have been provided by Trofimov and Gusarova, based on the activation of P_red_ by “superbasic” media (aqueous KOH/DMSO) and the direct phosphorylation of electrophilic alkenes, alkynes and (het)aryl halides to afford organophosphorus compounds such as phosphines, phosphine oxides and phosphinic acids (Scheme 1b, top).[^3b^, ^9^] A different approach, initially reported by Brandsma et al.^[10]^ and later modified by the group of Grützmacher,^[11]^ consists of the reduction of P_red_ with sodium metal and subsequent addition of *t*BuOH, to afford NaPH_2_ as a sodium *tert*‐butanolate aggregate which can also serve as a nucleophilic phosphorus synthon (Scheme 1b, bottom).^[12]^

**Scheme 1:**
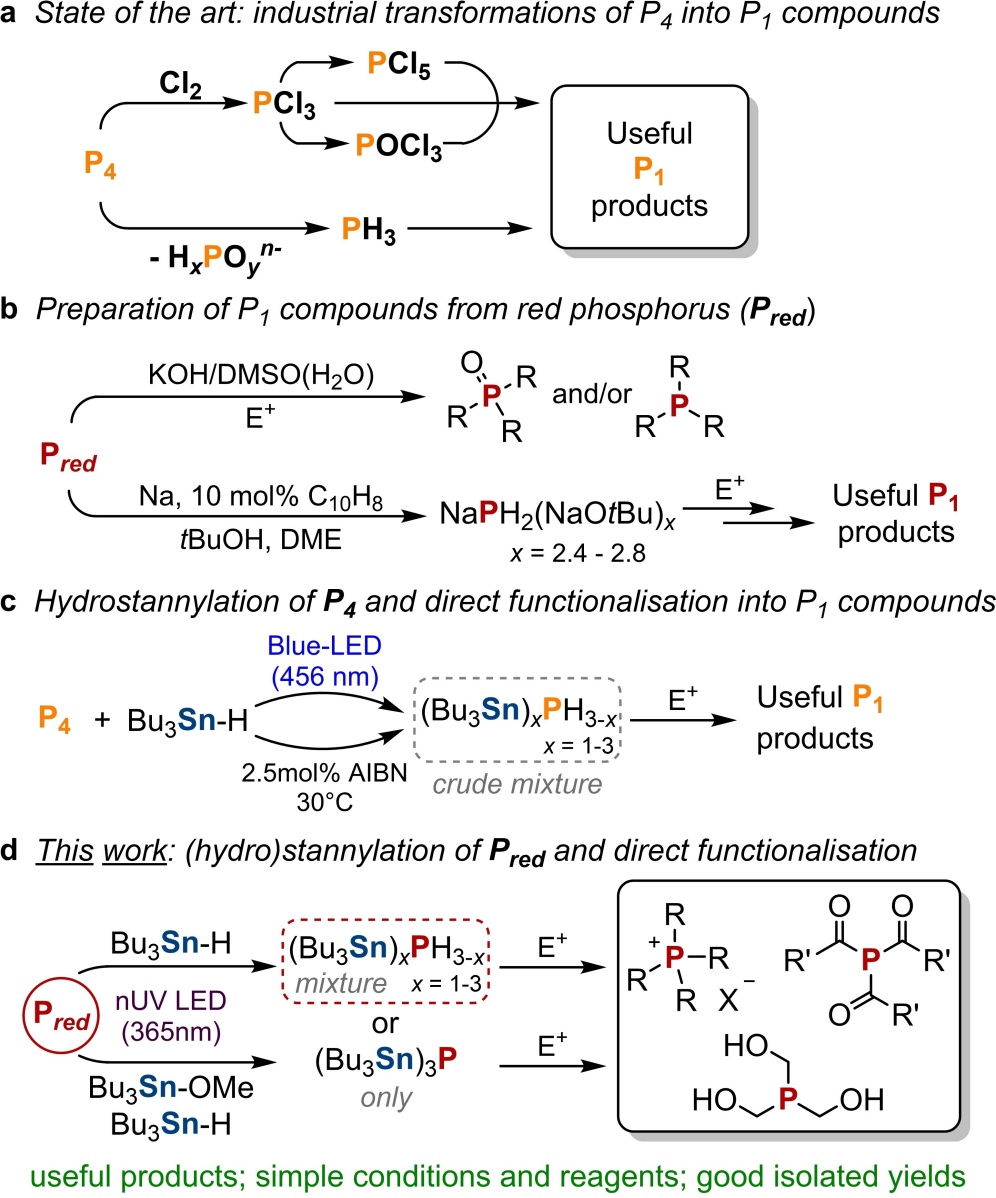
a) Industrial routes for the transformation of P_4_ into P_1_ compounds. b) Previously reported methods for the transformation of P_red_ into P_1_ compounds. c) Hydrostannylation of P_4_ and direct preparation of P_1_ compounds by reaction with electrophiles. d) (Hydro)stannylation of P_red_ and direct transformation into useful P_1_ products by using electrophiles, as described herein. E^+^ represents a generic electrophile.

Recently, we reported a breakthrough in the transformation of *white* phosphorus, by showing that P_4_ can be transformed directly into useful P_1_ products using only simple, readily available and easy‐to‐handle reagents.^[13]^ Specifically, the stoichiometric hydrostannylation of P_4_ was achieved by using the cheap, classical radical reagent tri‐*n*‐butyltin hydride (Bu_3_SnH) in the presence of a suitable radical initiator – either a chemical radical initiator such as azobis(isobutyronitrile) (AIBN) or irradiation with visible light – yielding the stannyl‐substituted monophosphines (Bu_3_Sn)_*x*_PH_3‐*x*_ (*x*=0–3) as a clean mixture. Significantly, each of these products can function as a chemically similar “P^3−^” synthon, allowing the crude (Bu_3_Sn)_*x*_PH_3‐*x*_ mixture to be directly functionalized “as is” with a variety of electrophiles to afford industrially relevant P_1_ compounds in a ‘one‐pot’ fashion (Scheme 1c).^[13]^

Inspired by these results, we speculated that the same hydrostannylation strategy might also be applicable to the transformation of P_red_, and allow the development of a procedure for its direct transformation into P_1_ products that would avoid the very harsh, challenging reagents normally associated with the activation of P_red_. This would also overcome the existing hydrostannylation procedure's current major drawback as a laboratory scale synthetic tool, which is that it requires pyrophoric P_4_ as a substrate. Herein we describe the results of these studies, which have allowed for the simple and efficient preparation of a variety of valuable, industrially and academically relevant monophosphorus compounds directly from P_red_ using only commonly and cheaply available reagents (Scheme 1d). Importantly, this procedure requires only modest inert atmosphere techniques and can be performed without use of a glovebox, making it an unusually convenient and practical approach for the preparation of P_1_ compounds from elemental phosphorus in a typical laboratory setting.

To begin, P_red_ functionalisation was tested using Bu_3_SnH under similar conditions to those used previously for the hydrostannylation of P_4_, as it was anticipated that an equivalent radical chain process should serve to cleave the P−P bonds of P_red_ and thus break its polymeric structure down to the same mixture of P_1_ species.^[13]^ Thus, P_red_ and Bu_3_SnH were combined in PhMe in a 1 : 1.5 molar ratio to reflect the expected reaction stoichiometry, and irradiated with blue LED light (455 nm; chosen for consistency with our previous report) for three days.^[14]^ Very gratifyingly, the formation of the anticipated hydrostannylated monophosphines (Bu_3_Sn)_*x*_PH_3−*x*_ (*x*=0–3) was observed by ^31^P{^1^H} NMR spectroscopy, clearly showing the viability of the desired transformation. Under these reaction conditions the conversion to (Bu_3_Sn)_*x*_PH_3−*x*_ was relatively limited (<20 %) and the corresponding ^1^H NMR spectrum revealed that only a fraction of the Bu_3_SnH was consumed, which is consistent with the more insoluble and inert nature of P_red_ in comparison to P_4_. Nevertheless, using this reaction as a starting point, further investigations revealed that the use of near UV LED irradiation (365 nm), more concentrated reaction mixtures, slightly longer reaction times, and an excess of very cheap P_red_ each led to improved reaction outcomes (see Tables S1 and S2 in the Supporting Information). Remarkably, following optimisation of the reaction conditions (365 nm LEDs, 1 equiv. Bu_3_SnH, 6.7 equiv. P_red_, 1.2 M PhMe, 4 d; Scheme 2a) the desired mixture of PH_3_ (**1**), Bu_3_SnPH_2_ (**2**), (Bu_3_Sn)_2_PH (**3**) and (Bu_3_Sn)_3_P (**4**) could be obtained cleanly and near‐quantitatively (for full details, see Section 1.1 in the Supporting Information). Formally, the optimized reaction proceeds with relatively poor P atom economy, due to the use of an excess of P_red_. While in an industrial context this could be problematic, in a laboratory setting it is mitigated by the extremely low cost of P_red_, even in comparison with Bu_3_SnH; this makes the latter the more sensible limiting reagent.

**Scheme 2:**
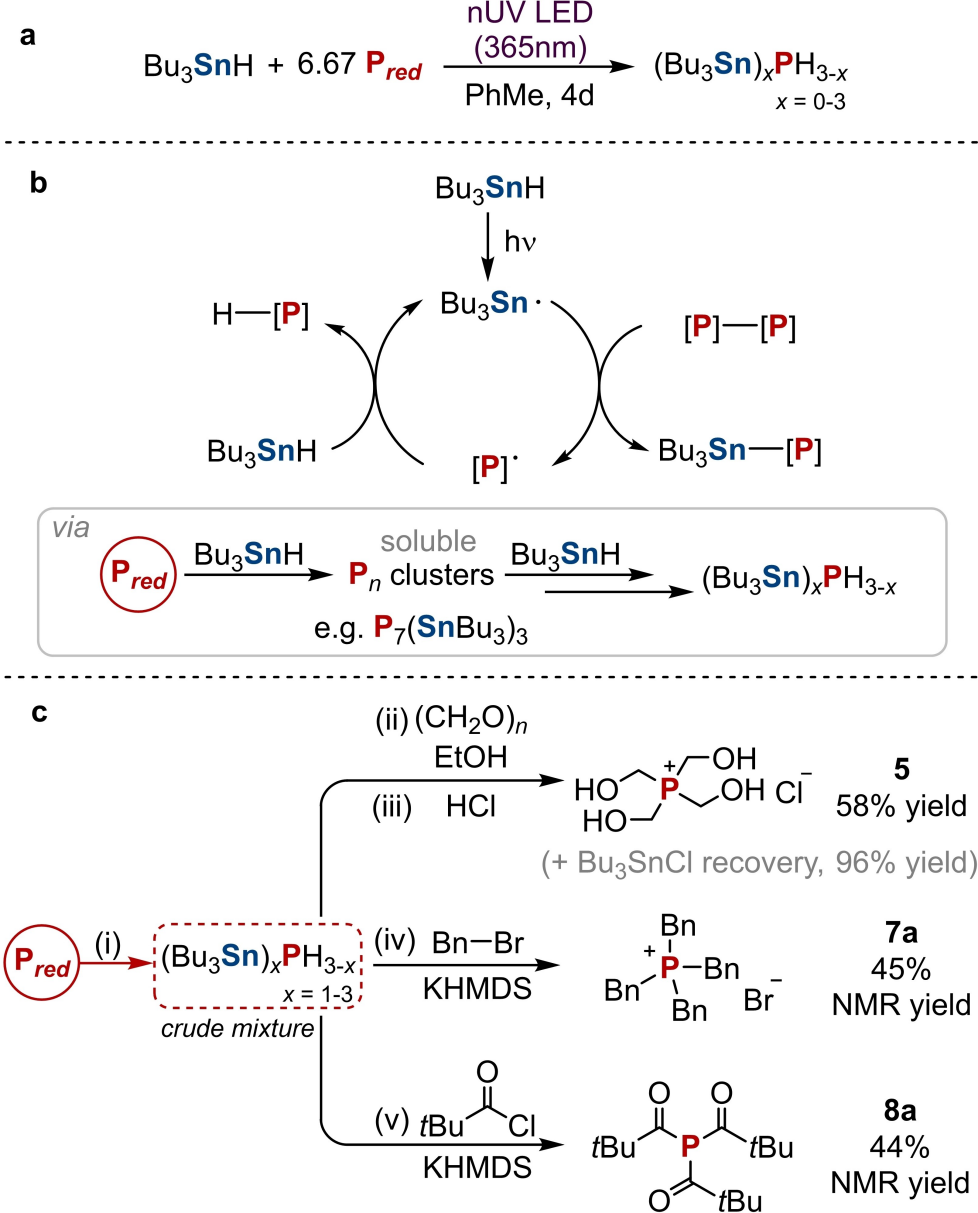
a) Hydrostannylation of P_red_ with Bu_3_SnH promoted by near‐UV irradiation. b) Proposed radical chain mechanism for P_red_ hydrostannylation, proceeding by excision of P_*n*_ clusters such as P_7_(SnBu_3_)_3_. c) Synthesis of P_1_ products directly from P_red_ by hydrostannylation to (Bu_3_Sn)_*x*_PH_3−*x*_ (*x*=1–3). (i) Bu_3_SnH (1 equiv.), P_red_ (6.67 equiv.), PhMe, 365 nm LEDs, RT, 4 days; (ii) EtOH, 8.33 equiv. paraformaldehyde, RT, 16 h; (iii) 6.67 equiv. HCl (4.0 M in 1,4‐dioxane), RT, 2 h; (iv) 6.67 equiv. BnBr, 1 equiv. KHMDS, 70 °C, 3 days; (v) 2.67 equiv. *t*BuC(O)Cl, 0.67 equiv. KHMDS, RT, 1 day. Yields are defined relative to the limiting reagent (Bu_3_SnH/Bu_3_SnOMe).

It is proposed that P_red_ hydrostannylation proceeds through a simple radical chain mechanism largely equivalent to that proposed for P_4_ (Scheme 2b).^[15]^ Interestingly, when the optimised procedure was performed using less “driving”, lower energy 455 nm LEDs, ^31^P NMR analysis of the partially converted reaction mixture showed a set of minor multiplets consistent with formation of P_7_(SnBu_3_)_3_ (see Section 1.1 in the Supporting Information).^[16]^ No analogous observation was ever made during our previous study of P_4_ hydrostannylation.^[13]^

While far from conclusive, this suggests that P_red_ hydrostannylation may proceed through initial, rate‐limiting excision of soluble, partially reduced oligomeric P_*n*_ moieties from the solid surface, followed by rapid further reduction in solution (Scheme 2b). During reaction optimisation, P_7_(SnBu_3_)_3_ was not observed for any reactions using the optimised wavelength of 365 nm, even when only partial conversions were achieved, which could indicate its faster hydrostannylation under these conditions (see Section 1.1 in the Supporting Information).

It is known from our previous work that the hydrostannylation products (Bu_3_Sn)_*x*_PH_3−*x*_ (*x*=1–3) possess reactive P−Sn and P−H bonds and can serve as a combined “P^3−^” synthon to react with suitable electrophiles and directly afford desirable organophosphorus compounds in a “one‐pot” fashion.^[13]^ Thus, after performing the optimised hydrostannylation of P_red_, addition of paraformaldehyde as a representative C‐centred electrophile in ethanol followed by quenching with HCl allowed the product tetrakis(hydroxymethyl)phosphonium chloride (THPC) to be formed exclusively and with good conversion (70 %, Section 1.2 in the Supporting Information). Upon increasing the reaction to a preparative scale (0.6 mmol), THPC could be isolated in good yield (58 %, see Scheme 2c and Sections 1.3 and 1.4 in the Supporting Information) without having required isolation or purification of any intermediates. Notably, the by‐product of this reaction, Bu_3_SnCl (**6**), could also be recovered in excellent yield (96 %) after a simple extraction procedure. This is significant because organotin derivatives can display appreciable toxicity and must be handled with commensurate care, and stoichiometric organotin waste is in principle one of the main limitations of this procedure. However, we have demonstrated previously that Bu_3_SnCl recovery allows for easy recycling of the Bu_3_Sn moiety, thus minimising organotin waste and potentially helping to mitigate against this issue.[^13^, ^17^]

Further investigations showed that the selective alkylation and acylation of the hydrostannylated phosphine mixture could also be achieved in a similar fashion from P_red_, by treating with benzyl bromide (BnBr; 45 % conversion to [Et_4_P]Br, **7 a**) or pivaloyl chloride (*t*BuC(O)Cl; 44 % conversion to *t*BuC(O))_3_P, **8 a**), respectively, in the presence of base (see Scheme 2c and Section 1.2 in the Supporting Information). Collectively, these initial results clearly demonstrate the principle of the desired, direct transformation of P_red_ into P_1_ products. Nevertheless, it was noticed that the conversions achieved from P_red_ were consistently lower than those previously achieved when starting from P_4_ (e. g., cf. 80 % isolated yield for [Bn_4_P]Br from P_4_).^[13]^ It was speculated that this could arise from the previously observed ability of the hydrostannylated monophosphine mixture (Bu_3_Sn)_*x*_PH_3−*x*_ (*x*=1–3) to scramble its H and Bu_3_Sn ligands, which should be accelerated by the much higher concentrations used to reduce P_red_.^[13]^ This would increase the fraction of gaseous PH_3_ in the mixture, which is liable to be unproductively lost during subsequent manipulations. Indeed, such PH_3_ loss has been proposed to be a limiting factor even when P_4_ is employed as the substrate.^[4]^

To attempt to mitigate this problem it was decided to investigate the selective conversion of P_red_ into the fully stannylated phosphine (Bu_3_Sn)_3_P (**4**) as a single product, as an alternative to the more complex, PH_3_‐containing mixture (Bu_3_Sn)_*x*_PH_3−*x*_ (*x*=1–3).^[5f]^ We previously found that addition of Bu_3_SnOMe prior to the hydrostannylation of P_4_ results in conversion of the initially formed P−H bonds into P−Sn bonds, and that this can be used to selectively prepare **4** in excellent yield.^[13]^ Satisfyingly, when the already‐optimised hydrostannylation of P_red_ was repeated in the presence of Bu_3_SnOMe the desired product **4** was formed seemingly quantitatively, without the need for any further reaction modifications (for further details see Section 2.1 in the Supporting Information).

It was possible to isolate product **4** from this one‐step reaction in excellent yield (86 %, see Scheme 3a and Section 2.2 in the Supporting Information). More significantly, it was confirmed that (Bu_3_Sn)_3_P (**4**) could also serve as an intermediate “P^3−^” synthon and be functionalised with suitable electrophiles in a similar fashion to the previous (Bu_3_Sn)_*x*_PH_3−*x*_ mixture.^[5f]^ For example, treatment of crude (Bu_3_Sn)_3_P generated from P_red_ directly with paraformaldehyde in ethanol followed by quenching with HCl furnished THPC (**5**) in excellent isolated yield (85 %, see Scheme 3b and Section 3.1 in the Supporting Information). This yield is appreciably higher than that obtained from hydrostannylation in the absence of Bu_3_SnOMe (Scheme 2c and see above), and is in excellent agreement with yields obtained previously using P_4_.^[13]^ Once again, Bu_3_SnCl (**6**) could also be recovered from this reaction in excellent yield (98 %) with minimal effort, for potential recycling.

**Scheme 3:**
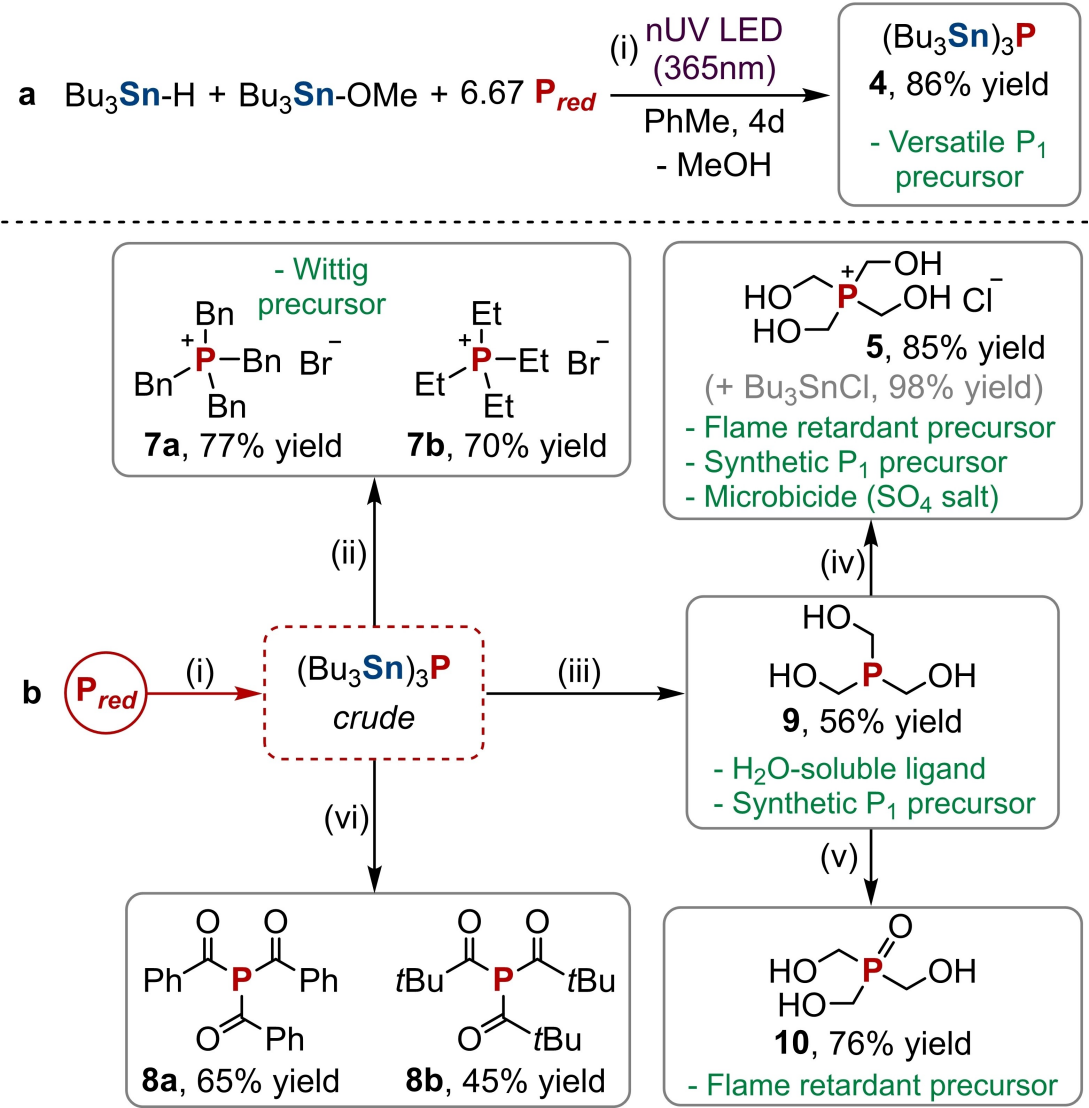
a) One‐pot synthesis of (Bu_3_Sn)_3_P directly from P_red_ using Bu_3_SnH and Bu_3_SnOMe promoted by near‐UV irradiation. b) Synthesis of P_1_ products directly from P_red_ by stannylation to (Bu_3_Sn)_3_P. (i) Stannylation of P_red_ (6.67 equiv.) with Bu_3_SnH (0.06 mmol, 1 equiv.), Bu_3_SnOMe (0.06 mmol, 1 equiv.) and PhMe (50 μL), 365 nm LEDs, RT, 4 days; (ii) preparation of phosphonium salts [R_4_P]Br from crude (Bu_3_Sn)_3_P: 6.67 equiv. RBr (R=Bn or Et), 105 °C, 2 days; (iii) preparation of THP from crude (Bu_3_Sn)_3_P: EtOH, 12.5 equiv. paraformaldehyde, RT, 16 h; (iv) preparation of THPC from crude (Bu_3_Sn)_3_P: EtOH, 8.33 equiv. paraformaldehyde, RT, 16 h, then 6.67 equiv. HCl (4.0 M in 1,4‐dioxane), RT, 2 h; (v) preparation of THPO from crude THP: PhMe/H_2_O, air, 90 °C, 16 h; vi) preparation of triacylphosphines P(C(O)R)_3_ from crude (Bu_3_Sn)_3_P: 2.67 equiv. RC(O)Cl (R=Ph or *t*Bu), RT, 2 days. Yields are defined relative to the limiting reagent (Bu_3_SnH/Bu_3_SnOMe).

Similar reactions allowed the conversion of P_red_ directly into the corresponding phosphine (HOCH_2_)_3_P (THP, **9**, by excluding the HCl quench) and phosphine oxide (HOCH_2_)_3_PO (THPO, **10**, by quenching with air) as well as the phosphonium salts [Bn_4_P]Br and [Et_4_P]Br (**7 a** and **7 b** respectively, prepared using BnBr and EtBr) and the triacylphosphines (*t*BuC(O))_3_P and (PhC(O))_3_P (**8 a** and **8 b**, respectively, prepared using *t*BuC(O)Cl and PhC(O)Cl), in generally good to excellent isolated yields (Scheme 3b and Sections 3.2–3.7 in the Supporting Information). The industrial and academic applications of these isolated products include flame retardants (**5** and **10**),[^1b^, ^18^] Wittig reagents (**7**), and chemical precursors (**9**),[^1b^, ^19^] among others. Significantly, the formation of **7** and **8** could be performed in the absence of base which contrasts with previous results where a base was necessary for functionalisation of the intermediate P−H bonds present in (Bu_3_Sn)_*x*_PH_3−*x*_ (*x*=1 or 2), thus highlighting an additional advantage of instead proceeding via (Bu_3_Sn)_3_P only.^[5f]^

From the results summarised in Scheme 3b, the scope of this new, direct P_red_ functionalisation reaction appears to closely match that of the corresponding P_4_ functionalisation. In all cases the (Bu_3_Sn)_3_P functionalisation step could be achieved using identical or near‐identical conditions to those used previously for (Bu_3_Sn)_*x*_PH_3−*x*_ functionalisation, and the isolated yields starting from P_red_ and P_4_ are generally in excellent agreement (e. g., 77 vs. 80 % for **7 a**, 76 vs. 77 % for **10**).^[13]^

Finally, as a further demonstration of the utility of this method, the synthesis of the key intermediate (Bu_3_Sn)_3_P (**4**) directly from P_red_ was investigated without the use of a glovebox. Although performing the reaction completely under air was detrimental (presumably due to sensitivity of the radical chain mechanism towards O_2_), it was found that the use of “bench” solvent, standard Schlenk techniques, and/or freeze‐pump‐thaw degassing instead of dried solvent in a glovebox led to only minor reductions in conversion (<10 %, for full details see Section 4.1 in the Supporting Information). As it is relatively air‐stable,^[20]^ (Bu_3_Sn)_3_P can also subsequently be worked up under air. Thus, without a glovebox and using only simple, easily reproducible air‐exclusion techniques, this key species could be conveniently synthesised at preparative scale and in very good isolated yield (76 %, see Section 4.2 in the Supporting Information).

We have therefore described herein the development of a practical and highly versatile new method for the direct transformation of P_red_ into a variety of useful monophosphorus compounds. This system provides access to a wide variety of product structures, including examples with significant industrial and academic relevance. Despite the relative inertness of P_red_, and unlike most other examples of productive P_red_ functionalisation, these transformations can be achieved without the need for especially powerful or elaborate reagents, or extremely rigorous inert‐atmosphere techniques. Instead, they require only simple, “familiar” reagents that can be handled in almost any standard synthetic laboratory. As a result, this method allows synthetic chemists to prepare useful P_1_ compounds by using P_red_ as a cheap and highly convenient P atom source, as an alternative to the more hazardous reagents that are currently standard (P_4_, PCl_3_, PH_3_, etc.).

## Conflict of interest

A patent covering all of the results described herein has been filed (13 February 2020) by the University of Regensburg (EP 20,157,197.3; inventors, D.J.S. and R.W.). The authors declare no other competing interests.

## Supporting information

As a service to our authors and readers, this journal provides supporting information supplied by the authors. Such materials are peer reviewed and may be re‐organized for online delivery, but are not copy‐edited or typeset. Technical support issues arising from supporting information (other than missing files) should be addressed to the authors.

Supporting Information

## Data Availability

The data that support the findings of this study are available in the supplementary material of this article.
