# Supplementary material for: Hydrostannylation of Red Phosphorus: A Convenient Route to Monophosphines
Source: Chemistry. 2022 Oct 6;28(67):e202202456. doi: 10.1002/chem.202202456 (PMC10092039; doi:10.1002/chem.202202456)
Supplement: Supplementary file 1 — Supporting Information [file CHEM-28-0-s001.pdf]

Chemistry–A European Journal

Supporting Information

Hydrostannylation of Red Phosphorus: A Convenient Route to Monophosphines

Jose Cammarata, Daniel J. Scott,* and Robert Wolf*

Table of Contents

General information 3

1. Hydrostannylation of Red Phosphorus (P_{red}) 4

1. 1. General procedure and optimisation for the hydrostannylation of P_{red} using Bu_3SnH and LED irradiation (0.06 mmol scale) 4

1. 2. General procedure for the functionalisation of the mixture $(Bu_3Sn)_xPH_{3-x}$ (x = 1–3) 8

1. 3. Synthesis and isolation of THPC (**5**) *via* hydrostannylation of P_{red} using Bu_3SnH (12 x 0.06 mmol scale) with recovery of Bu_3SnCl (**6**) 9

1. 4. Synthesis and isolation of THPC (**5**) *via* hydrostannylation of P_{red} using Bu_3SnH (0.6 mmol scale) with recovery of Bu_3SnCl (**6**) 14

2. Stannylation of Red Phosphorus (P_{red}) 15

2. 1. General procedure for the stannylation of P_{red} using Bu_3SnH and Bu_3SnOMe under near-UV LED irradiation (0.06 mmol scale) 15

2. 2. Synthesis and isolation of $(Bu_3Sn)_3P$ (**4**) 18

3. Synthesis and Isolation of Products Derived from $(Bu_3Sn)_3P$ 21

3. 1. Synthesis and isolation of THPC (**5**) *via* stannylation of P_{red} using Bu_3SnH and Bu_3SnOMe with recovery of Bu_3SnCl (**6**) 21

3. 2. Synthesis and isolation of THP (**9**) 22

3. 3. Synthesis and isolation of $(HOCH_2)_3PO$ (**10**) 25

3. 4. Synthesis and isolation of $[Bn_4P]Br$ (**7a**) 28

3. 5. Synthesis and isolation of $[Et_4P]Br$ (**7b**) 31

3. 6. Synthesis and isolation of $P(C(O)Ph)_3$ (**8a**) 34

3. 7. Synthesis and isolation of $P(C(O)tBu)_3$ (**8b**) 37

4. Stannylation of Red Phosphorus (P_{red}) without a glovebox 40

4. 1. General procedures for the stannylation of P_{red} using Bu_3SnH and Bu_3SnOMe under near-UV LED irradiation without using a glovebox 40

4. 2. Synthesis and isolation of $(Bu_3Sn)_3P$ (**4**) without using a glovebox 41

5. (Hydro)Stannylation of Red Phosphorus (P_{red}) promoted by chemical radical initiators 42

5. 1. General procedure and optimisation for the hydrostannylation of P_{red} using Bu_3SnH and AIBN (0.06 mmol scale) 42

5. 2. General procedure and optimisation for the stannylation of P_{red} using Bu_3SnH, Bu_3SnOMe and AIBN (0.06 mmol scale) 44

References 46

General information

Unless stated otherwise, all reactions and manipulations were performed under an N_2 atmosphere (< 0.1 ppm O_2, H_2O) through use of MBraun Unilab and GS MEGA Line gloveboxes and standard Schlenk line techniques. All glassware was oven-dried (160 °C) overnight prior to use. PhH was distilled from Na/benzophenone and stored over molecular sieves (3 Å). MeCN was distilled from CaH_2 and stored over molecular sieves (3 Å). *n*-Hexane was purified using an MBraun SPS-800 system and stored over K. PhMe, Et_2O and THF were purified using an MBraun SPS-800 system and stored over molecular sieves (3 Å). EtOH was degassed and dried by standing over at least three sequential batches of molecular sieves (3 Å). C_6D_6 was distilled from K and stored over molecular sieves (3 Å). CD_3CN, CD_3OD and D_2O were used without purification. All reagents and starting materials were purchased from major suppliers. Liquids were degassed (if not supplied under inert atmosphere) but were otherwise used as supplied, unless stated otherwise. Bu_3SnH was supplied containing 0.05% BHT as stabilizer and was used as received. Bu_3SnOMe was degassed and stored over molecular sieves (3 Å). BnBr, EtBr, PhC(O)Cl and *t*BuC(O)Cl were distilled, degassed, and stored over molecular sieves (3 Å). Solids were dried under vacuum (with the exception of paraformaldehyde) but otherwise used as supplied, unless stated otherwise. Red phosphorus (≥97.0%) was purchased from Sigma-Aldrich.

NMR spectra were recorded at room temperature on Bruker Avance 400 (400 MHz) spectrometers and were processed using Topspin 3.2. Chemical shifts, δ, are reported in parts per million (ppm); ^{1}H NMR and ^{13}C NMR shifts are reported relative to $SiMe_4$ and were referenced internally to residual solvent peaks, while ^{31}P NMR and ^{119}Sn shifts were referenced externally to 85 % H_3PO_4 (aq.) and $SnMe_4$ (90% in C_6D_6), respectively. Except where stated otherwise, integrals for $^{31}P\{^{1}H\}$ and ^{31}P spectra are provided for the purposes of qualitative comparison only, and should not be considered quantitatively accurate. The abbreviations s, d, t, q, m are used to indicate singlets, doublets, triplets, quartets and multiplets, respectively.

Reactions driven by light were performed using apparatus that has been described in previous publications, in which reaction vessels are illuminated from beneath by LEDs while placed in a metal block through which cooling water is constantly circulated to maintain near-ambient temperature.[1,2]

1. Hydrostannylation of Red Phosphorus (P_{red})

1.1. General procedure and optimisation for the hydrostannylation of P_{red} using Bu_3SnH and LED irradiation (0.06 mmol scale)

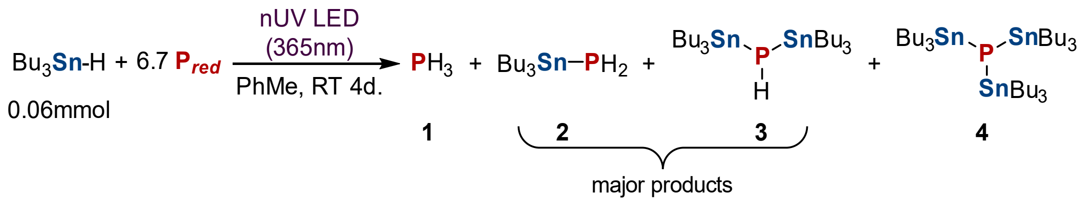

To a 10 mL, flat-bottomed, stoppered tube were added P_{red} (0.4 mmol, 12.4 mg), PhMe (50 μL) and Bu_3SnH (16.1 μL, 0.06 mmol). The tube was sealed, placed in a water-cooled block to maintain near-ambient temperature, and irradiated with UV light (365 nm, 4.3 V, 700 mA, Osram OSLON SSL 80) for 4 days (unless stated otherwise). Ph_3PO (0.02 mmol, stock solution in benzene) was subsequently added to act as an internal standard. The resulting mixture was analysed by ^{1}H, $^{31}P\{^{1}H\}$, ^{31}P, and $^{119}Sn\{^{1}H\}$ NMR spectroscopy, as shown in Figures S1-4, below.

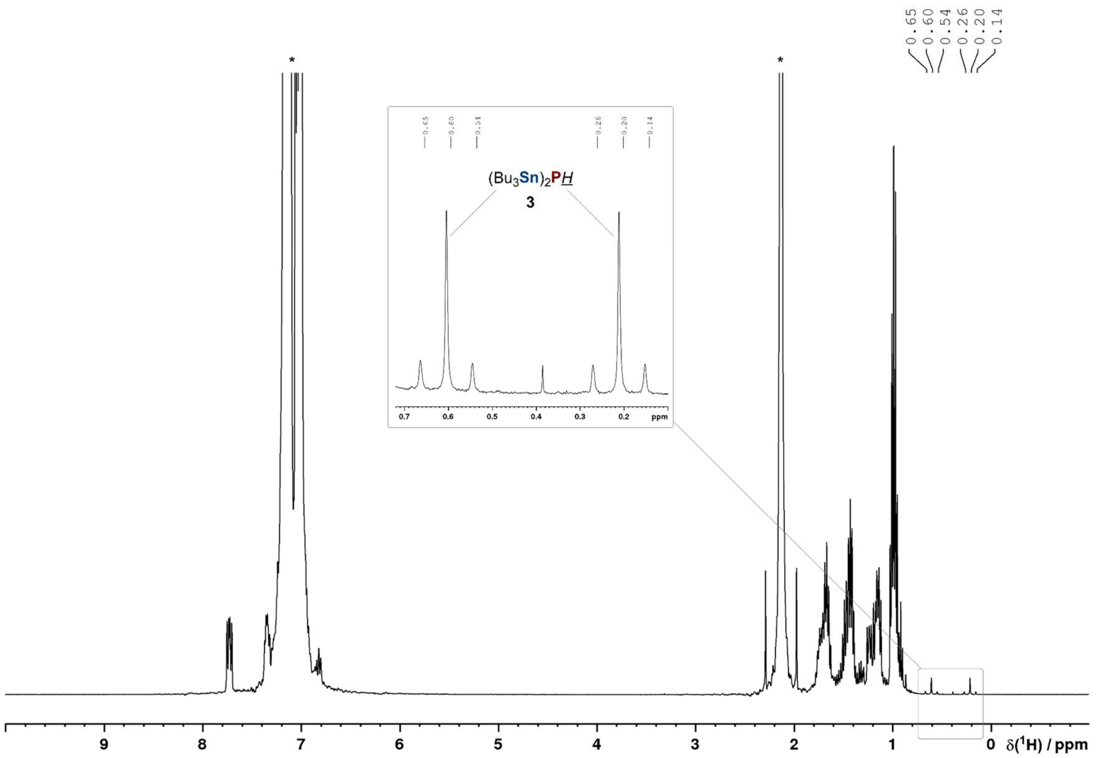

Figure S1. ^{1}H NMR spectrum for the reaction of P_{red} with Bu_3SnH (0.06 mmol) in PhMe and driven by 365 nm LED irradiation for 4 days. Solvent resonances are marked with an asterisk and are truncated for clarity. The inset shows an expansion of the doublet resonance with $^{117/119}Sn$ satellites attributed to the P*H* moiety of $(Bu_3Sn)_2PH$ (**3**).

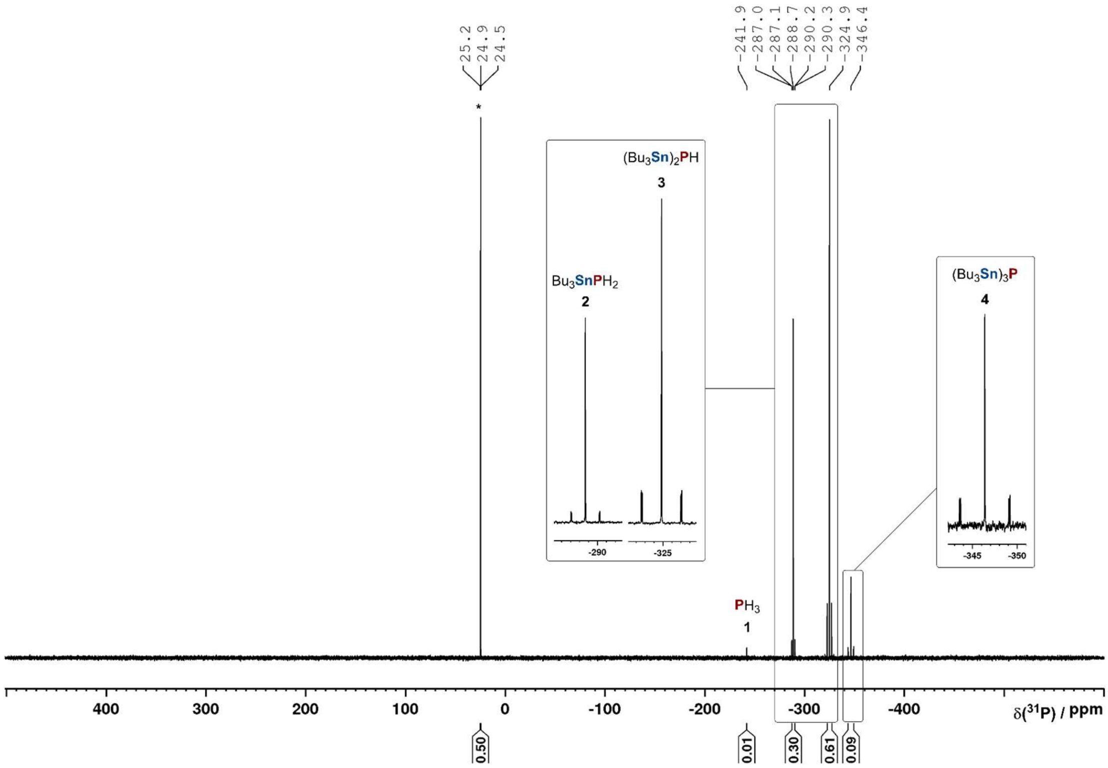

Figure S2. $^{31}P\{^1H\}$ NMR spectrum for the reaction of P_{red} with Bu_3SnH (0.06 mmol) in PhMe and driven by 365 nm LED irradiation for 4 days. The insets show expansions of the signals attributed to Bu_3SnPH_2 (**2**) and $(Bu_3Sn)_2PH$ (**3**), and to $(Bu_3Sn)_3P$ (**4**), highlighting the presence of $^{117/119}Sn$ satellites. * marks the internal standard Ph_3PO (0.02 mmol).

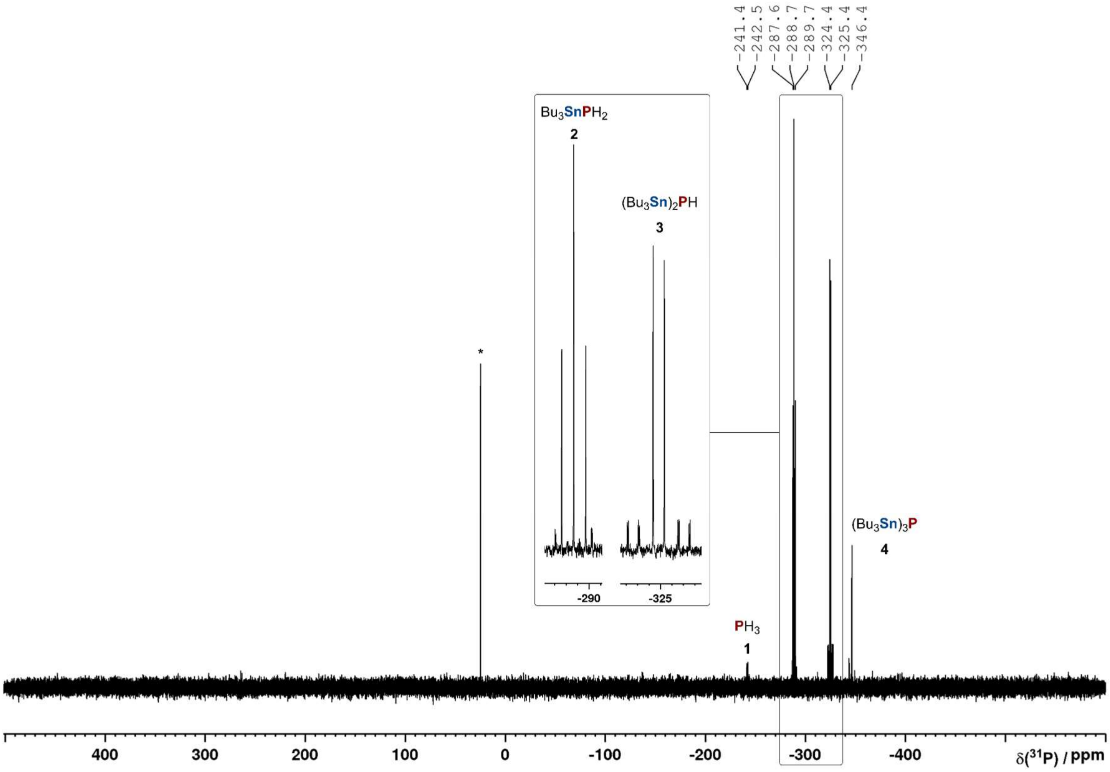

Figure S3. ^{31}P NMR spectrum for the reaction of P_{red} with Bu_3SnH (0.06 mmol) in PhMe and driven by 365 nm LED irradiation for 4 days. The insets show expansions of the signals attributed to Bu_3SnPH_2 (**2**) and $(Bu_3Sn)_2PH$ (**3**), highlighting their multiplicity due to $^1J(^{31}P\text{-}^1H)$ couplings. * marks the internal standard Ph_3PO (0.02 mmol).

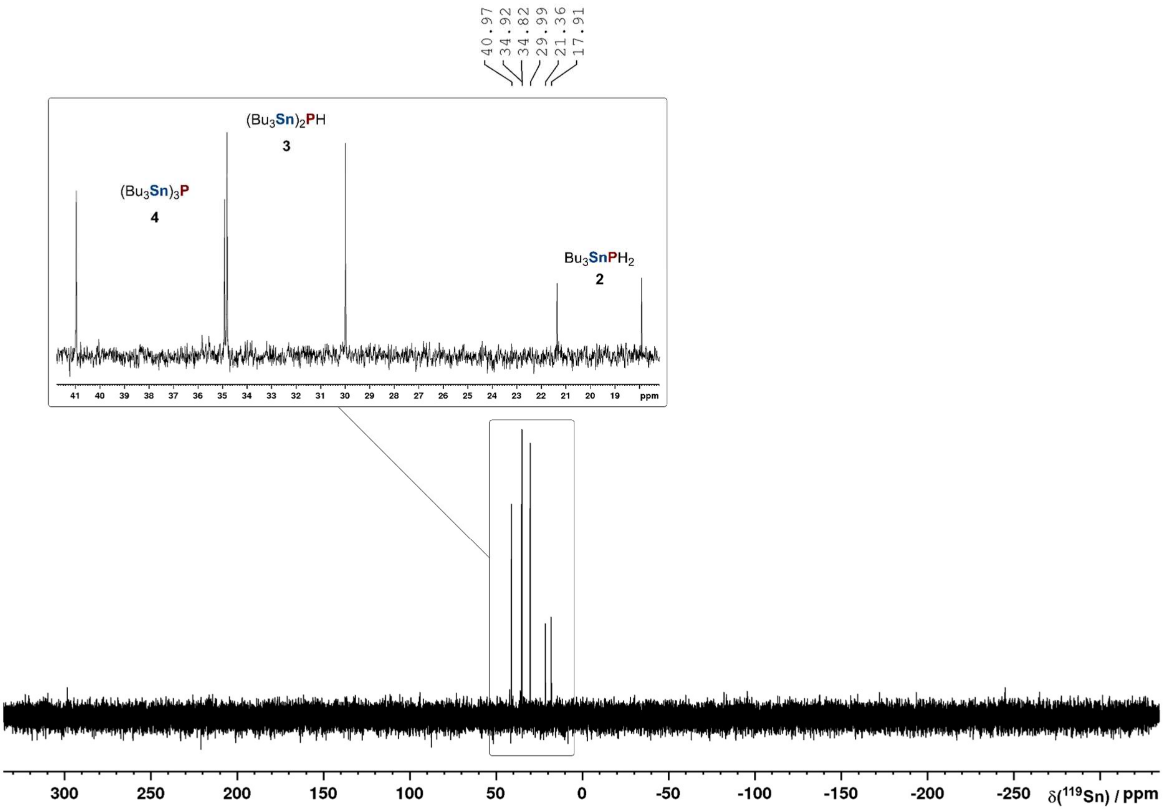

Figure S4. $^{119}Sn\{^1H\}$ NMR spectrum for the reaction of P_{red} with Bu_3SnH (0.06 mmol) in PhMe and driven by 365 nm LED irradiation for 4 days. The insets show expansions of the signals attributed to Bu_3SnPH_2 (**2**), $(Bu_3Sn)_2PH$ (**3**), and $(Bu_3Sn)_3P$ (**4**).

For reasons of experimental expediency, during the optimization of the hydrostannylation of P_{red} acquisition of quick but non-quantitative $^{31}P\{^1H\}$ NMR spectra was used to analyse each experiment and to assess the relative total conversion to **1-4**. Although this did not directly provide precise, quantitative conversions it did allow for meaningful, qualitative comparisons between experiments. Under the optimized conditions highlighted in Table S1, entry 12 (and Table S2, entry 1) full consumption of the limiting reagent Bu_3SnH was observed (assessed by ^1H NMR spectroscopy), and the $^{31}P\{^1H\}$ and $^{119}Sn\{^1H\}$ spectra suggest clean conversion to the desired products **1-4** (Figures S1-S4). It is therefore proposed that this procedure provides complete (or very nearly complete) conversion. Thus, for ease of interpretation, the integrals measured for **1-4** for all optimization experiments have been normalized relative to the value for this experiment (defined as 99%) to provide the relative conversions indicated in Table S1.

Table S1. Optimization of hydrostannylation of P*red* using Bu_3SnH and near-UV LED irradiation(365 nm)[a]

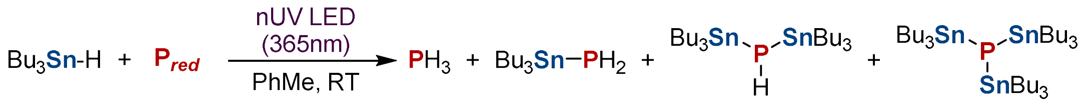

1 2 3 4

Entry	Bu_3SnH (mmol)	P*red* (mmol)	PhMe (µL)	Time (days)	Full conv. of Bu_3SnH?[b]	Relative total conv. to **1-4** (%)[c]
1[d]	0.06	0.04	500	1	X	traces
2[d]	0.06	0.04	500	3	X	20
3	0.06	0.15	500	3	X	25
4	0.06	0.4	500	3	X	50
5	0.06	0.4	500 (THF)	3	X	49
6	0.06	0.4	500 (hexane)	3	X	48
7	0.06	0.4	500 (EtOH)	3	X	18
8	0.06	0.4	250	3	X	60
9	0.06	0.4	100	3	X	70
10	0.06	0.4	50	3	X	95
11	0.06	0.4	-	3	X	36
**12**	**0.06**	**0.4**	**50**	**4**	✓	**99**
13	0.12	0.4	100	4	X	60
14	0.12	0.8	100	4	X	27
15	0.12	0.8	50	4	X	75

[a] The general procedure described in this section was modified to use the indicated amount of reactants and solvent. [b] The full consumption of Bu_3SnH was assessed by ^1H NMR spectroscopy and the disappearance of the Sn*H* resonance that would otherwise be observed at *ca.* 5 ppm. [c] Conversions were calculated by integration of the ^{31}P resonances of **1-4** relative to an internal standard, which was then normalized relative to entry 12 (defined as 99%) as described in the text above. [d] Blue LED (455 nm).

Table S2. Hydrostannylation of P*red* using Bu_3SnH and LED irradiation: screening of **LEDs**[a]

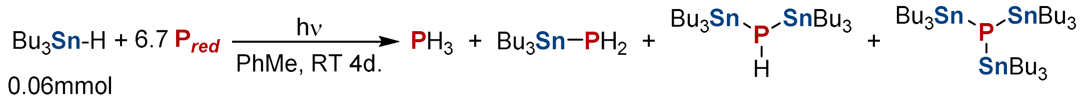

1 2 3 4

Entry	hν	Full conv. of Bu_3SnH?[b]	Relative total conv. to **1-4** (%)[c]
**1**	**365 nm**	✓	**99**
2	400 nm	X	33
3	420 nm	X	72[d]
4	455 nm	X	47[d]
5	520 nm	X	12
6	no hν, 100 °C[e]	X	7

[a] The general procedure described in this section was modified to use LEDs of the indicated wavelengths. [b] The full consumption of Bu_3SnH was assessed by ^1H NMR spectroscopy and the disappearance of the Sn*H* resonance that would otherwise be observed at *ca.* 5 ppm. [c] Conversions were calculated by integration of the ^{31}P resonances of **1-4** relative to an internal standard, which was then normalized relative to entry 1 (defined as 99%) as described in the text above. [d] Other signals were observed apart from those corresponding to **1-4**, attributed to $P_7(SnBu_3)_3$ (see Figure S5). [e] The reaction tube was wrapped in Al foil to exclude light, and heated to 100 °C for 4 days.

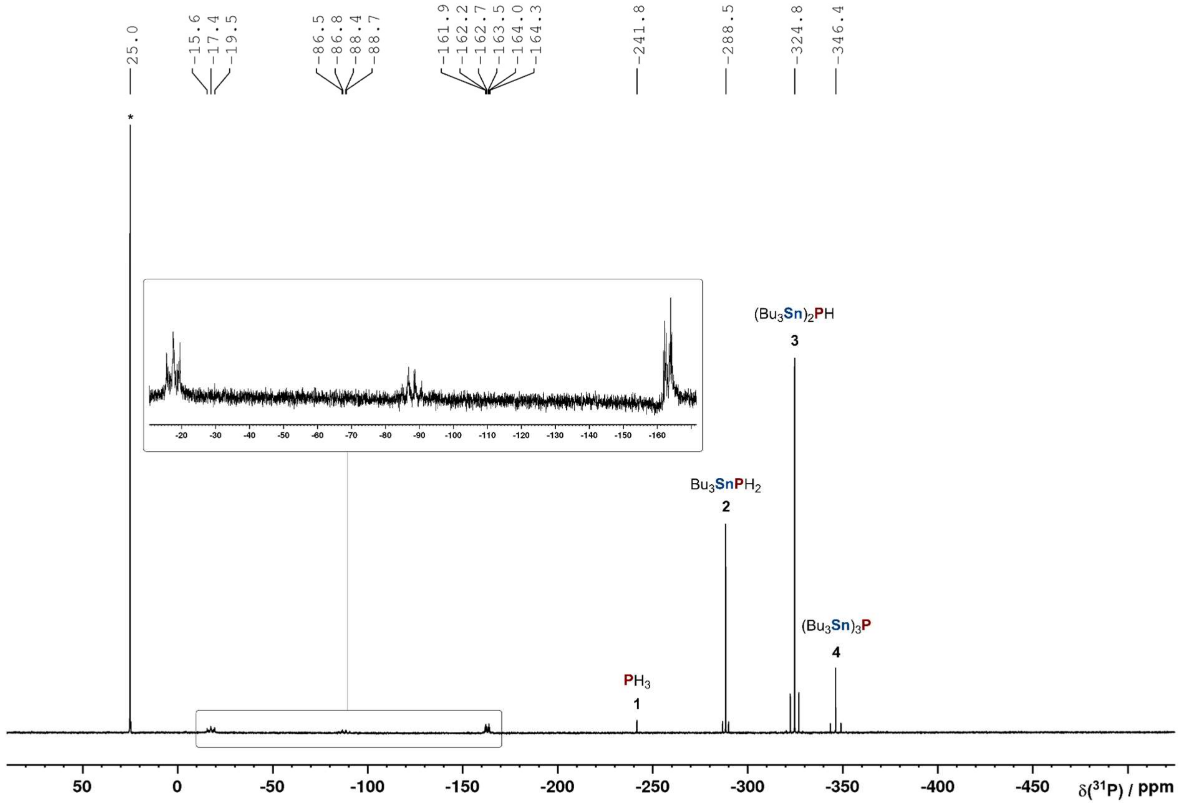

Figure S5. $^{31}P\{^1H\}$ NMR spectrum for the reaction of P_{red} with Bu_3SnH (0.06 mmol) in PhMe and driven by 455 nm LED irradiation for 4 days (Table S2, entry 4). The insets show expansion of minor signals attributed to $P_7(SnBu_3)_3$, which were assigned by comparison with those reported for $P_7(SnMe_3)_3$.[3] * marks the internal standard Ph_3PO.

1.2. General procedure for the functionalisation of the mixture $(Bu_3Sn)_xPH_{3-x}$ (x = 1–3)

The conversions of the products shown in this section were determined by a quantitative single-scan inverse-gated $^{31}P\{^1H\}$ NMR (DS = 0, D1 = 2 s) methodology that we have described previously, and whose use to quantify tertiary phosphines and quaternary phosphonium salts has previously been validated.[1]

To a 10 mL, flat-bottomed, stoppered tube were added P_{red} (0.4 mmol, 12.4 mg), PhMe (50 µL) and Bu_3SnH (16.1 µL, 0.06 mmol). The tube was sealed, placed in a water-cooled block to maintain near-ambient temperature, and irradiated with UV light (365 nm, 4.3 V, 700 mA, Osram OSLON SSL 80) for 4 days. The resulting red suspension was filtered and extracted with PhMe (0.5 mL). The resulting clear yellowish solution was treated with the corresponding electrophiles as follows:

***2.1** <u>Reactivity toward paraformaldehyde:</u>* Volatiles were removed under vacuum. EtOH (0.5 mL) and paraformaldehyde (15.0 mg, 0.5 mmol) were added to the oily residue, and the resulting suspension was stirred at room temperature for 16 h. The mixture was frozen in a liquid-nitrogen bath, and HCl (4.0 M in 1,4-dioxane, 100 µL, 0.4 mmol) was added. After thawing, the reaction mixture was stirred at room temperature for 2 h. Ph_3PO (0.02 mmol, stock solution in benzene) was subsequently added to act as an internal standard.

$^{31}P\{^1H\}$ NMR analysis of the resulting mixture showed the exclusive formation of THPC (**5**) with 70% conversion.

2.2 Reactivity toward benzyl bromide: Benzyl bromide (71.3 µL, 0.6 mmol) and KHMDS (12.0 mg, 0.06 mmol) were added to the yellowish solution, and heated to 70 °C with stirring for 3 days. After cooling to room temperature, Ph_3PO (0.02 mmol, stock solution in benzene) was subsequently added to act as an internal standard. $^{31}P\{^1H\}$ NMR analysis of the resulting mixture showed the formation of $[Bn_4P]Br$ (**7a**) as the main product with 45 % conversion.

2.2 Reactivity toward pivaloyl chloride: *t*BuC(O)Cl (19.6 µL, 0.16 mmol) and KHMDS (8.0 mg, 0.04 mmol) were added to the yellowish solution, and stirred at room temperature for 1 days. Ph_3PO (0.02 mmol, stock solution in benzene) was subsequently added to act as an internal standard. $^{31}P\{^1H\}$ NMR analysis of the resulting mixture showed the exclusive formation of $P(C(O)tBu)_3$ (**8a**) with 44 % conversion.

1.3. Synthesis and isolation of THPC (5) *via* hydrostannylation of P_{red} using Bu_3SnH (12 x 0.06 mmol scale) with recovery of Bu_3SnCl (6)

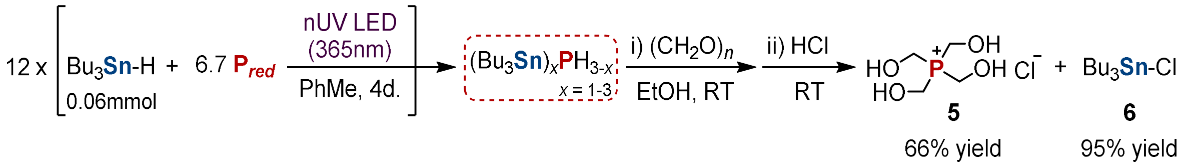

To provide sufficient material for reliable yield determination, a total of twelve reactions were performed in parallel using the following procedure: To a 10 mL, flat-bottomed, stoppered tube were added P_{red} (0.4 mmol, 12.4 mg), PhMe (50 µL) and Bu_3SnH (16.1 µL, 0.06 mmol). The tube was sealed, placed in a water-cooled block to maintain near-ambient temperature, and irradiated with UV light (365 nm, 4.3 V, 700 mA, Osram OSLON SSL 80) for 4 days. The twelve reactions were then combined in a 100 mL Schlenk using PhMe (3 x 0.5 mL) to transfer and wash each tube. The combined red suspension was filtered and the remaining solid extracted with additional PhMe (2 x 10 mL), followed by removal of volatiles under vacuum. EtOH (10 mL) and paraformaldehyde (180.2 mg, 6.0 mmol) were added to the oily residue, and the resulting suspension was stirred at room temperature for 16 h. The mixture was frozen in a liquid-nitrogen bath, and HCl (4.0 M in 1,4-dioxane, 1.2 mL, 4.8 mmol) was added. After thawing, the reaction mixture was stirred at room temperature for 2 h. Volatiles from the resulting yellowish suspension were removed under vacuum. The remaining oily solid residue was triturated with Et_2O (10 mL) overnight, filtered and washed with further Et_2O (2 x 5 mL). The resulting white solid was then again dissolved in EtOH (10 mL). Following filtration and removal of volatiles under vacuum, the desired product (**5**) was obtained as a white solid (60.0 mg, 66%).

The combined Et_2O washes from the above reaction were dried under vacuum to afford Bu_3SnCl (**6**) as a pale yellow oil (223 mg, 95%).

Spectroscopic data of THPC (**5**):

^{1}H NMR (400 MHz, 300 K, D_2O) : δ = 4.67 ppm (d, $^{2}J(^{31}P\text{-}^{1}H)$ = 1.8 Hz).

$^{31}P\{^{1}H\}$ NMR (121 MHz, 300 K, D_2O) : δ = 27.0 ppm (s).

^{31}P NMR (121 MHz, 300 K, D_2O) : δ = 27.0 ppm (s).

$^{13}C\{^{1}H\}$ NMR (101 MHz, 300 K, D_2O): δ = 49.1 ppm (d, $^{1}J(^{31}P\text{-}^{13}C)$ = 51.2 Hz).

NMR data are consistent with our previous report.[4]

Spectroscopic data of recovered Bu_3SnCl (**6**):

^{1}H NMR (400 MHz, 300 K, $CDCl_3$) : δ = 1.79-1.51 (2H, m), 1.41-1.9 (4H, m), 0.92 ppm (3H, t, $^{3}J(^{1}H\text{-}^{1}H)$ = 7.3 Hz).

$^{119}Sn\{^{1}H\}$ NMR (149 MHz, 300 K, $CDCl_3$) : δ = 157.2 ppm.

$^{13}C\{^{1}H\}$ NMR (101 MHz, 300 K, $CDCl_3$): δ = 27.9 (s), 26.9 (s), 17.6 (s), 13.6 ppm (s).

NMR data are consistent with our previous report.[4]

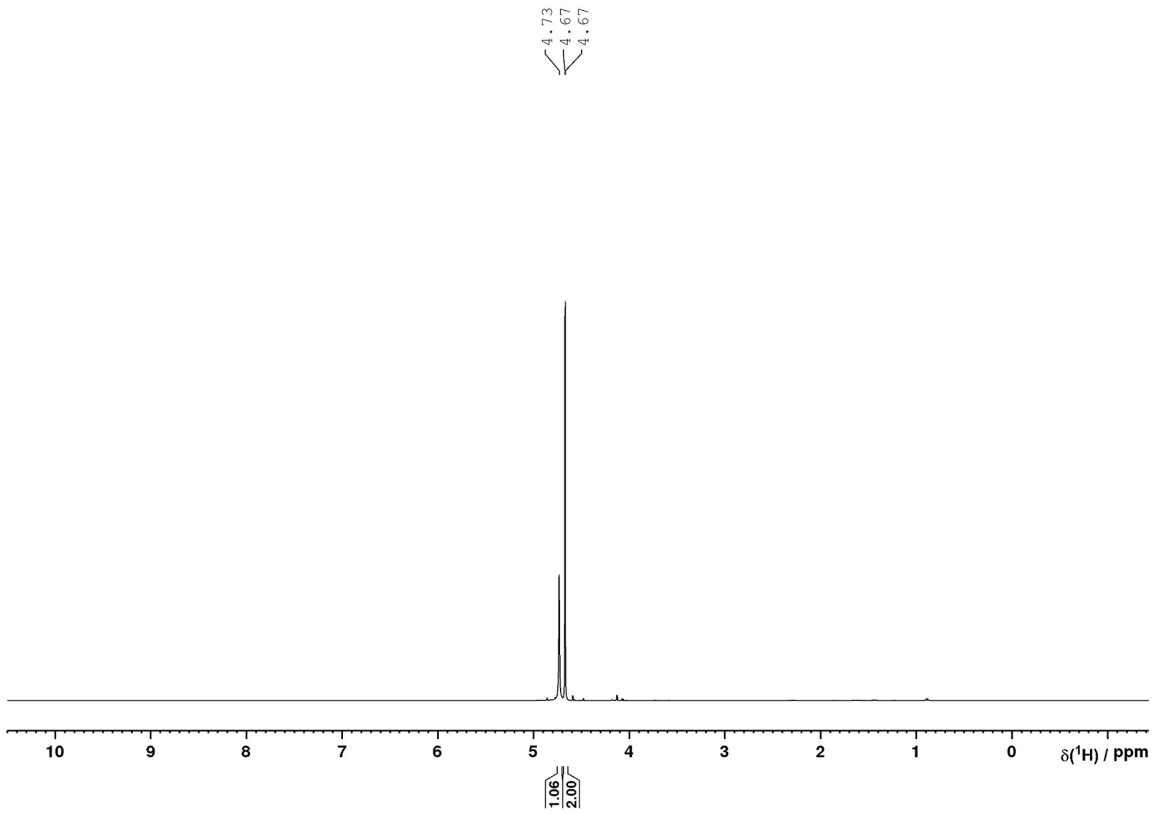

Figure S6. ^{1}H NMR spectrum of THPC (**5**) in D_2O.

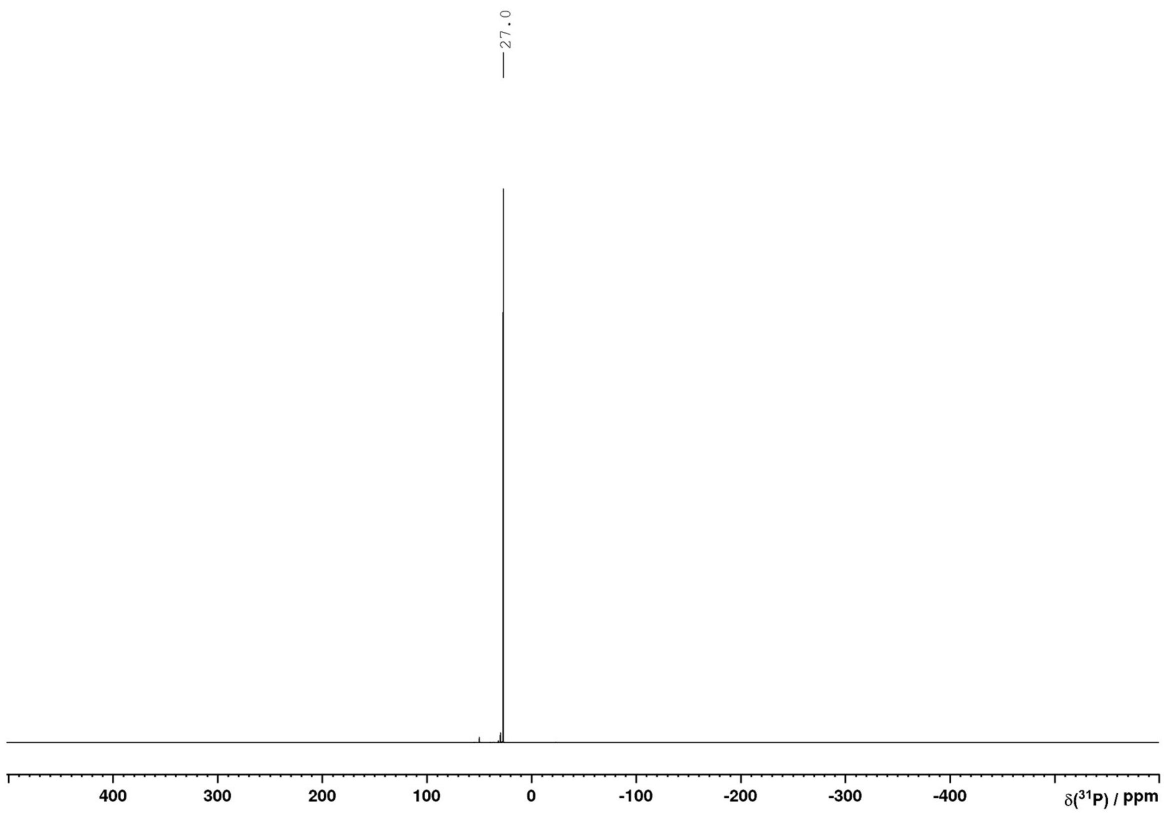

Figure S7. $^{31}P\{^{1}H\}$ NMR spectrum of THPC (**5**) in D_2O.

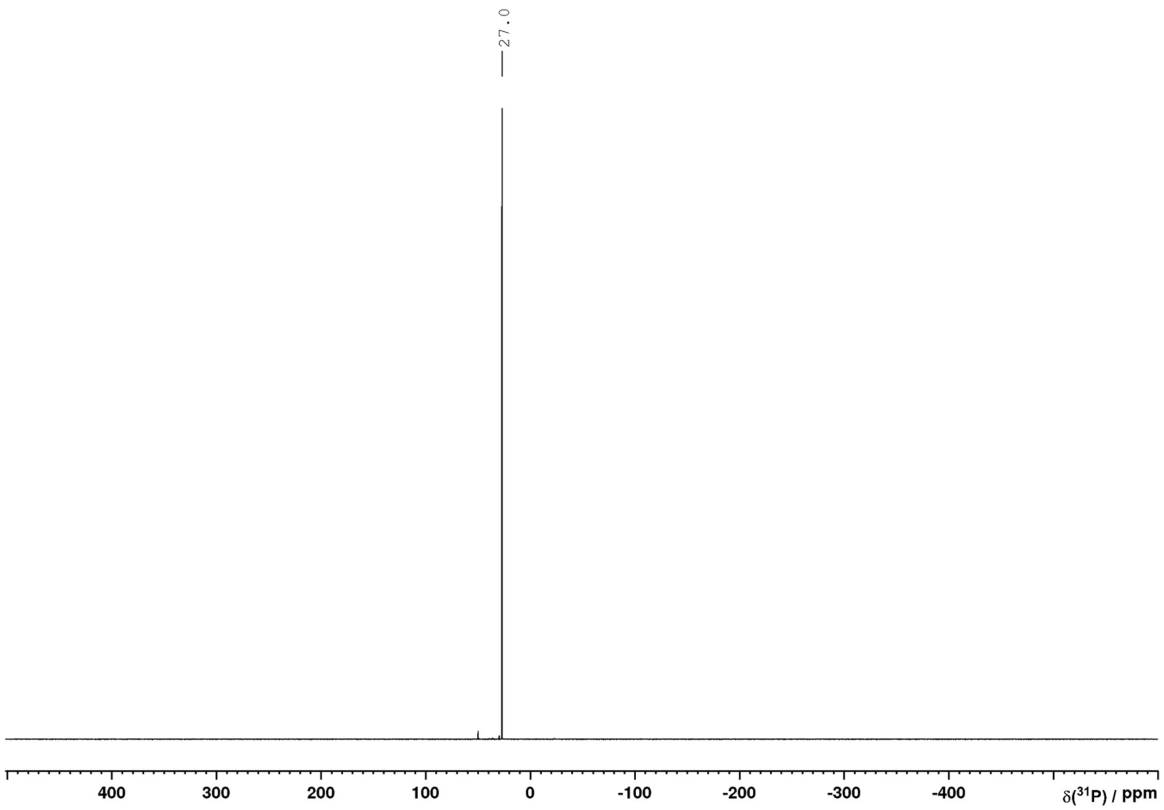

Figure S8. ^{31}P NMR spectrum of THPC (**5**) in D_2O.

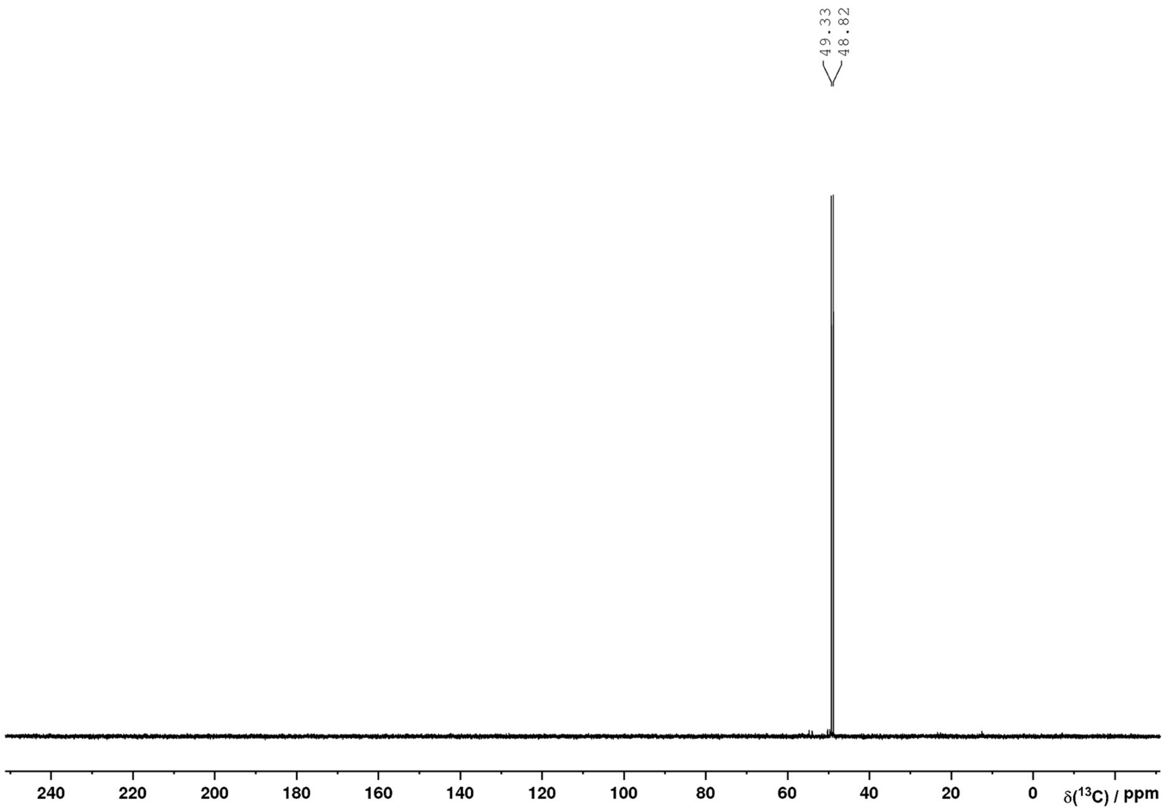

Figure S9. $^{13}C\{^{1}H\}$ NMR spectrum of THPC (**5**) in D_2O.

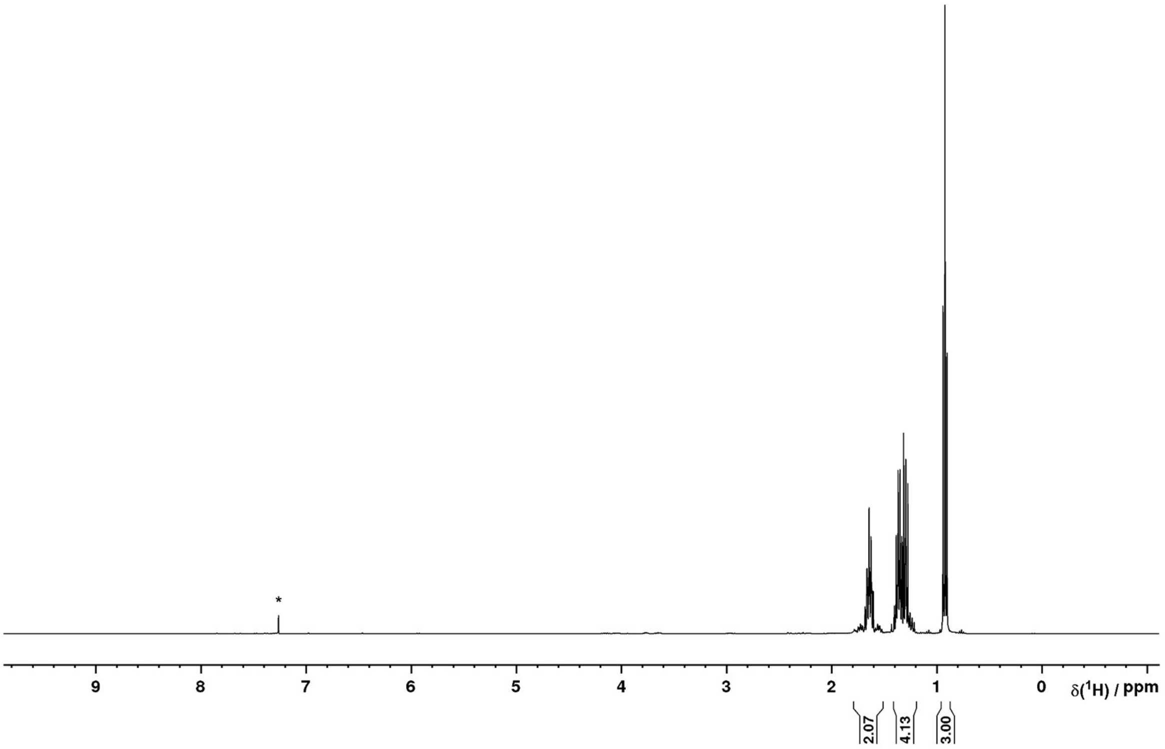

Figure S10. ^{1}H NMR spectrum of Bu_3SnCl (**6**) in $CDCl_3$ (*).

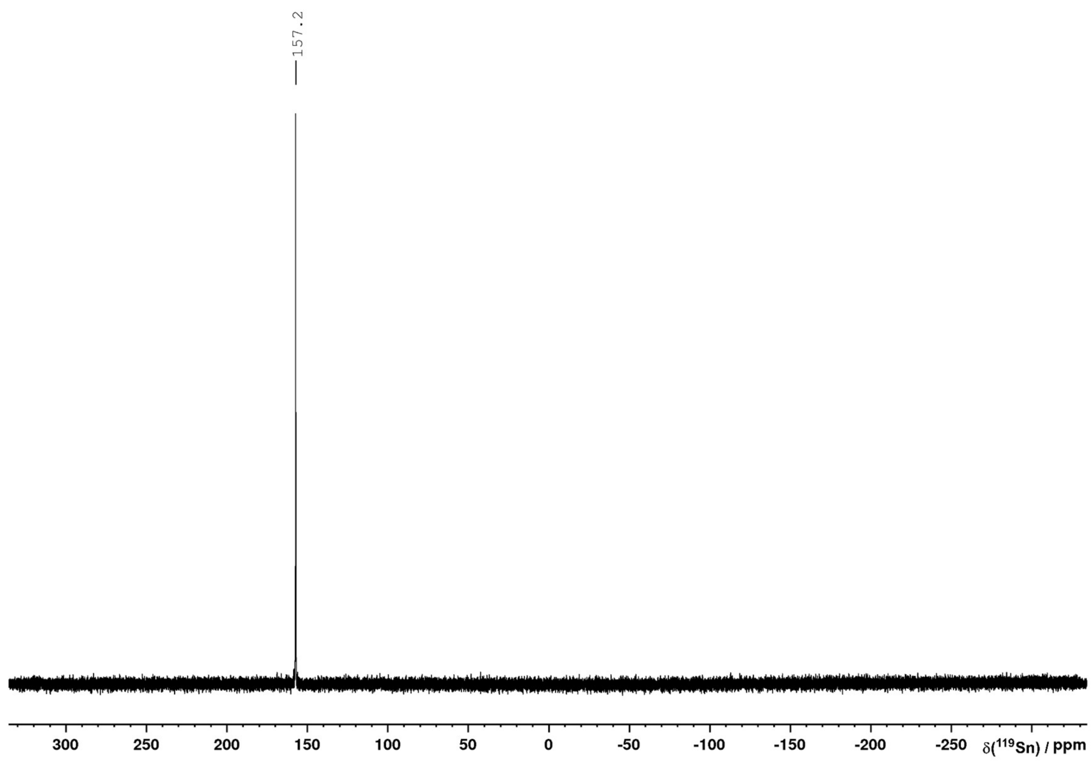

Figure S11. $^{119}Sn\{^{1}H\}$ NMR spectrum of Bu_3SnCl (**6**) in $CDCl_3$.

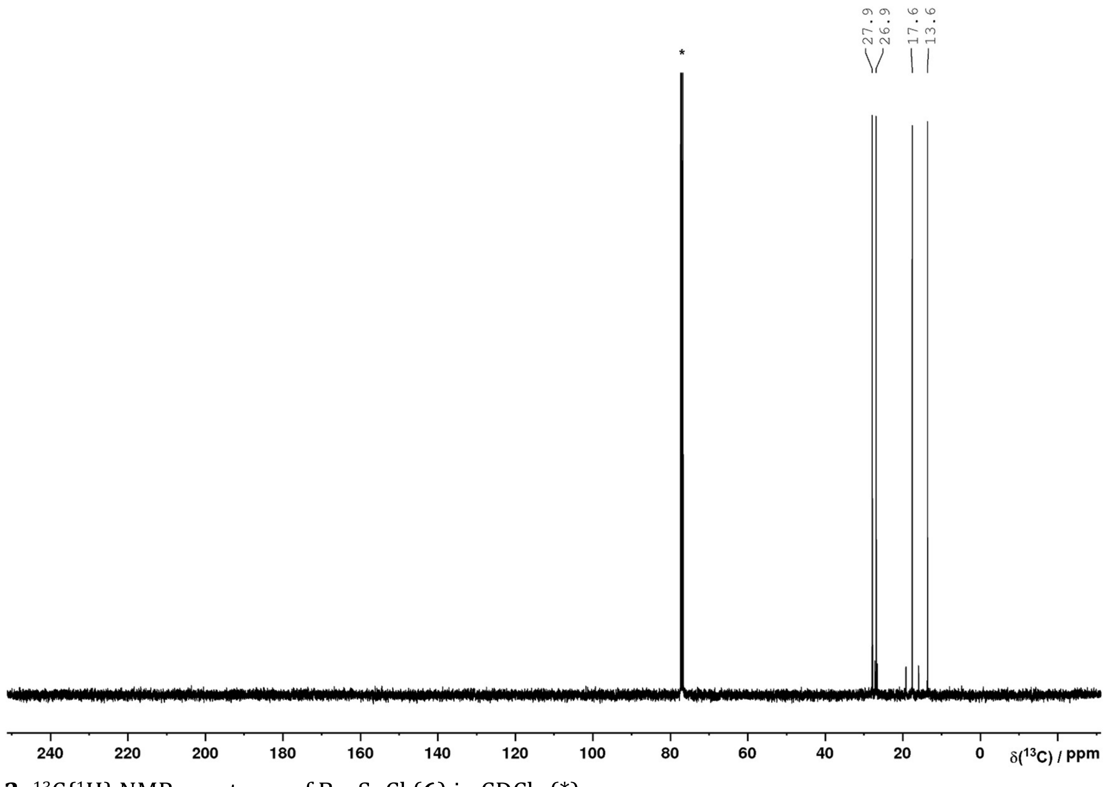

Figure S12. $^{13}C\{^{1}H\}$ NMR spectrum of Bu_3SnCl (**6**) in $CDCl_3$ (*).

1.4. Synthesis and isolation of THPC (5) *via* hydrostannylation of P_{red} using Bu_3SnH (0.6 mmol scale) with recovery of Bu_3SnCl (6)

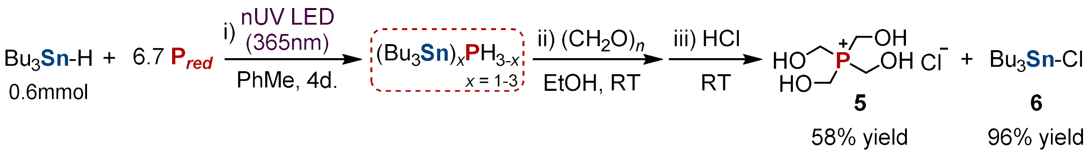

To a 50 mL, flat-bottomed, stoppered tube were added P_{red} (4.0 mmol, 124 mg), PhMe (250 µL) and Bu_3SnH (162 µL, 0.6 mmol). The tube was sealed, placed in a water-cooled block to maintain near-ambient temperature, and irradiated with UV light (365 nm, 14 V, 700 mA, Osram OSLON SSL 80) for 4 days. The red suspension was filtered and washed with additional PhMe (3 x 15 mL), followed by removal of volatiles under vacuum. EtOH (10 mL) and paraformaldehyde (150.1 mg, 5.0 mmol) were added to the oily residue, and the resulting suspension was stirred at room temperature for 18 h. The mixture was frozen in a liquid-nitrogen bath, and HCl (4.0 M in 1,4-dioxane, 1.0 mL, 4.0 mmol) was added. After thawing, the reaction mixture was stirred at room temperature for 2 h. Volatiles from the resulting yellowish suspension were removed under vacuum. The remaining oily solid residue was triturated with Et_2O (10 mL) overnight, filtered and washed with further Et_2O (2 x 10 mL). The resulting white solid was then again dissolved in EtOH (10 mL). Following filtration and removal of volatiles under vacuum, the desired product (**5**) was obtained as a white solid (44.0 mg, 58%).

The combined Et_2O washes from the above reaction were dried under vacuum to afford Bu_3SnCl (**6**) as a pale yellow oil (187 mg, 96%).

NMR data are identical to those given in section 1.3.

2. Stannylation of Red Phosphorus (P_{red})

2.1. General procedure for the stannylation of P_{red} using Bu_3SnH and Bu_3SnOMe under near-UV LED irradiation (0.06 mmol scale)

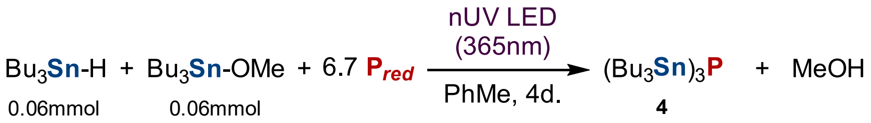

To a 10 mL, flat-bottomed, stoppered tube were added P_{red} (0.4 mmol, 12.4 mg), PhMe (50 μL), Bu_3SnH (16.1 μL, 0.06 mmol) and Bu_3SnOMe (17.3 μL, 0.06 mmol). The tube was sealed, placed in a water-cooled block to maintain near-ambient temperature, and irradiated with UV light (365 nm, 4.3 V, 700 mA, Osram OSLON SSL 80) for 4 days (unless stated otherwise). Ph_3PO (0.02 mmol, stock solution in benzene) was subsequently added to act as an internal standard. The resulting mixture was analysed by ^1H, $^{31}P\{^1H\}$, and ^{31}P NMR spectroscopy, as shown in Figures S13 and S14, below.

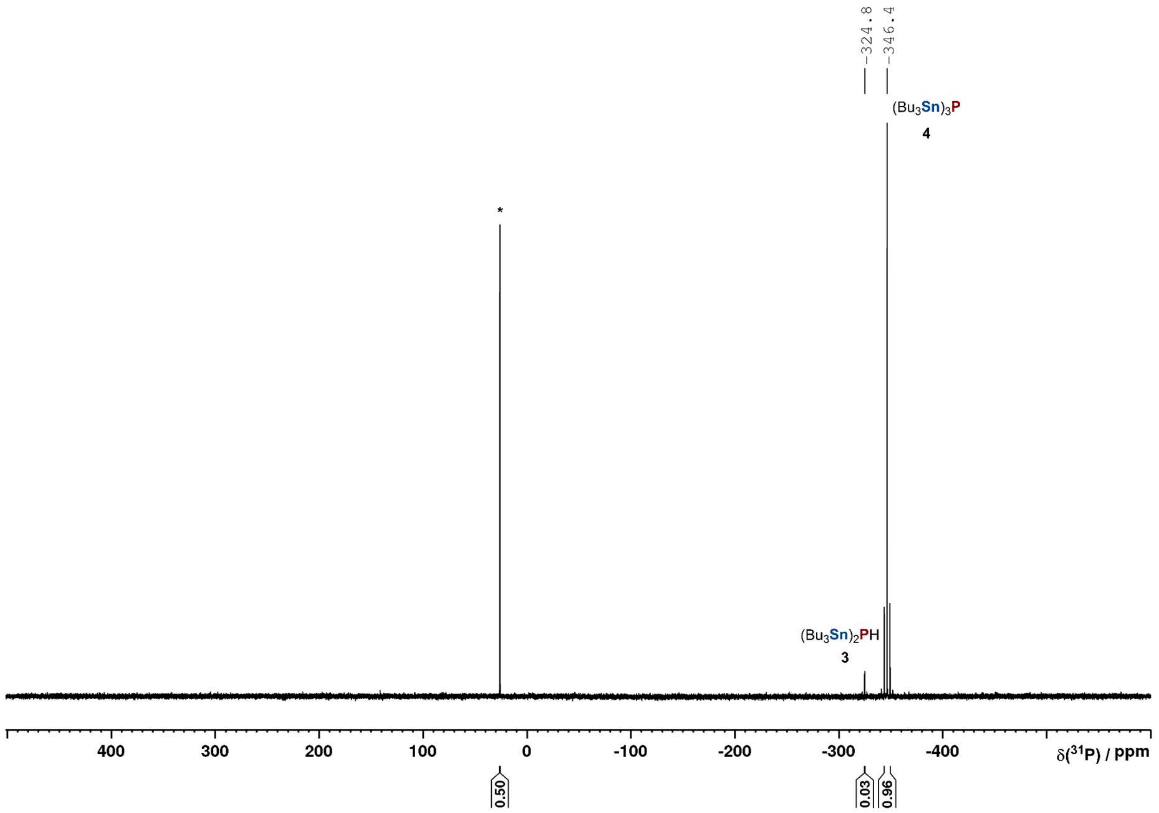

Figure S13. $^{31}P\{^1H\}$ NMR spectrum for the reaction of P_{red} with Bu_3SnH (0.06 mmol) and Bu_3SnOMe (0.06 mmol) in PhMe and driven by 365 nm LED irradiation for 4 days.* marks the internal standard Ph_3PO (0.02 mmol).

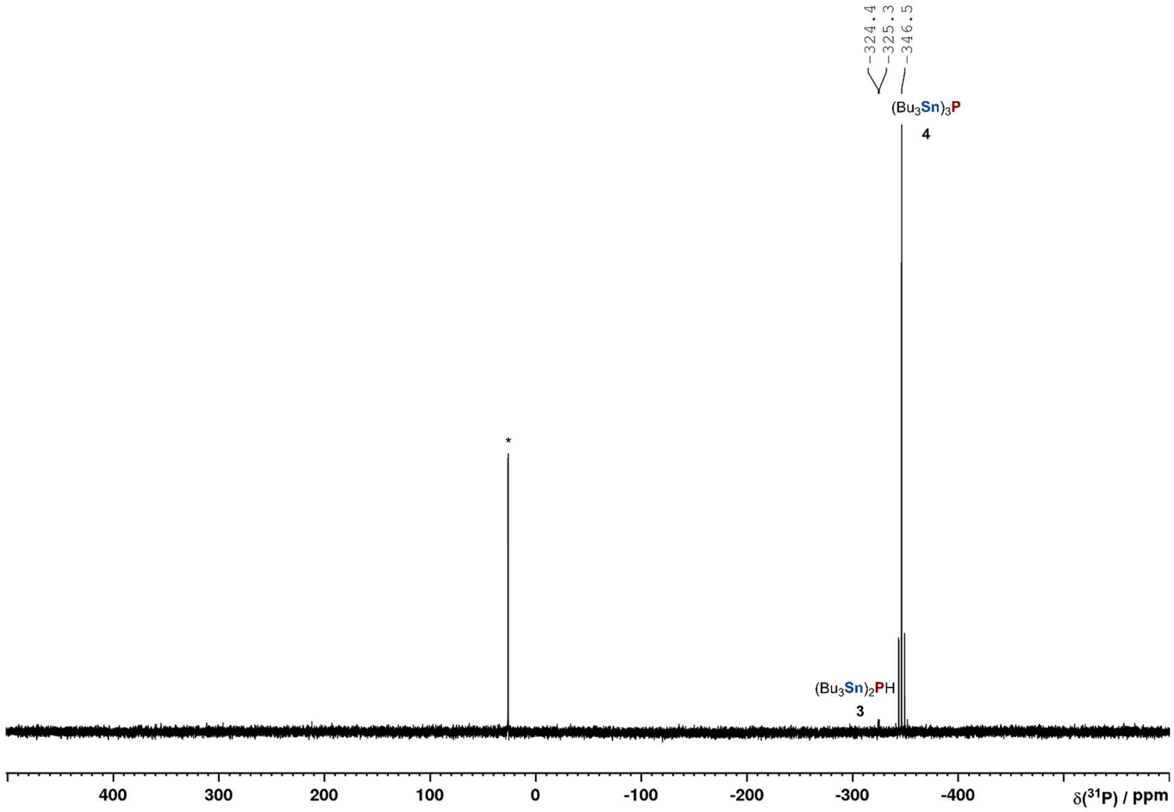

Figure S14. ^{31}P NMR spectrum for the reaction of P_{red} with Bu_3SnH (0.06 mmol) and Bu_3SnOMe (0.06 mmol) in PhMe and driven by 365 nm LED irradiation for 4 days.* marks the internal standard Ph_3PO (0.02 mmol).

Table S3. Stannylation of P_{red} using Bu_3SnH and Bu_3SnOMe under LED irradiation: screening of **solvents**[a]

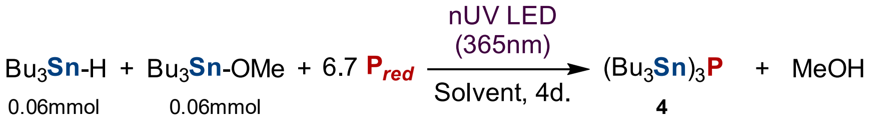

Entry	Solvent	Full conv. of Bu_3SnH?[b]	Conv. to **4** (%)[c]
1	PhMe	✓	96
2	THF	✓	91
3	Hexane	✓	90
4	EtOH	✓	75
5	Acetone	X	81
6	Acetonitrile	X	75

[a] The general procedure described in this section was modified to use the indicated solvents. [b] The full consumption of Bu_3SnH was assessed by ^{1}H NMR spectroscopy and the disappearance of the Sn*H* resonance that would otherwise be observed at *ca.* 5 ppm. [c] Conversions were calculated by integration of the ^{31}P resonance of **4** relative to an internal standard, which was then normalized relative to Table S1, entry 12 (defined as 99%) as described in section 1.1.

Table S4. Stannylation of P_{red} using Bu_3SnH and Bu_3SnOMe under near UV LED irradiation: screening of **LEDs**[a]

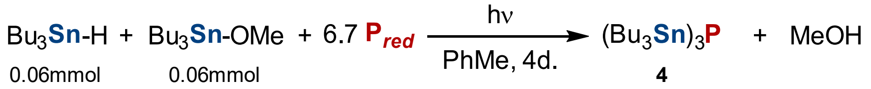

Entry	hν	Full conv. of Bu_3SnH?[b]	Conv. to **4** (%)[c]
1	365 nm	✓	96
2	400 nm	X	22
3	420 nm	X	50[d]
4	455 nm	X	28[d]
5	520 nm	X	7
6	no hν, 100 °C[e]	X	3

[a] The general procedure described in this section was modified to use LEDs of the indicated wavelengths. [b] The full consumption of Bu_3SnH was assessed by ^1H NMR spectroscopy and the disappearance of the Sn*H* resonance that would otherwise be observed at *ca.* 5 ppm. [c] Conversions were calculated by integration of the ^{31}P resonance of **4** relative to an internal standard, which was then normalized relative to Table S1, entry 12 (defined as 99%) as described in section 1.1. [d] Other signals were observed apart from the one corresponding to **4**, attributed to $P_7(SnBu_3)_3$ (see Figure S15). [e] The reaction tube was wrapped in Al foil to exclude light, and heated to 100 °C for 4 days.

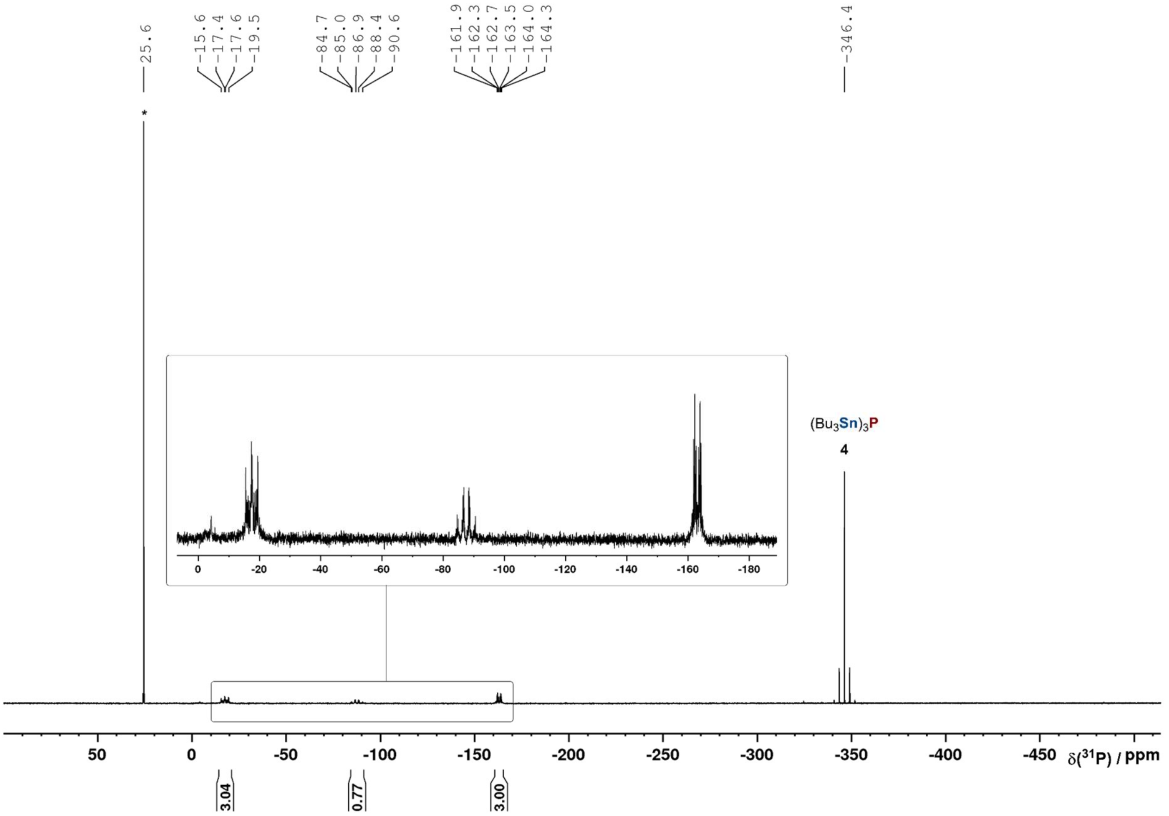

Figure S15. $^{31}P\{^1H\}$ NMR spectrum for the reaction of P_{red} with Bu_3SnH (0.06 mmol) and Bu_3SnOMe (0.06 mmol) in PhMe and driven by 455 nm LED irradiation for 4 days (Table S4, entry 4). The insets show expansion of minor signals attributed to $P_7(SnBu_3)_3$, which were assigned by comparison with those reported for $P_7(SnMe_3)_3$.[3] * marks the internal standard Ph_3PO.

2.2. Synthesis and isolation of $(Bu_3Sn)_3P$ (4)

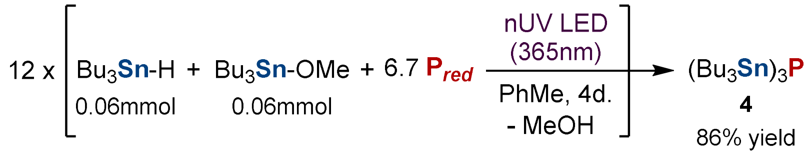

To provide sufficient material for reliable yield determination, a total of twelve reactions were performed in parallel using the following procedure: To a 10 mL, flat-bottomed, stoppered tube were added P_{red} (0.4 mmol, 12.4 mg), PhMe (50 µL), Bu_3SnH (16.1 µL, 0.06 mmol) and Bu_3SnOMe (17.3 µL, 0.06 mmol). The tube was sealed, placed in a water-cooled block to maintain near-ambient temperature, and irradiated with UV light (365 nm, 4.3 V, 700 mA, Osram OSLON SSL 80) for 4 days. The twelve reactions were then combined in a 100 mL Schlenk using PhMe (3 x 0.5 mL) to transfer and wash each tube. The combined red suspension was filtered and the remaining solid extracted with additional PhMe (2 x 10 mL), followed by removal of volatiles under vacuum. After distillation of the resulting yellowish oil under vacuum (*ca.* 105 °C, 10^{-5} mbar), the product (**4**) was obtained as a colourless oil (371.8 mg, 86%).

^{1}H NMR (400 MHz, 300 K, C_6D_6): δ = 1.84-1.63 (2H, m), 1.53-1.40 (2H, m), 1.13-1.33 (2H, m), 0.99 ppm (3H, t, $^{3}J(^{1}H-^{1}H)$ = 7.3 Hz).

$^{31}P\{^{1}H\}$ NMR (121 MHz, 300 K, C_6D_6): δ = –346.5 ppm (s).

^{31}P NMR (121 MHz, 300 K, C_6D_6): δ = –346.5 ppm (s).

$^{119}Sn\{^{1}H\}$ (149 MHz, 300 K, C_6D_6) δ = 37.5 ppm (d, $^{1}J(^{31}P-^{119}Sn)$ = 912 Hz, $^{2}J(^{119}Sn-^{117}Sn)$ = 280 Hz).

$^{13}C\{^{1}H\}$ NMR (101 MHz, 300 K, C_6D_6): δ = 30.0 (d, $J(^{31}P-^{1}H)$ = 1.4 Hz), 28.0 (s), 15.2 (d, $J(^{31}P-^{1}H)$ = 3.8 Hz), 13.9 ppm (s).

NMR data are consistent with our previous report.[4]

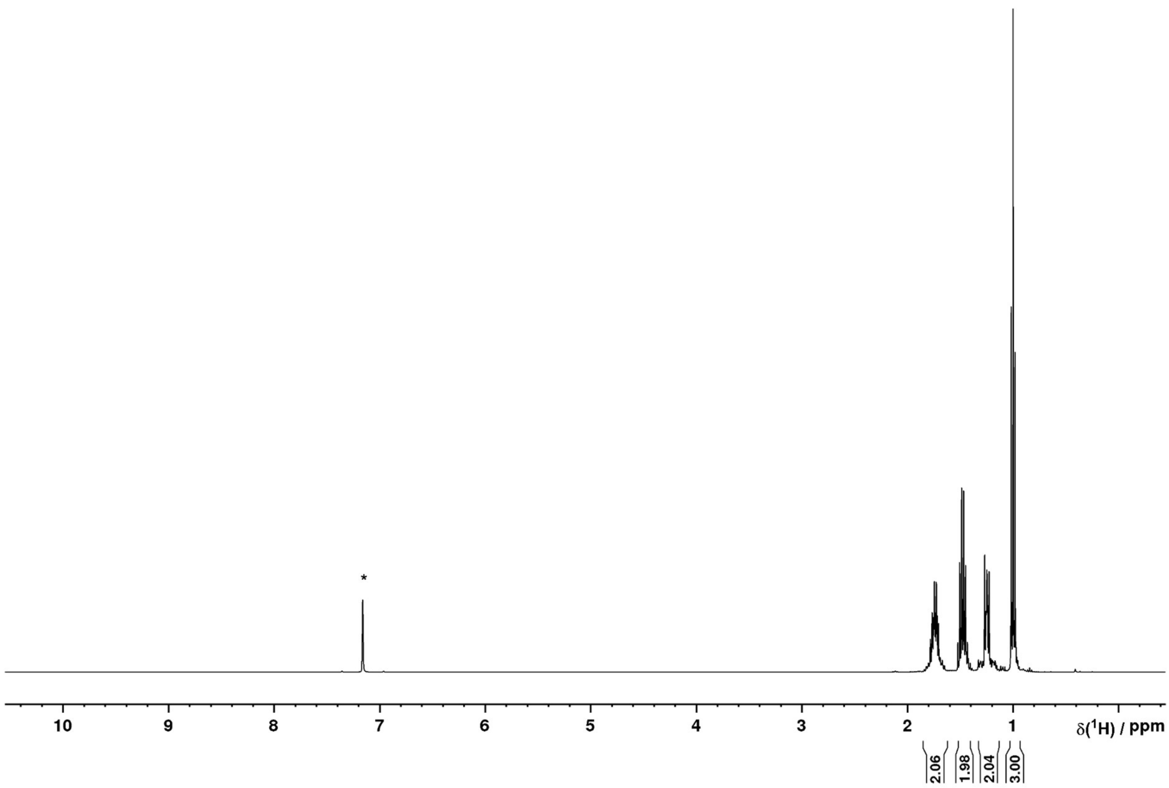

Figure S16. ^{1}H NMR spectrum of $(Bu_3Sn)_3P$ (**4**) in C_6D_6 (*).

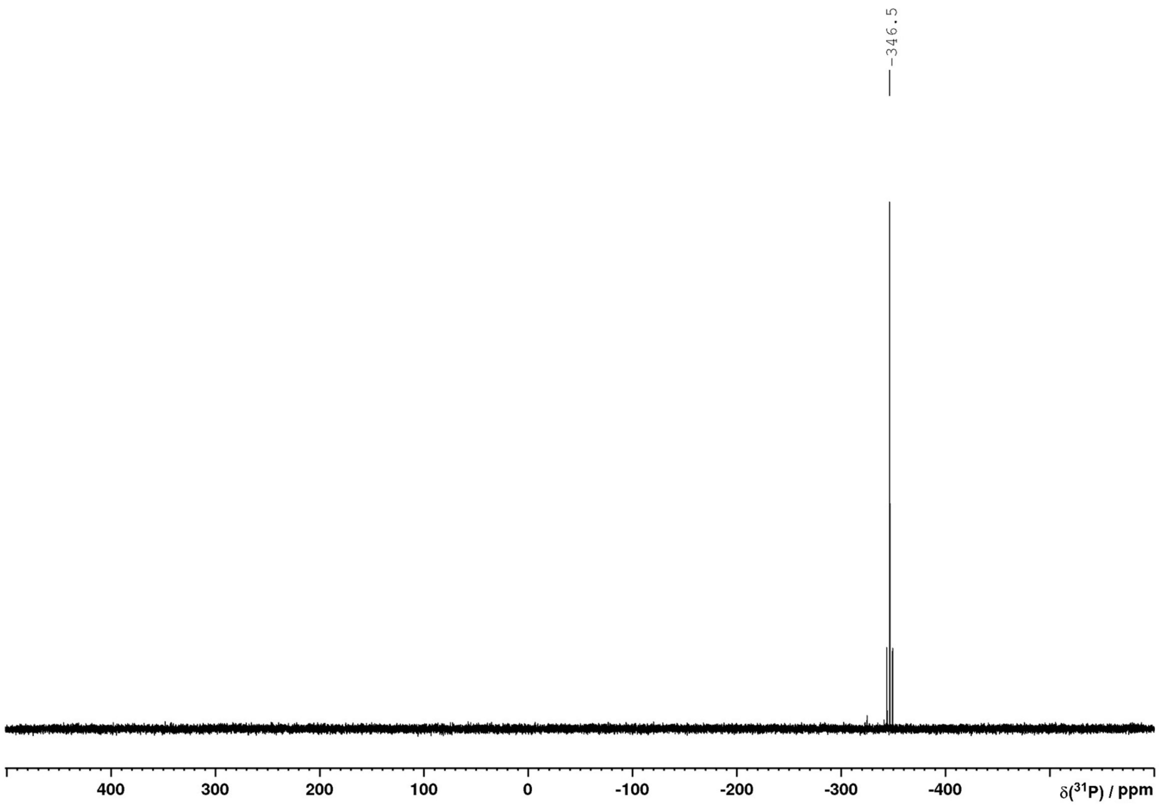

Figure S17. $^{31}P\{^{1}H\}$ NMR spectrum of $(Bu_3Sn)_3P$ (**4**) in C_6D_6.

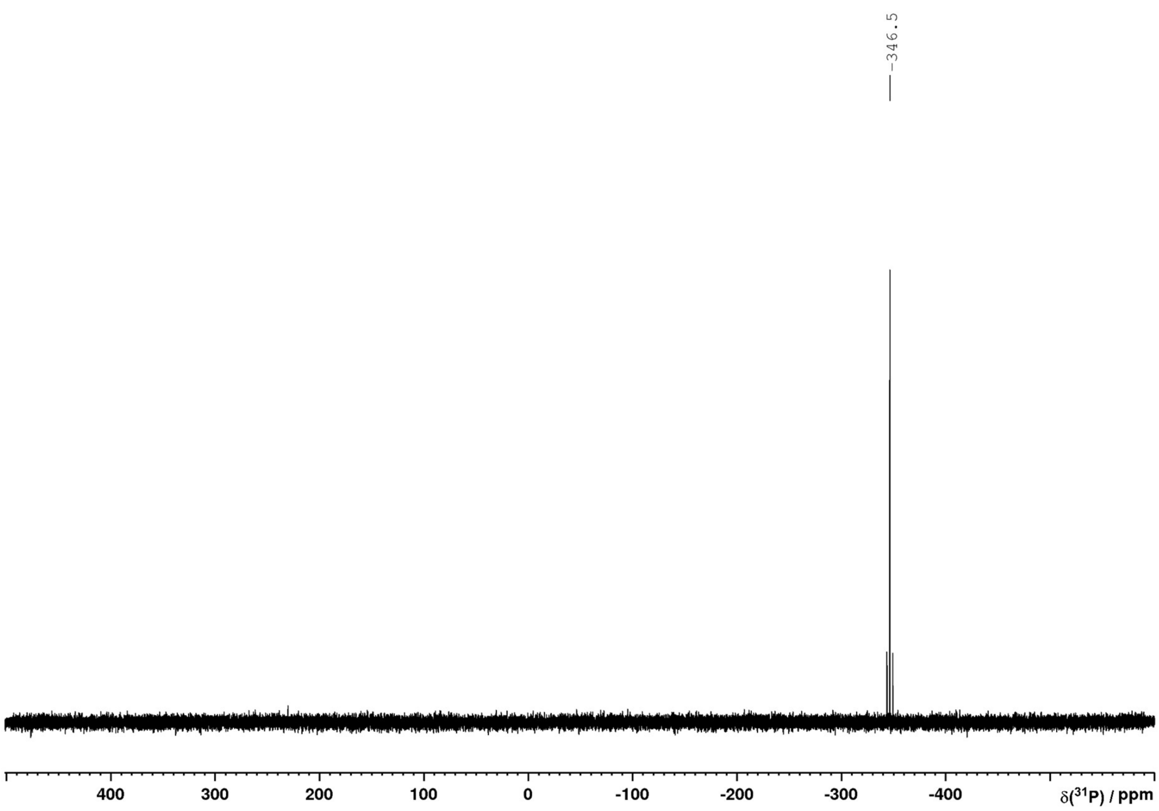

Figure S18. ^{31}P NMR spectrum of $(Bu_3Sn)_3P$ (**4**) in C_6D_6.

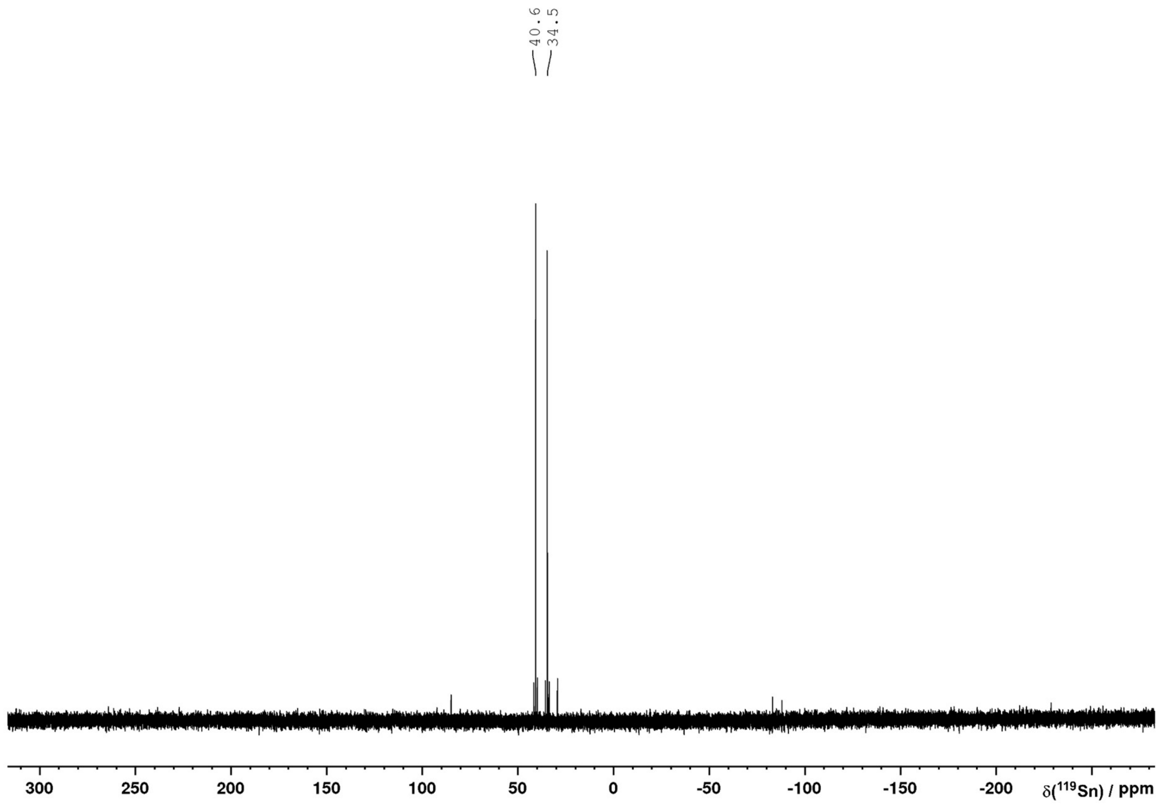

Figure S19. $^{119}Sn\{^1H\}$ NMR spectrum of $(Bu_3Sn)_3P$ (**4**) in C_6D_6.

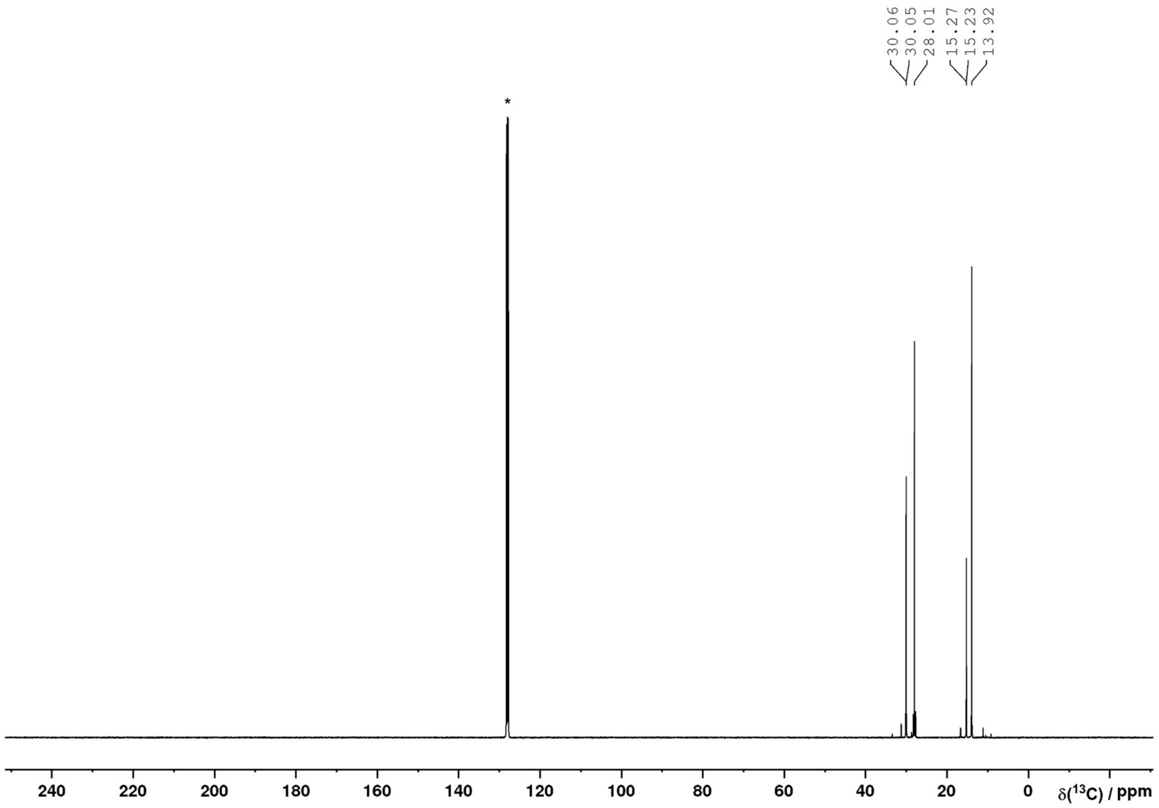

Figure S20. $^{13}C\{^{1}H\}$ NMR spectrum of $(Bu_3Sn)_3P$ (**4**) in C_6D_6 (*).

3. Synthesis and Isolation of Products Derived from $(Bu_3Sn)_3P$

3.1. Synthesis and isolation of THPC (5) *via* stannylation of P_{red} using Bu_3SnH and Bu_3SnOMe with recovery of Bu_3SnCl (6)

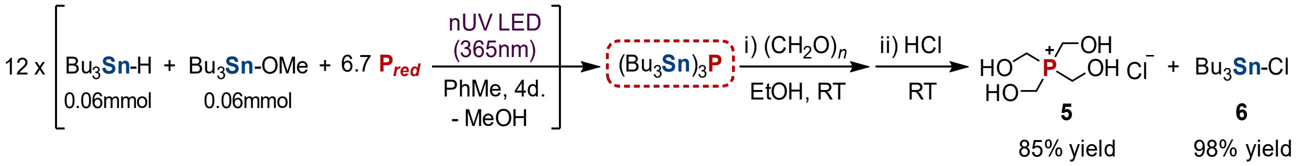

To provide sufficient material for reliable yield determination, a total of twelve reactions were performed in parallel using the following procedure: To a 10 mL, flat-bottomed, stoppered tube were added P_{red} (0.4 mmol, 12.4 mg), PhMe (50 µL), Bu_3SnH (16.1 µL, 0.06 mmol) and Bu_3SnOMe (17.3 µL, 0.06 mmol). The tube was sealed, placed in a water-cooled block to maintain near-ambient temperature, and irradiated with UV light (365 nm, 4.3 V, 700 mA, Osram OSLON SSL 80) for 4 days. The twelve reactions were then combined in a 100 mL Schlenk using PhMe (3 x 0.5 mL) to transfer and wash each tube. The combined red suspension was filtered and the remaining solid extracted with additional PhMe (2 x 10 mL), followed by removal of volatiles under vacuum. EtOH (10 mL) and paraformaldehyde (180.2 mg, 6.0 mmol) were added to the oily residue, and the resulting suspension was stirred at room temperature for 16 h. The mixture was frozen in a liquid-nitrogen bath, and HCl (4.0 M in 1,4-dioxane, 1.2 mL, 4.8 mmol) was added. After thawing, the reaction mixture

was stirred at room temperature for 2 h. Volatiles from the resulting yellowish suspension were removed under vacuum. The remaining oily solid residue was triturated with Et_2O (10 mL) overnight, filtered and washed with further Et_2O (2 x 5 mL). The resulting white solid was then again dissolved in EtOH (10 mL). Following filtration and removal of volatiles under vacuum, the desired product (**5**) was obtained as a white solid (78.1 mg, 85%).

The combined Et_2O washes from the above reaction were dried under vacuum to afford Bu_3SnCl (**6**) as a pale yellow oil (461.4 mg, 98%).

NMR data are identical to those given in section 1.3.

3.2. Synthesis and isolation of THP (9)

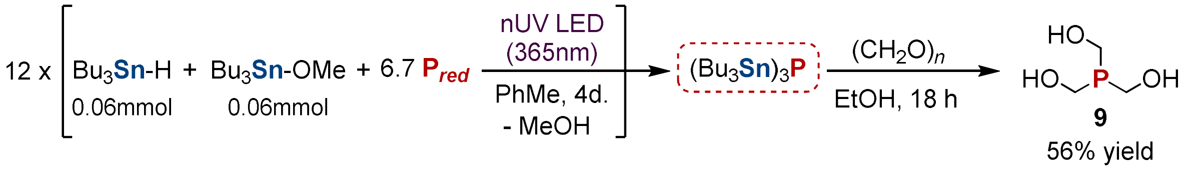

To provide sufficient material for reliable yield determination, a total of twelve reactions were performed in parallel using the following procedure: To a 10 mL, flat-bottomed, stoppered tube were added P_{red} (0.4 mmol, 12.4 mg), PhMe (50 µL), Bu_3SnH (16.1 µL, 0.06 mmol) and Bu_3SnOMe (17.3 µL, 0.06 mmol). The tube was sealed, placed in a water-cooled block to maintain near-ambient temperature, and irradiated with UV light (365 nm, 4.3 V, 700 mA, Osram OSLON SSL 80) for 4 days. The twelve reactions were then combined in a 100 mL Schlenk using PhMe (3 x 0.5 mL) to transfer and wash each tube. The combined red suspension was filtered and the remaining solid extracted with additional PhMe (2 x 10 mL), followed by removal of volatiles under vacuum. EtOH (10 mL) and paraformaldehyde (180.2 mg, 6.0 mmol) were added to the oily residue, and the resulting suspension was stirred at room temperature for 16 h. Volatiles were removed under vacuum, and the oily residue was disolved in PhMe (10 mL). Following filtration, degassed H_2O (10 mL) was added to the solution. The biphasic mixture was thoroughly stirred for 30 min, the toluene phase was removed, and the aqueous phase was washed with further PhMe (2 x 10 mL). Following removal of volatiles from the aqueous phase under vacuum, THP (**9**) was obtained as a colourless oil (33.4 mg, 56%).

^{1}H NMR (400 MHz, 300 K, D_2O) : δ = 3.99 ppm (d, $^{2}J(^{31}P\text{-}^{1}H)$ = 5.2 Hz).

$^{31}P\{^{1}H\}$ NMR (121 MHz, 300 K, D_2O) : δ = –23.6 ppm (s).

^{31}P NMR (121 MHz, 300 K, D_2O) : δ = –23.6 ppm (s).

$^{13}C\{^{1}H\}$ NMR (101 MHz, 300 K, D_2O): δ = 56.4 ppm (d, $^{1}J(^{31}P\text{-}^{13}C)$ = 8.5 Hz).

NMR data are consistent with our previous report.[4]

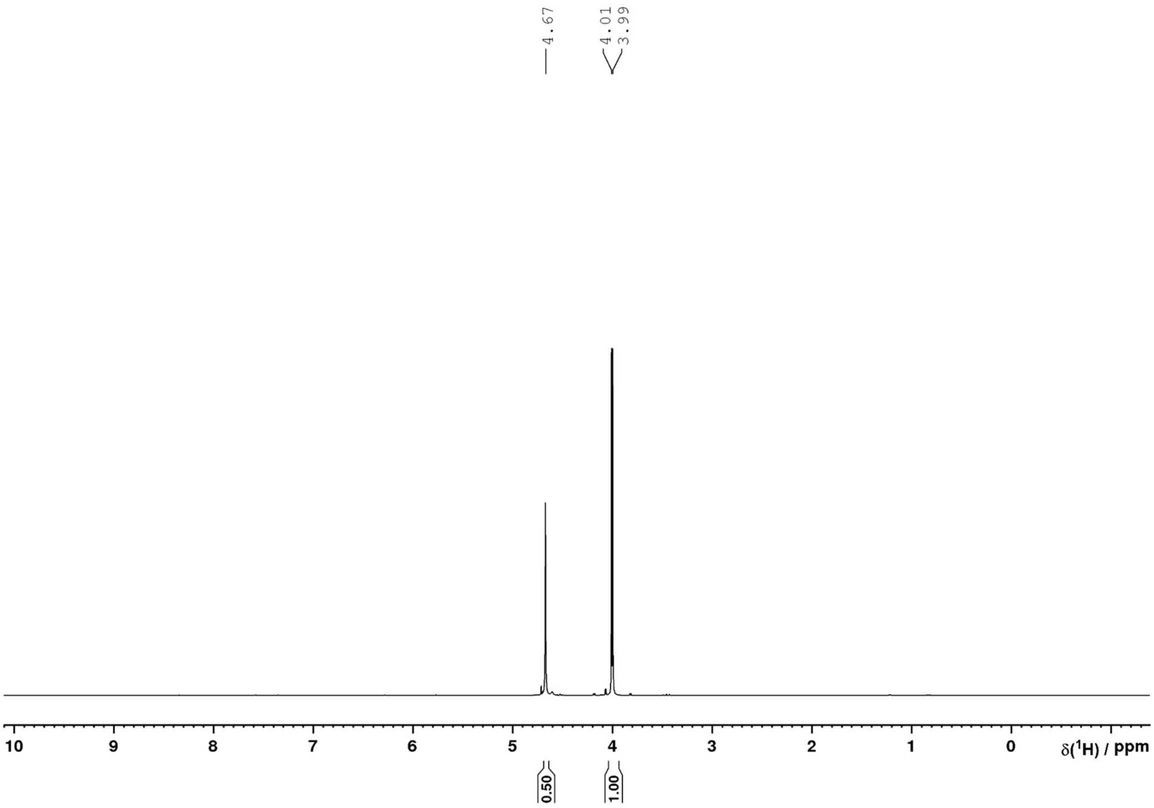

Figure S21. ^{1}H NMR spectrum of THP (**9**) in D_2O.

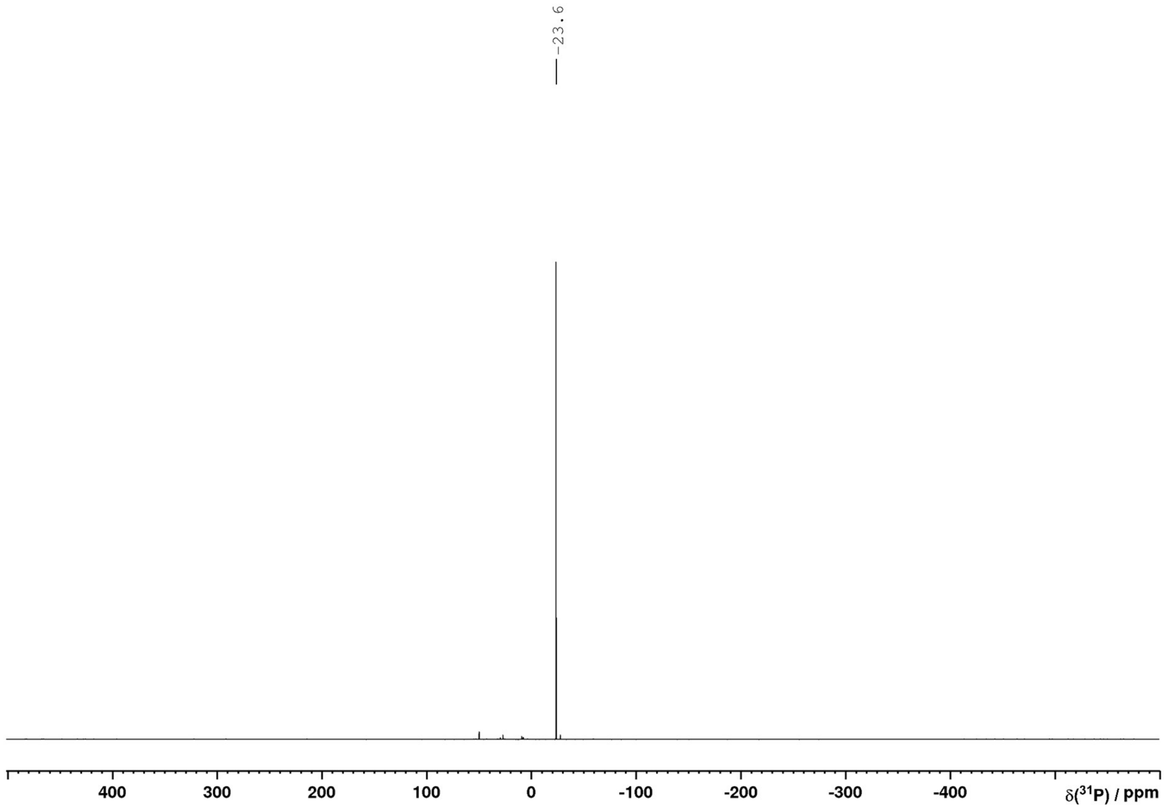

Figure S22. $^{31}P\{^{1}H\}$ NMR spectrum of THP (**9**) in D_2O.

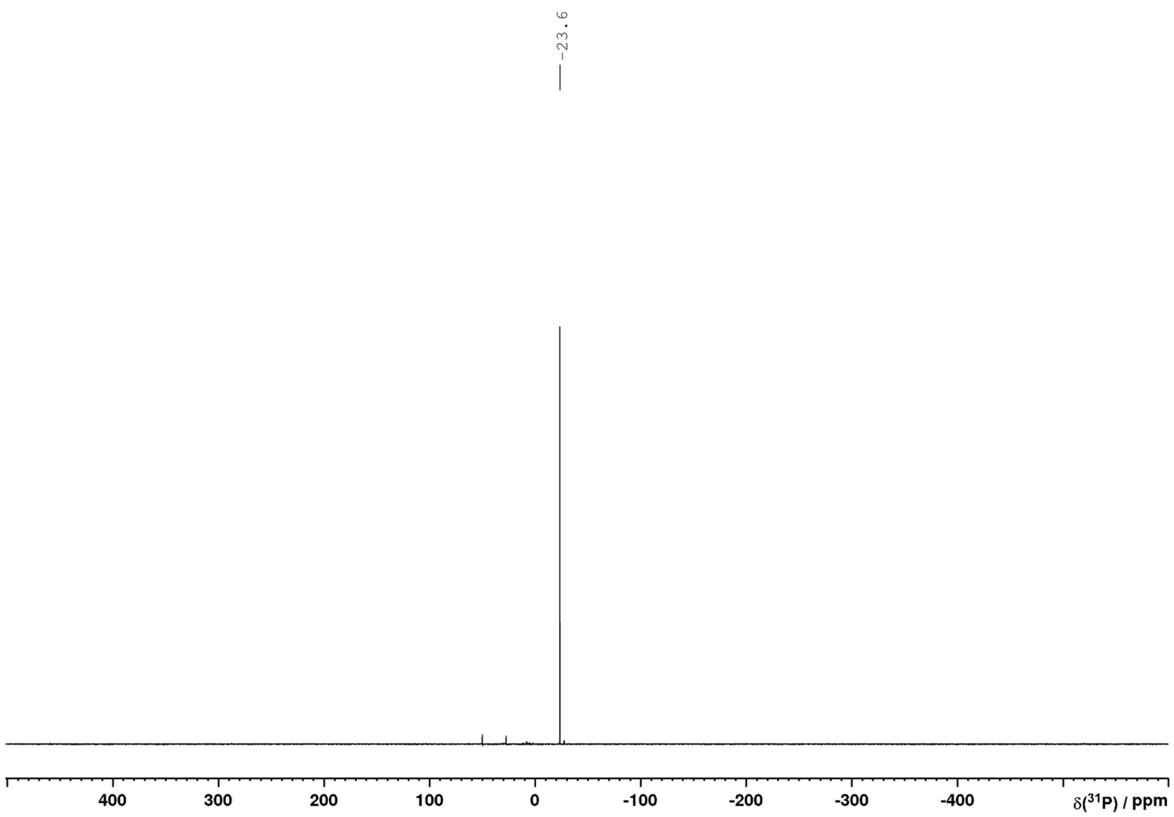

Figure S23. ^{31}P NMR spectrum of THP (**9**) in D_2O.

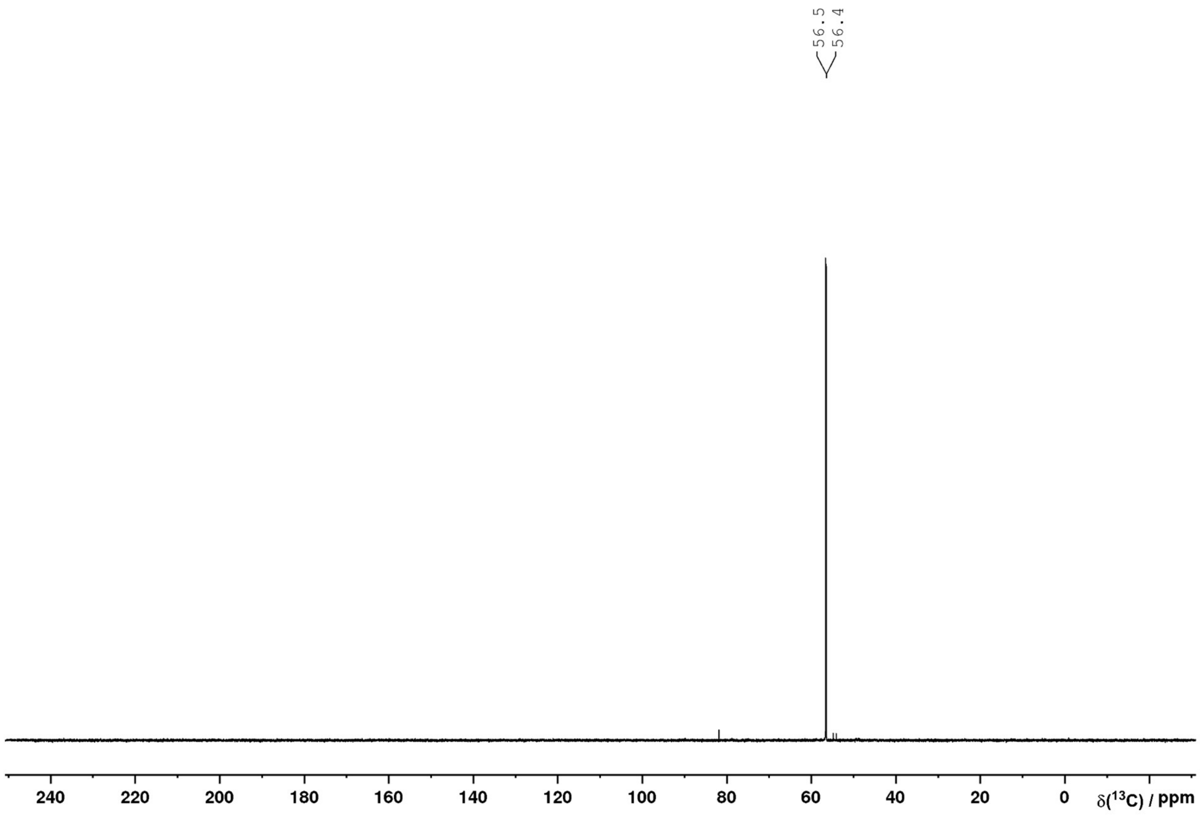

Figure S24. $^{13}C\{^1H\}$ NMR spectrum of THP (**9**) in D_2O.

3.3. Synthesis and isolation of $(HOCH_2)_3PO$ (10)

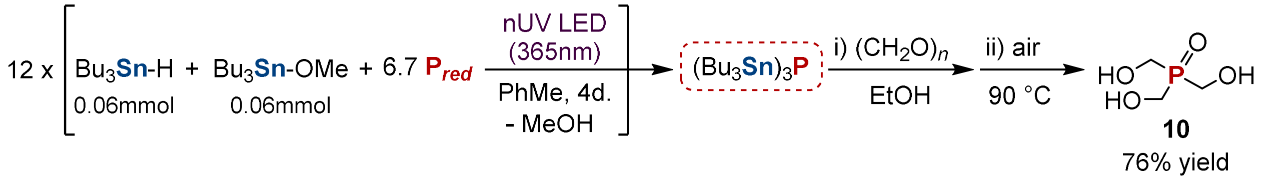

To provide sufficient material for reliable yield determination, a total of twelve reactions were performed in parallel using the following procedure: To a 10 mL, flat-bottomed, stoppered tube were added P_{red} (0.4 mmol, 12.4 mg), PhMe (50 µL), Bu_3SnH (16.1 µL, 0.06 mmol) and Bu_3SnOMe (17.3 µL, 0.06 mmol). The tube was sealed, placed in a water-cooled block to maintain near-ambient temperature, and irradiated with UV light (365 nm, 4.3 V, 700 mA, Osram OSLON SSL 80) for 4 days. The twelve reactions were then combined in a 100 mL Schlenk using PhMe (3 x 0.5 mL) to transfer and wash each tube. The combined red suspension was filtered and the remaining solid extracted with additional PhMe (2 x 10 mL), followed by removal of volatiles under vacuum. EtOH (10 mL) and paraformaldehyde (180.2 mg, 6.0 mmol) were added to the oily residue, and the resulting suspension was stirred at room temperature for 16 h. The resulting suspension was filtered and volatiles were removed under vacuum. Additional work-up was performed under air. To the oily residue thus obtained was added PhMe (10 mL) and H_2O (10 mL). The biphasic mixture was thoroughly stirred for 30 min and the aqueous phase was separated and washed with additional PhMe (3 x 10 mL), before being heated to 90 °C for 18 h while being kept open to air. Subsequent removal of volatiles yielded $(HOCH_2)_3PO$ (**10**) as a colourless oil (51.1 mg, 76%).

^1H NMR (400 MHz, 300 K, D_2O) : δ = 4.04 ppm (d, $^2J(^{31}P\text{-}^1H)$ = 3.0 Hz).

$^{31}P\{^1H\}$ NMR (121 MHz, 300 K, D_2O) : δ = 49.7 ppm (s).

^{31}P NMR (121 MHz, 300 K, D_2O) : δ = 49.7 ppm (s).

$^{13}C\{^1H\}$ NMR (101 MHz, 300 K, D_2O): δ = 54.3 ppm (d, $^1J(^{31}P\text{-}^{13}C)$ = 75.6 Hz).

NMR data are consistent with our previous report.[4]

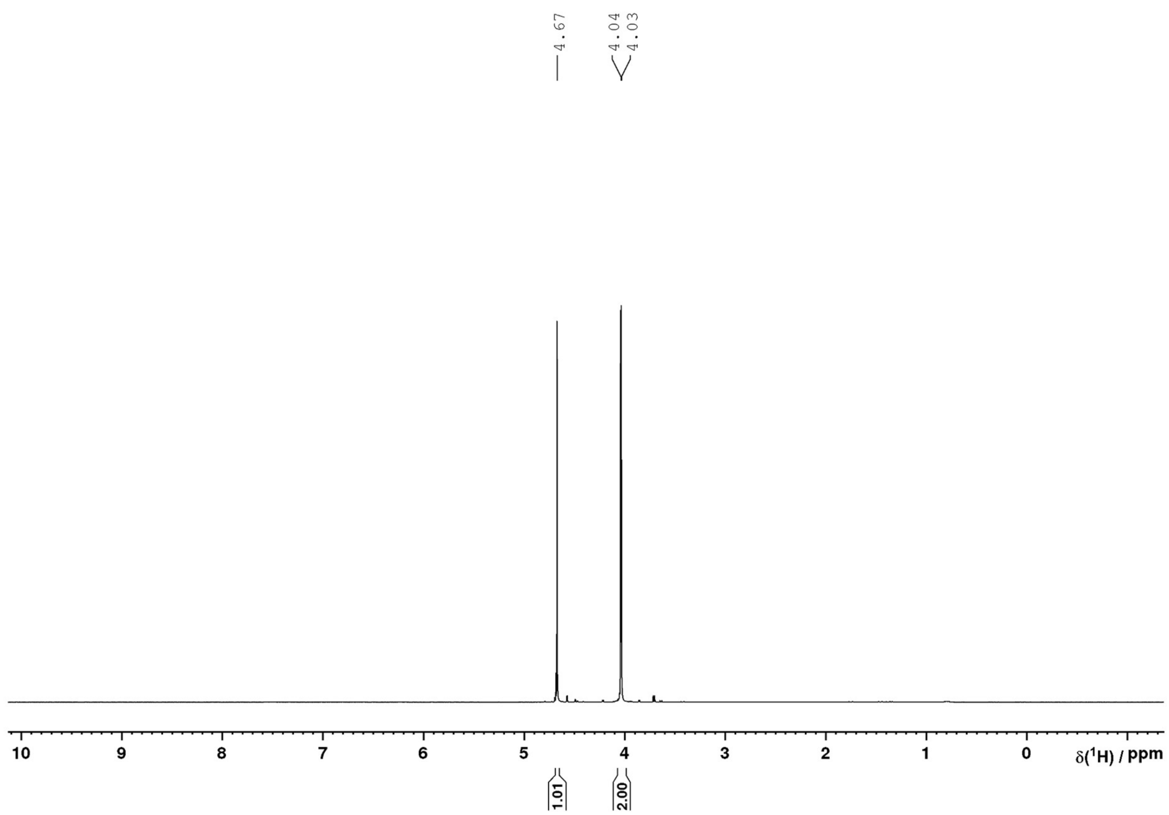

Figure S25. ^{1}H NMR spectrum of THPO (**10**) in D_2O.

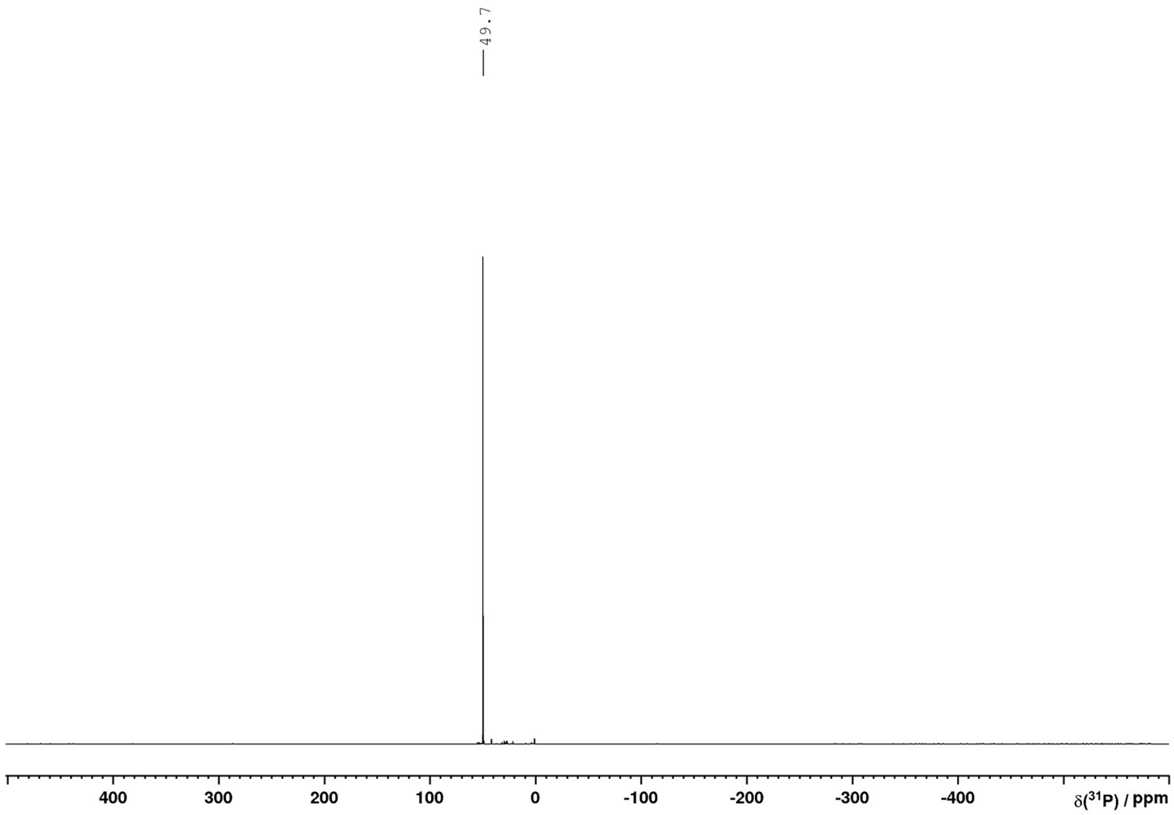

Figure S26. $^{31}P\{^{1}H\}$ NMR spectrum of THPO (**10**) in D_2O.

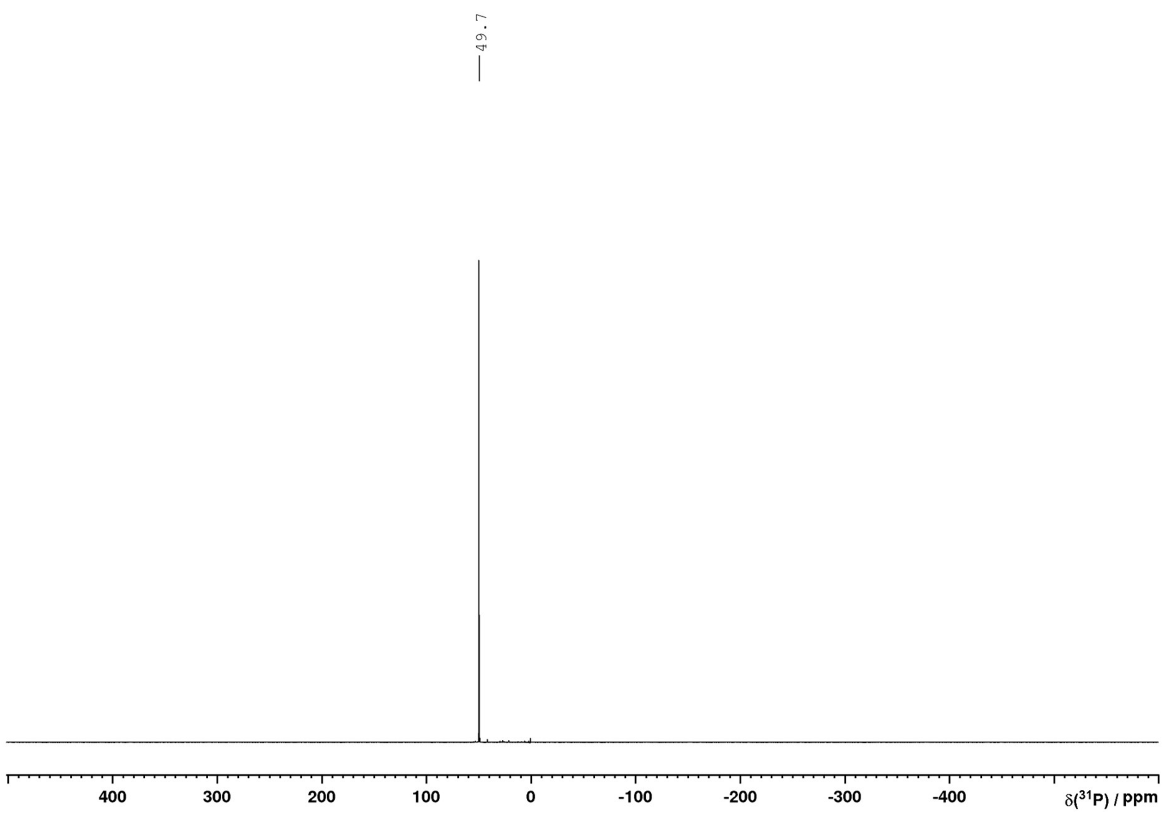

Figure S27. ^{31}P NMR spectrum of THPO (**10**) in D_2O.

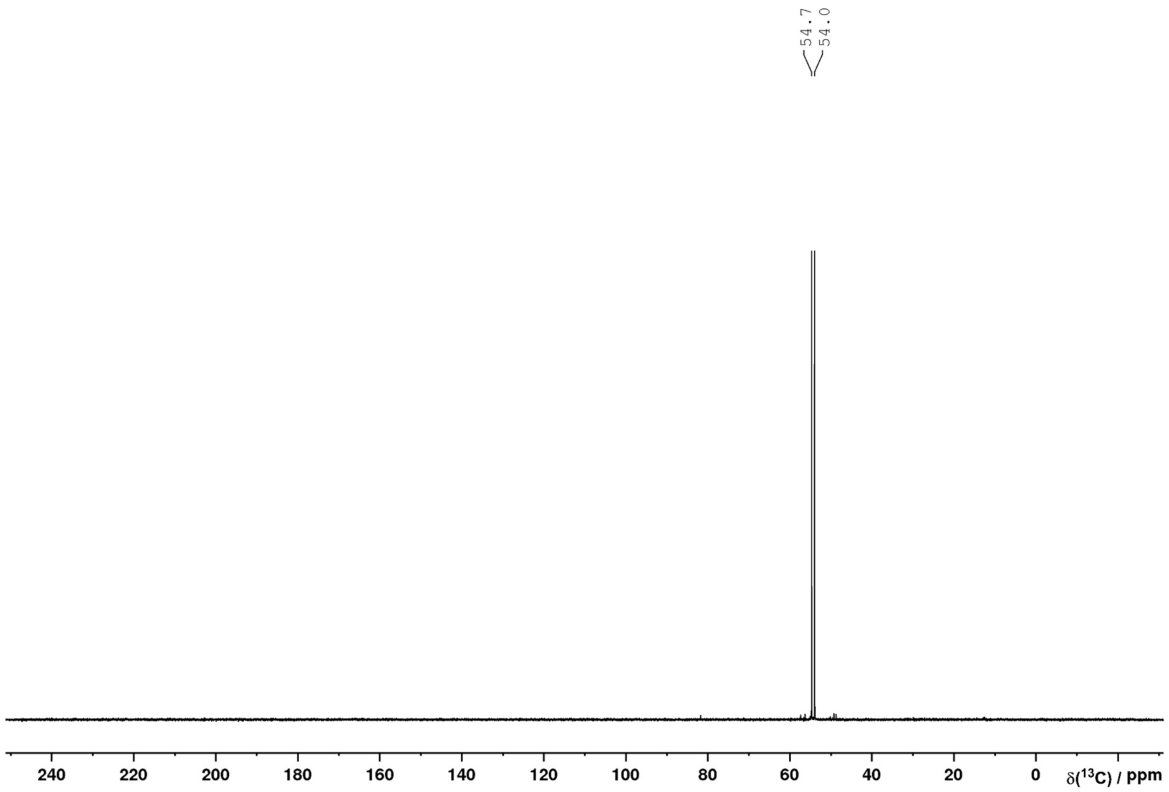

Figure S28. $^{13}C\{^1H\}$ NMR spectrum of THPO (**10**) in D_2O.

3.4. Synthesis and isolation of [Bn$_4$P]Br (7a)

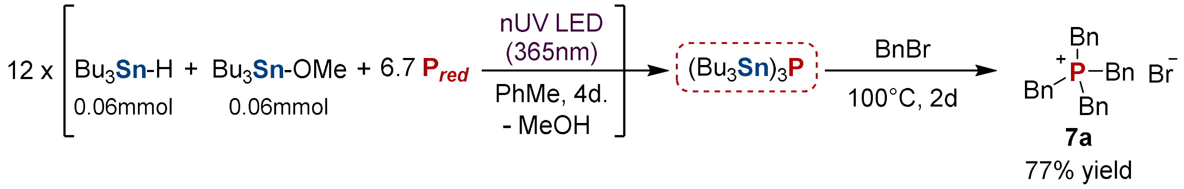

To provide sufficient material for reliable yield determination, a total of twelve reactions were performed in parallel using the following procedure: To a 10 mL, flat-bottomed, stoppered tube were added P$_{red}$ (0.4 mmol, 12.4 mg), PhMe (50 µL), Bu$_3$SnH (16.1 µL, 0.06 mmol) and Bu$_3$SnOMe (17.3 µL, 0.06 mmol). The tube was sealed, placed in a water-cooled block to maintain near-ambient temperature, and irradiated with UV light (365 nm, 4.3 V, 700 mA, Osram OSLON SSL 80) for 4 days. The twelve reactions were then combined in a 100 mL Schlenk using PhMe (3 x 0.5 mL) to transfer and wash each tube. The combined red suspension was filtered and the remaining solid extracted with additional PhMe (2 x 10 mL), followed by removal of volatiles under vacuum. PhMe (10 mL) and benzyl bromide (571 µL, 4.8 mmol) were added to the oily residue, and heated to 100 °C with stirring for 2 days. After cooling to room temperature, volatiles from the reaction mixture were removed under vacuum. The remaining oily solid residue was triturated with hexane (10 mL) overnight, to give TBPB (**7a**) as a white solid (176.1 mg, 77%) after filtration, washing with additional hexane (2 x 10 mL) and drying under vacuum.

^{1}H NMR (400 MHz, 300 K, CD$_3$CN) : δ = 7.36 ppm (3H, m), 7.15 ppm (2H, m), 3.73 ppm (2H, d, $^2J(^{31}P-^1H)$ = 14.3 Hz).

^{31}P{^{1}H} NMR (121 MHz, 300 K, CD$_3$CN) : δ = 25.7 ppm (s).

^{31}P NMR (121 MHz, 300 K, CD$_3$CN) : δ = 25.7 ppm (m).

^{13}C{^{1}H} NMR (101 MHz, 300 K, C$_6$D$_6$): δ = 131.6 (d, $J(^{31}P-^1H)$ = 5.3 Hz), 130.2 (d, $J(^{31}P-^1H)$ = 2.9 Hz), 129.3 (d, $J(^{31}P-^1H)$ = 3.4 Hz), 128.8 (d, $J(^{31}P-^1H)$ = 8.1 Hz), 27.4 ppm (d, $J(^{31}P-^1H)$ = 43.2 Hz).

NMR data are consistent with our previous report.[4]

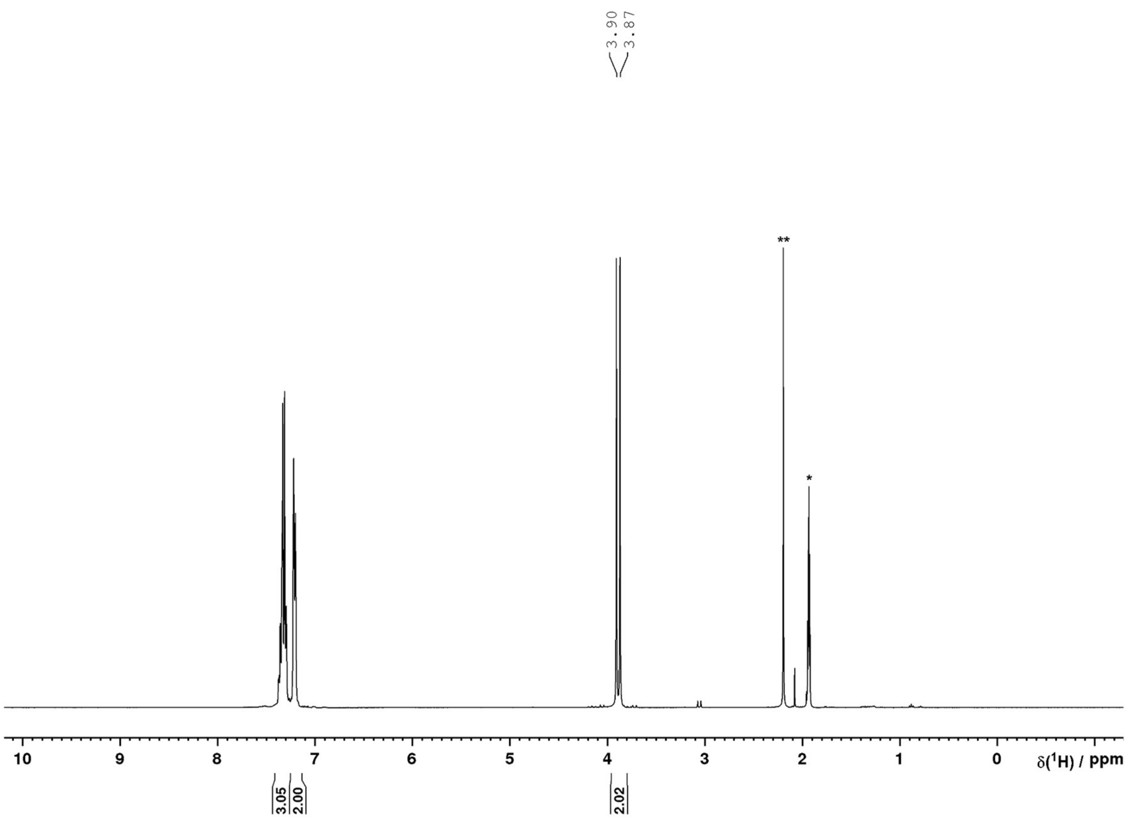

Figure S29. ^{1}H NMR spectrum of $[Bn_4P]Br$ (**7a**) in CD_3CN (*solvent, **H_2O).

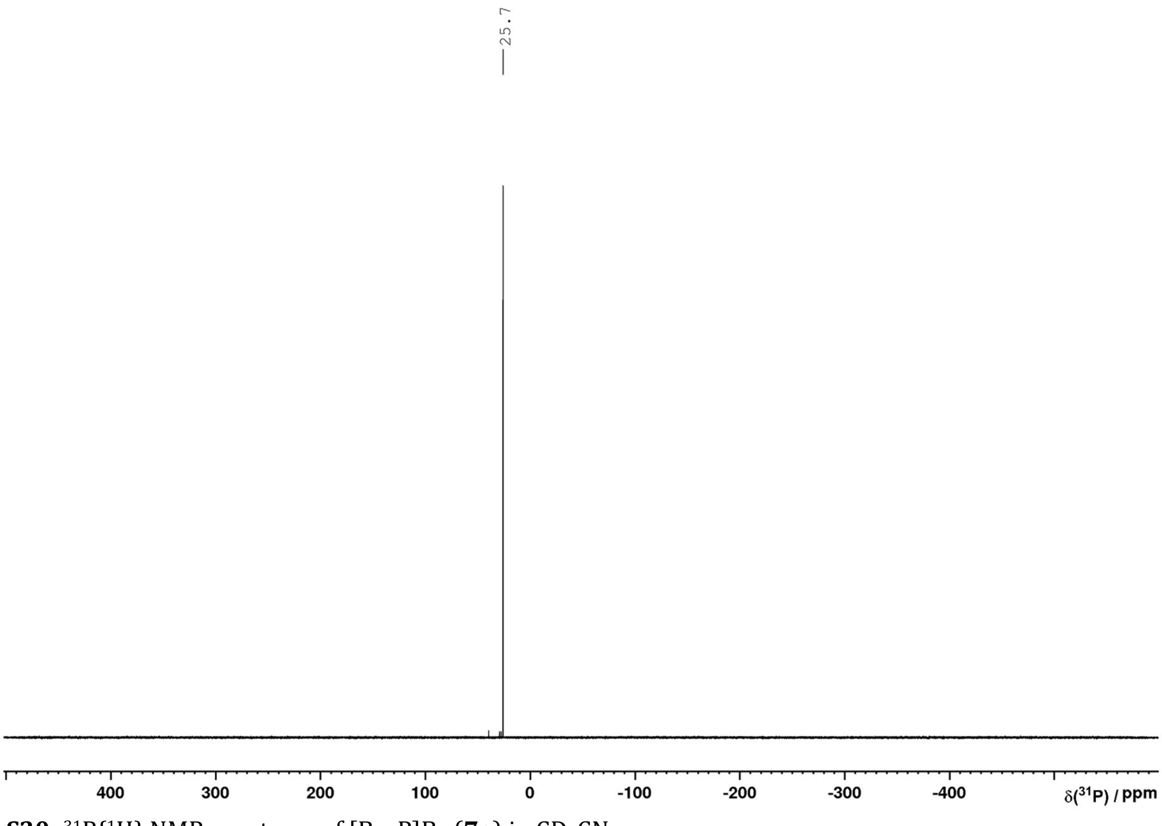

Figure S30. $^{31}P\{^1H\}$ NMR spectrum of $[Bn_4P]Br$ (**7a**) in CD_3CN.

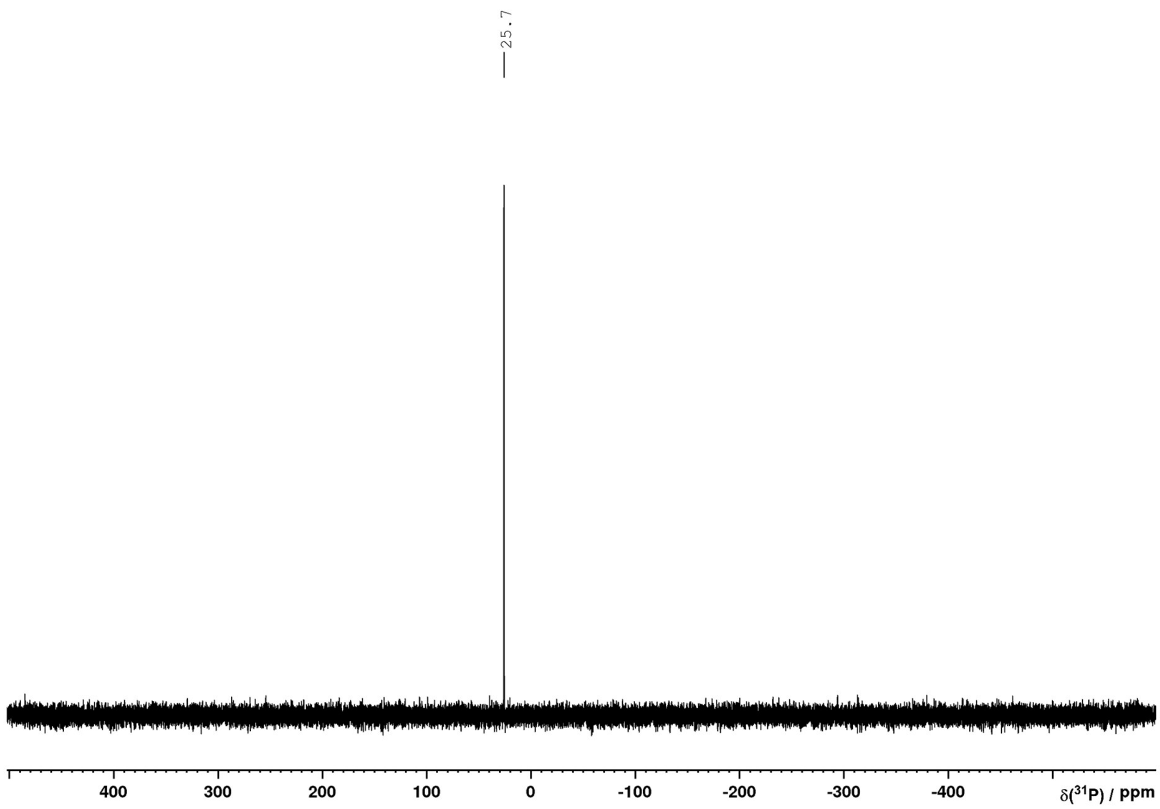

Figure S31. ^{31}P NMR spectrum of $[Bn_4P]Br$ (**7a**) in CD_3CN.

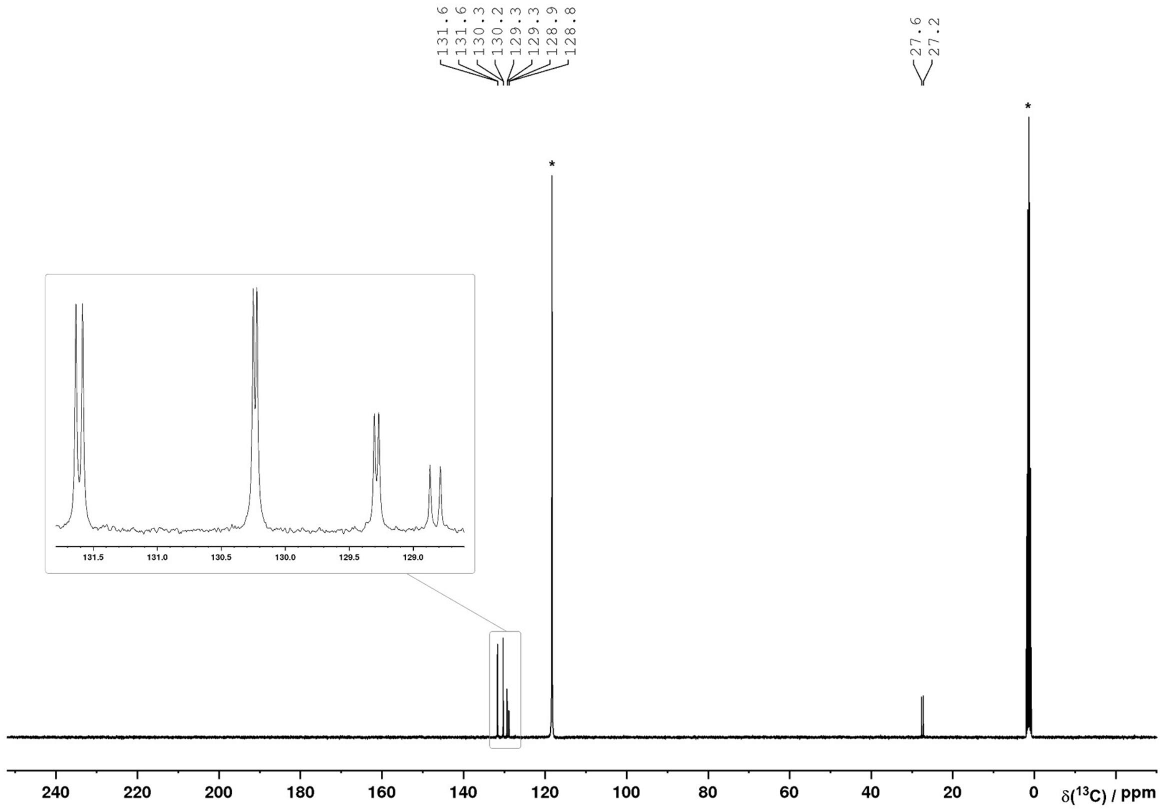

Figure S32. $^{13}C\{^1H\}$ NMR spectrum of $[Bn_4P]Br$ (**7a**) in CD_3CN (*).

3.5. Synthesis and isolation of [Et$_4$P]Br (7b)

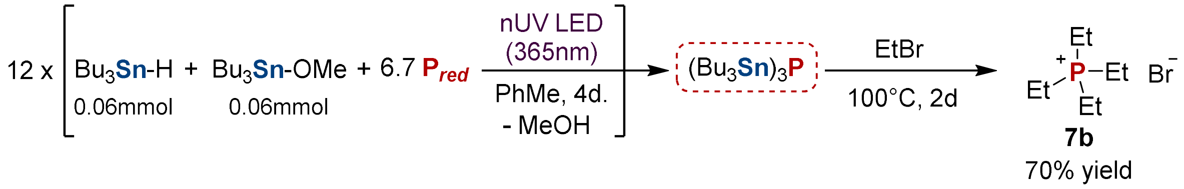

To provide sufficient material for reliable yield determination, a total of twelve reactions were performed in parallel using the following procedure: To a 10 mL, flat-bottomed, stoppered tube were added P_{red} (0.4 mmol, 12.4 mg), PhMe (50 µL), Bu_3SnH (16.1 µL, 0.06 mmol) and Bu_3SnOMe (17.3 µL, 0.06 mmol). The tube was sealed, placed in a water-cooled block to maintain near-ambient temperature, and irradiated with UV light (365 nm, 4.3 V, 700 mA, Osram OSLON SSL 80) for 4 days. The twelve reactions were then combined in a 100 mL Schlenk using PhMe (3 x 0.5 mL) to transfer and wash each tube. The combined red suspension was filtered and the remaining solid extracted with additional PhMe (2 x 10 mL), followed by removal of volatiles under vacuum. PhMe (10 mL) and ethyl bromide (358 µL, 4.8 mmol) were added to the oily residue, and heated to 100 °C with stirring for 2 days. After cooling to room temperature, volatiles from the reaction mixture were removed under vacuum. The remaining oily solid residue was triturated with hexane (10 mL) overnight, to give TEPB (**7b**) as a white solid (76.4 mg, 70%) after filtration, washing with additional hexane (2 x 10 mL) and drying under vacuum.

^{1}H NMR (400 MHz, 300 K, CD_3CN) : δ = 2.19 ppm (2H, dq, $^2J(^{31}P-^1H)$ = 13.0 Hz, $^3J(^1H-^1H)$ = 7.7 Hz), 1.19 ppm (3H, dt, $^3J(^{31}P-^1H)$ = 18.0 Hz, $^3J(^1H-^1H)$ = 7.7 Hz).

$^{31}P\{^1H\}$ NMR (121 MHz, 300 K, CD_3CN) : δ = 42.2 ppm (s).

^{31}P NMR (121 MHz, 300 K, CD_3CN) : δ = 42.2 ppm (m).

$^{13}C\{^1H\}$ NMR (101 MHz, 300 K, CD_3CN): δ = 11.7 (d, $^1J(^{31}P-^1H)$ = 49.5 Hz), 5.7 ppm (d, $^2J(^{31}P-^1H)$ = 5.4 Hz).

NMR data are consistent with our previous report.[4]

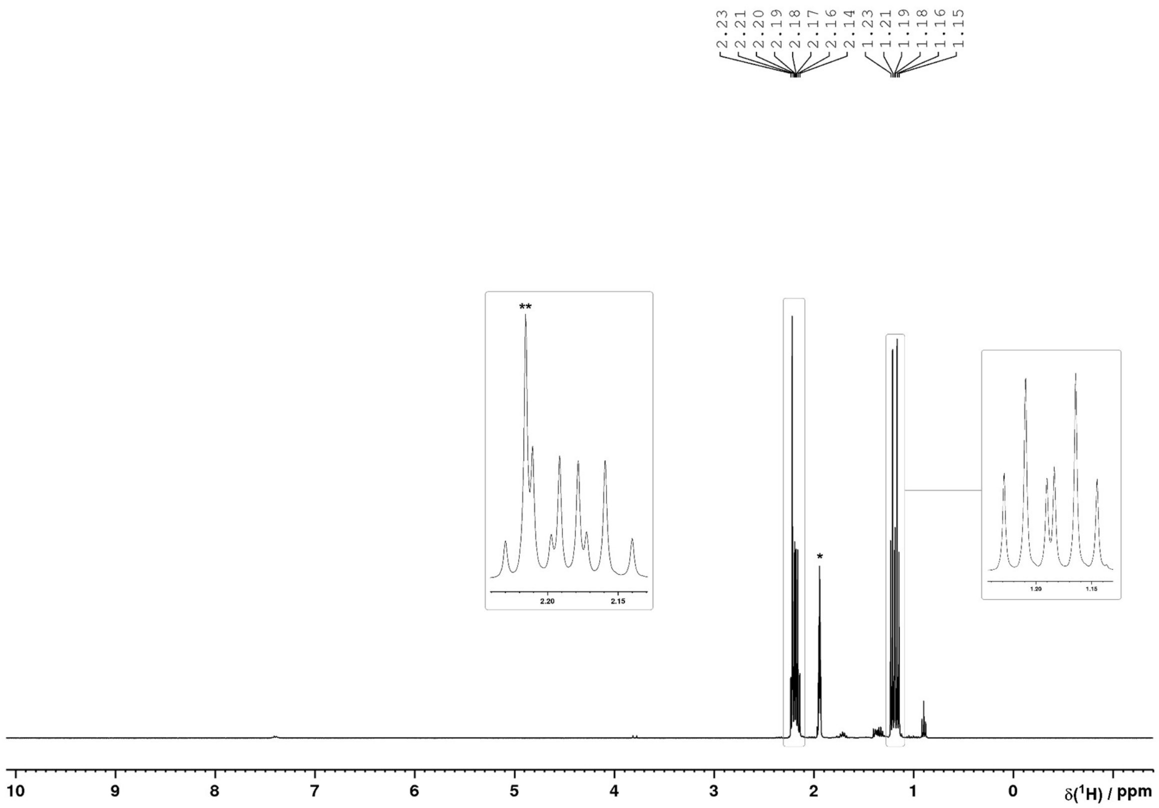

Figure S33. ^{1}H NMR spectrum of $[Et_4P]Br$ (**7b**) in CD_3CN (* solvent, **H_2O).

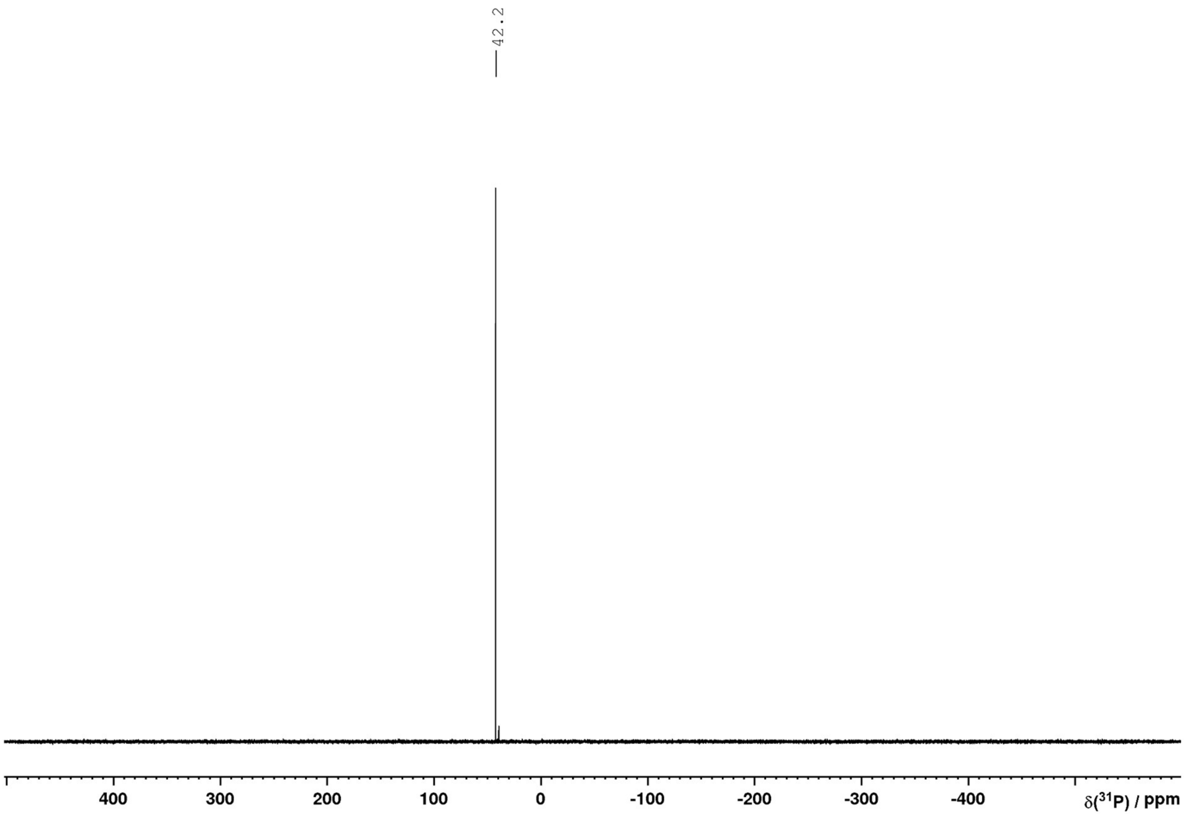

Figure S34. $^{31}P\{^{1}H\}$ NMR spectrum of $[Et_4P]Br$ (**7b**) in CD_3CN.

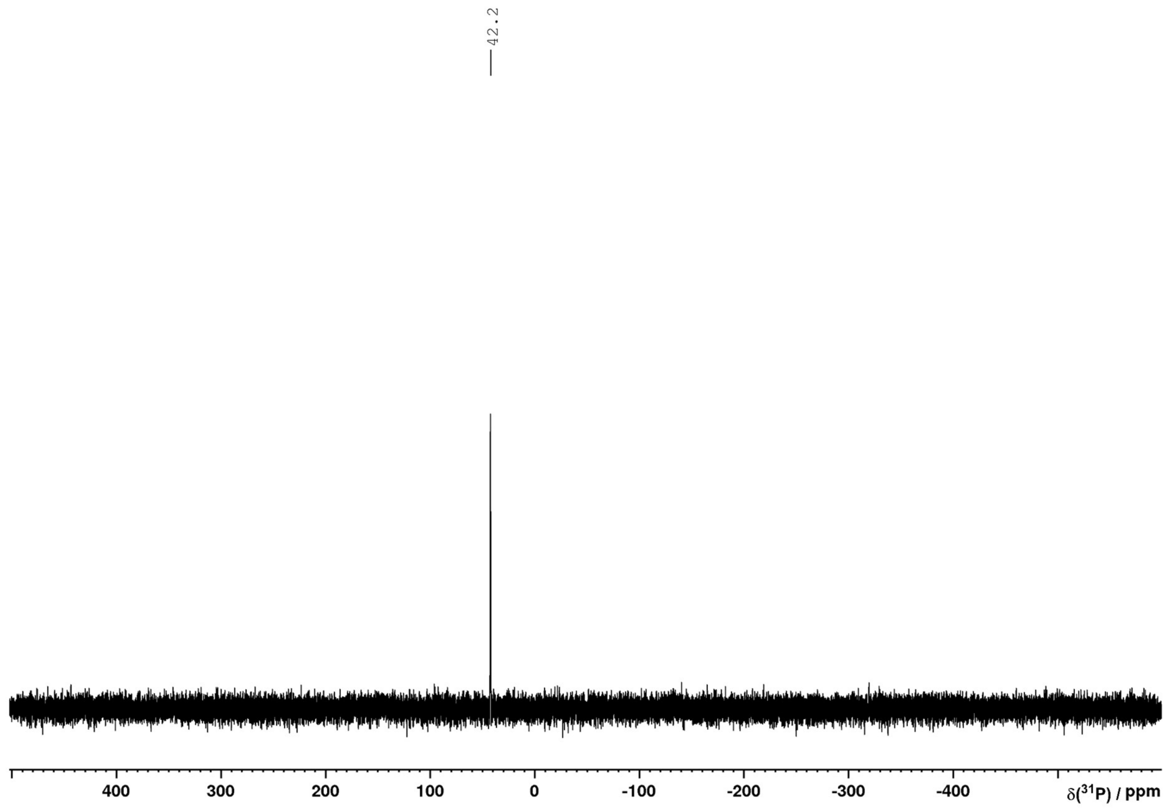

Figure S35. ^{31}P NMR spectrum of $[Et_4P]Br$ (**7b**) in CD_3CN.

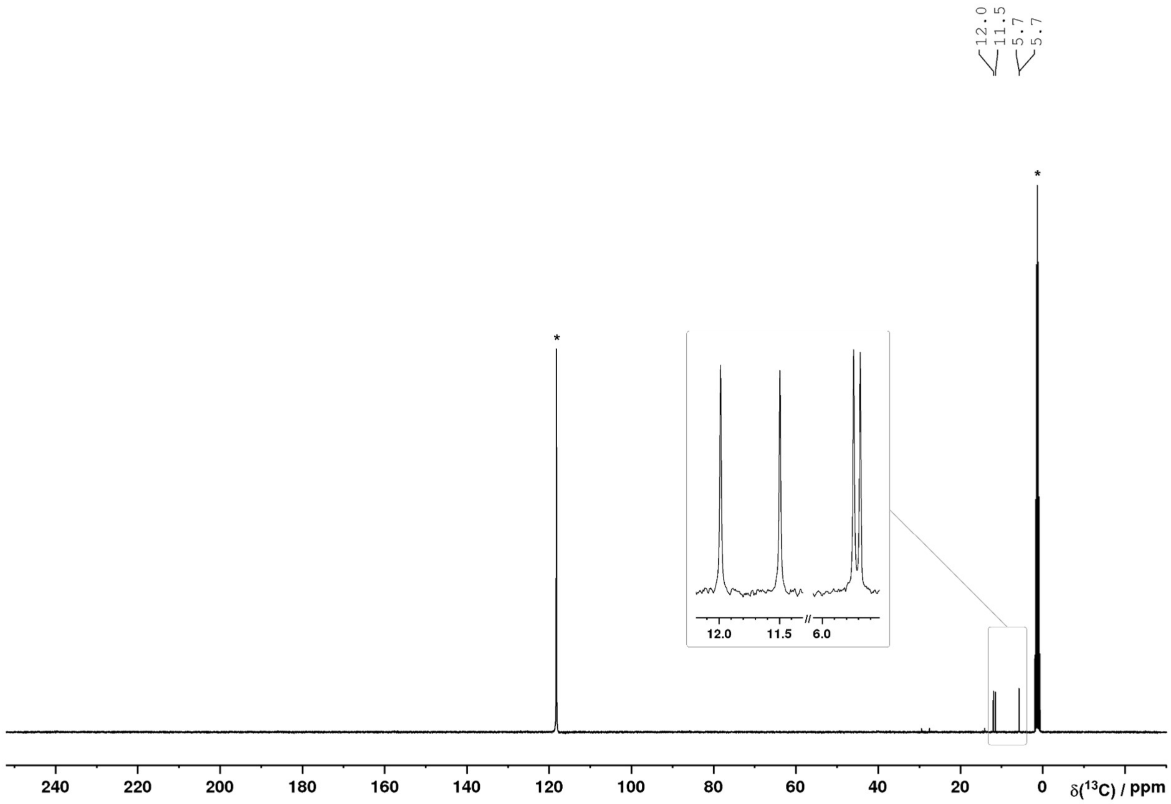

Figure S36. $^{13}C\{^1H\}$ NMR spectrum of $[Et_4P]Br$ (**7b**) in CD_3CN (*).

3.6. Synthesis and isolation of $P(C(O)Ph)_3$ (8a)

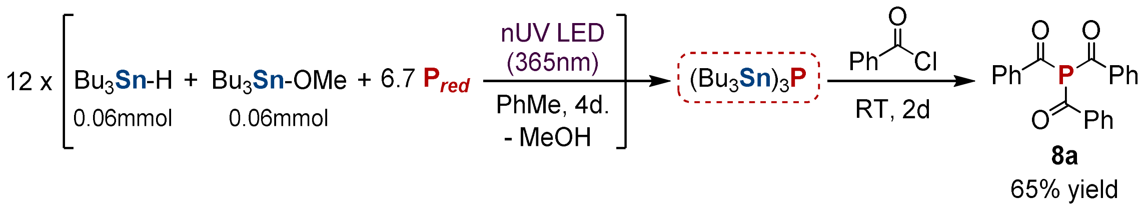

To provide sufficient material for reliable yield determination, a total of twelve reactions were performed in parallel using the following procedure: To a 10 mL, flat-bottomed, stoppered tube were added P_{red} (0.4 mmol, 12.4 mg), PhMe (50 µL), Bu_3SnH (16.1 µL, 0.06 mmol) and Bu_3SnOMe (17.3 µL, 0.06 mmol). The tube was sealed, placed in a water-cooled block to maintain near-ambient temperature, and irradiated with UV light (365 nm, 4.3 V, 700 mA, Osram OSLON SSL 80) for 4 days. The twelve reactions were then combined in a 100 mL Schlenk using PhMe (3 x 0.5 mL) to transfer and wash each tube. The combined red suspension was filtered and the remaining solid extracted with additional PhMe (2 x 10 mL), followed by removal of volatiles under vacuum. PhMe (10 mL) and PhC(O)Cl (223 µL, 1.92 mmol) were added to the oily residue, and the yellow mixture was stirred at room temperature for 2 days. Volatiles from the intense yellow mixture were removed under vacuum, and the remaining yellow solid was washed with *n*-hexane (3 x 10 mL). The remaining yellow residue was recrystallized from THF/*n*-hexane at –35 °C, to afford the desired product (**8a**) as yellow needles (108 mg, 65%).

^1H NMR (400 MHz, 300 K, C_6D_6): δ = 7.98 ppm (2H, m), 7.03 ppm (1H, tt, $^3J(^1H\text{-}^1H)$ =7.4 Hz, $^5J(^1H\text{-}^1H)$ =1.3 Hz), 6.96 ppm (2H, m).

$^{31}P\{^1H\}$ NMR (121 MHz, 300 K, C_6D_6): δ = 54.3 ppm (s).

^{31}P NMR (121 MHz, 300 K, C_6D_6): δ = 54.3 ppm (s).

$^{13}C\{^1H\}$ NMR (101 MHz, 300 K, C_6D_6): δ = 205.8 (d, $J(^{31}P\text{-}^1H)$ = 32.8 Hz), 140.9 (d, $J(^{31}P\text{-}^1H)$ = 35.2 Hz), 133.9 (d, $J(^{31}P\text{-}^1H)$ = 1.1 Hz), 129.0 (d, $J(^{31}P\text{-}^1H)$ = 8.0 Hz), 128.9 ppm (d, $J(^{31}P\text{-}^1H)$ = 0.7 Hz).

NMR data are consistent with our previous report.[4]

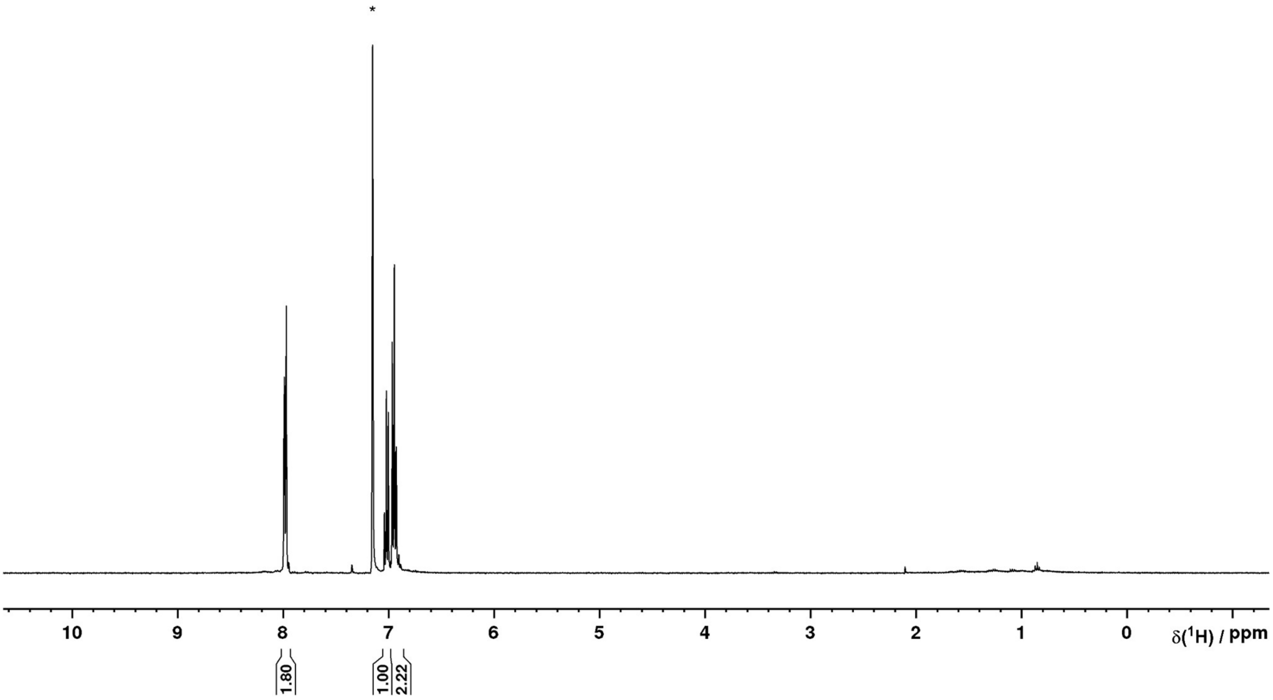

Figure S37. ^{1}H NMR spectrum of P(C(O)Ph)$_3$ (**8a**) in C_6D_6 (*).

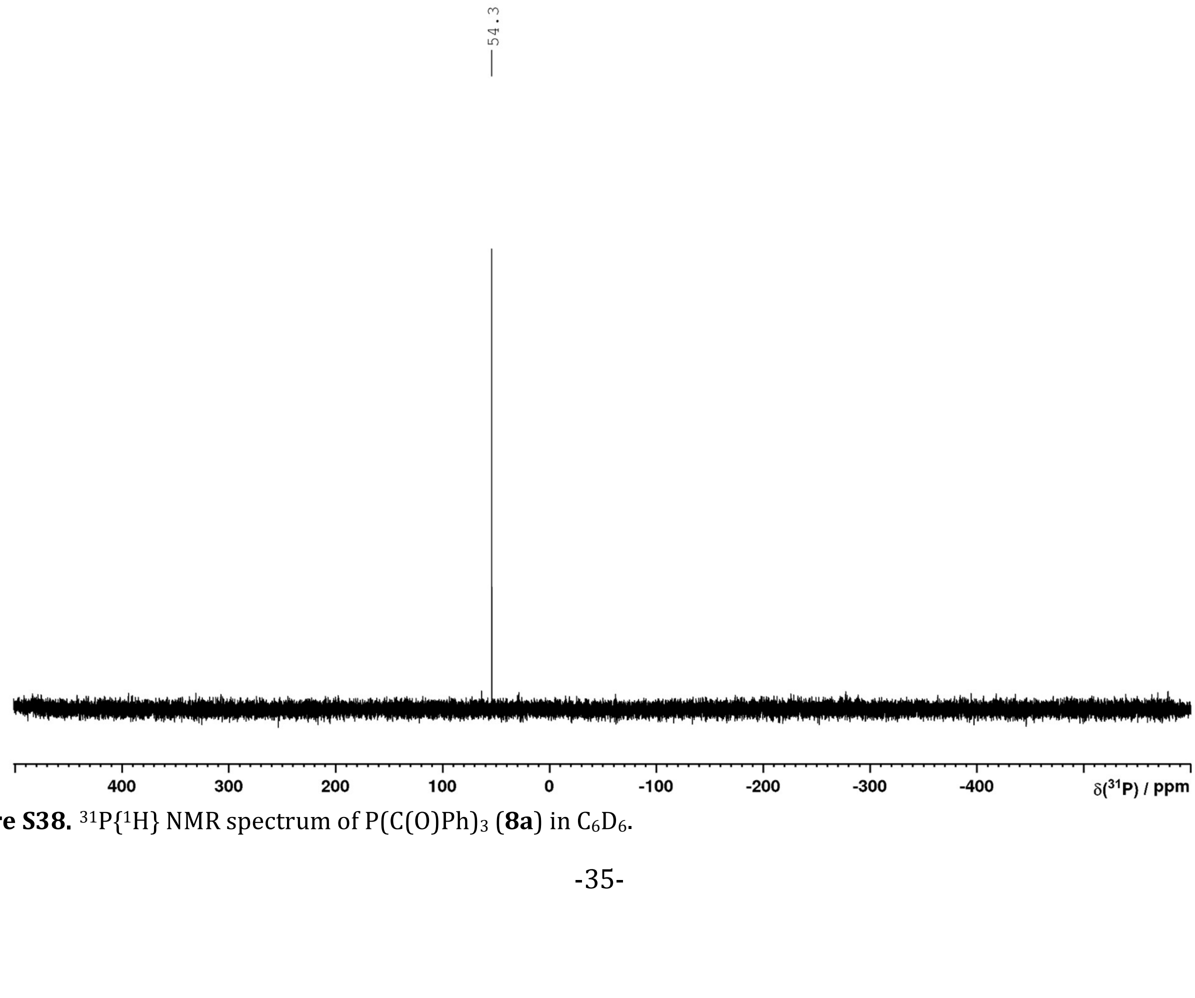

Figure S38. $^{31}P\{^{1}H\}$ NMR spectrum of P(C(O)Ph)$_3$ (**8a**) in C_6D_6.

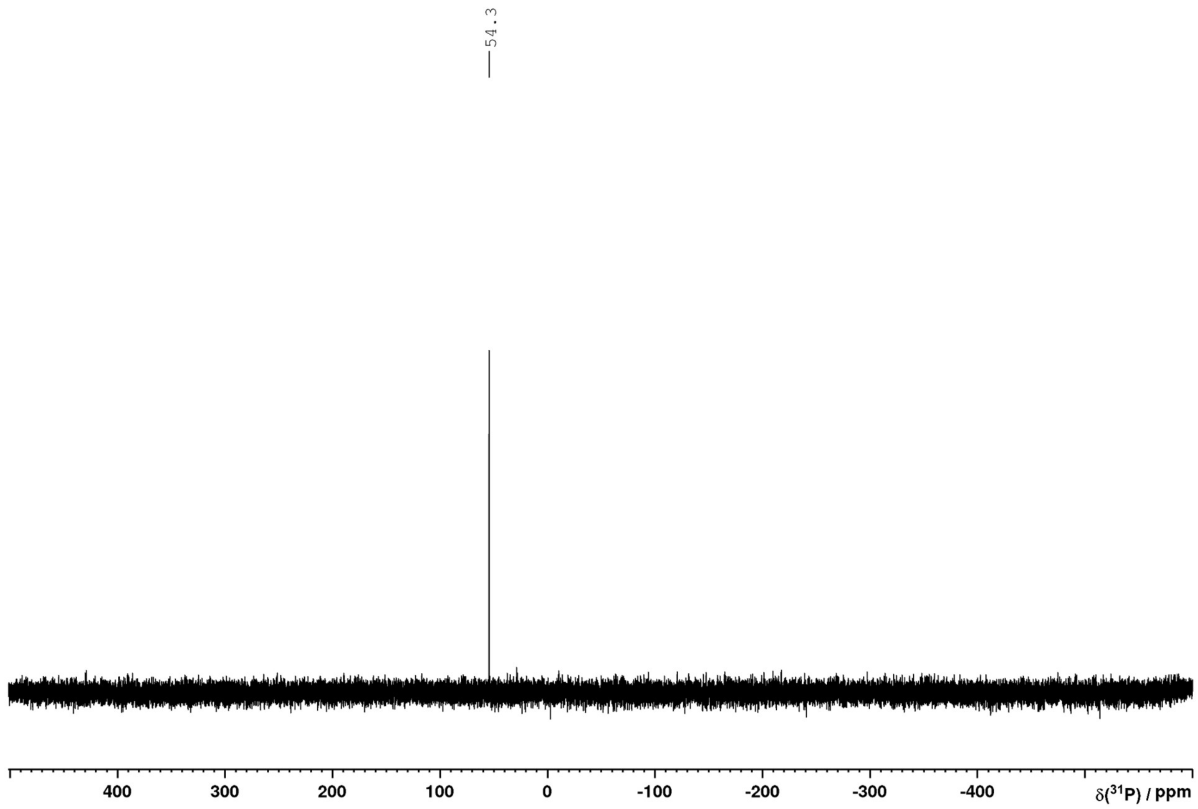

Figure S39. ^{31}P NMR spectrum of $P(C(O)Ph)_3$ (**8a**) in C_6D_6.

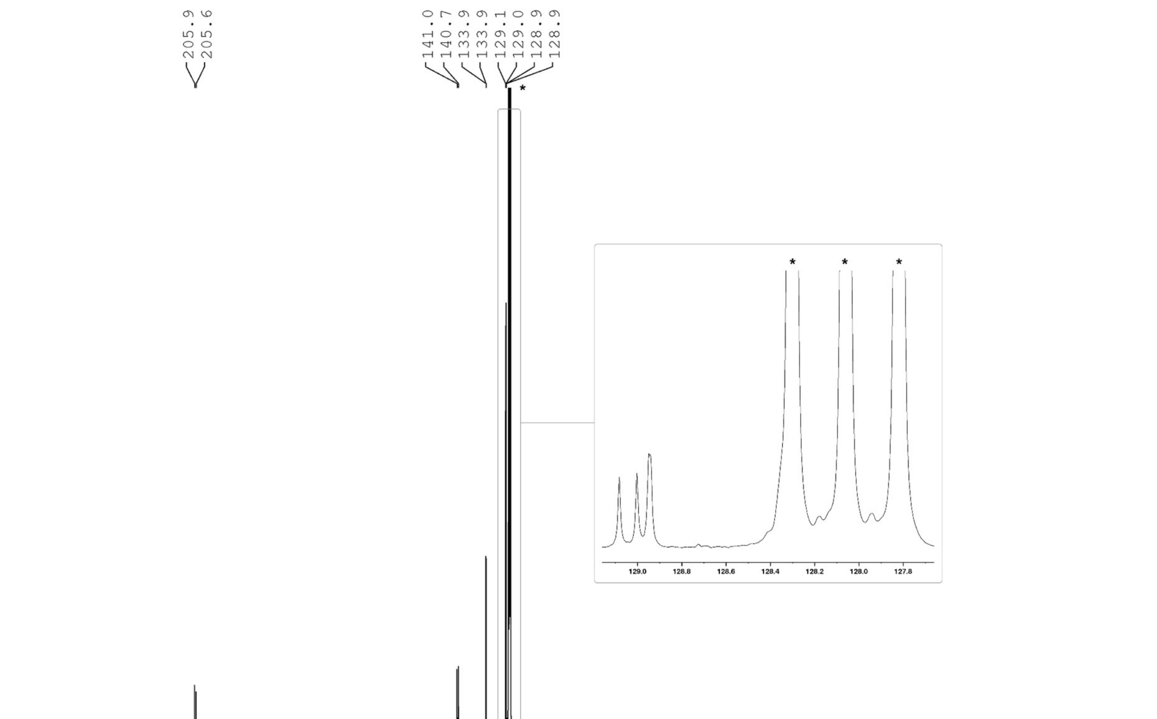

Figure S40. $^{13}C\{^1H\}$ NMR spectrum of $P(C(O)Ph)_3$ (**8a**) in C_6D_6. Solvent resonance (*) truncated for clarity.

3.7. Synthesis and isolation of P(C(O)*t*Bu)$_3$ (8b)

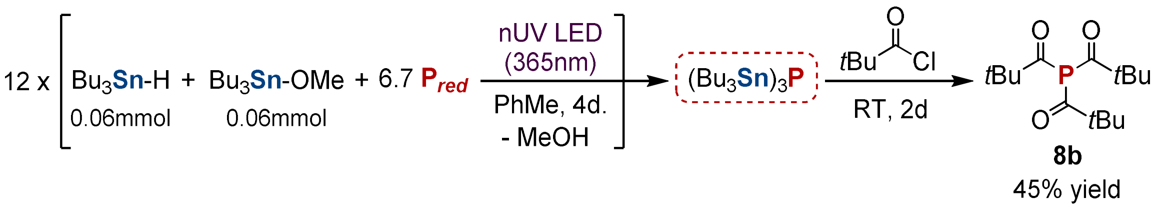

To provide sufficient material for reliable yield determination, a total of twelve reactions were performed in parallel using the following procedure: To a 10 mL, flat-bottomed, stoppered tube were added P_{red} (0.4 mmol, 12.4 mg), PhMe (50 µL), Bu_3SnH (16.1 µL, 0.06 mmol) and Bu_3SnOMe (17.3 µL, 0.06 mmol). The tube was sealed, placed in a water-cooled block to maintain near-ambient temperature, and irradiated with UV light (365 nm, 4.3 V, 700 mA, Osram OSLON SSL 80) for 4 days. The twelve reactions were then combined in a 100 mL Schlenk using PhMe (3 x 0.5 mL) to transfer and wash each tube. The combined red suspension was filtered and the remaining solid extracted with additional PhMe (2 x 10 mL), followed by removal of volatiles under vacuum. PhMe (10 mL) and *t*BuC(O)Cl (235 µL, 1.92 mmol) were added to the oily residue, and the yellowish mixture was stirred at room temperature for 1 day. Volatiles from the yellowish mixture were removed under vacuum, and the remaining yellow oily solid residue was recrystallized from *n*-hexane at −35 °C, to afford the desired product (**8b**) as colourless needles (62 mg, 45%).

^{1}H NMR (400 MHz, 300 K, C_6D_6): δ = 1.08 ppm (s).

^{31}P{^{1}H} NMR (121 MHz, 300 K, C_6D_6): δ = 51.6 ppm (s).

^{31}P NMR (121 MHz, 300 K, C_6D_6): δ = 51.6 ppm (s).

^{13}C{^{1}H} NMR (101 MHz, 300 K, C_6D_6): δ = 221.3 (d, $^1J(^{31}P-^1H)$ = 47.9 Hz), 49.7 (d, $^2J(^{31}P-^1H)$ = 30.3 Hz), 25.4 ppm (d, $^3J(^{31}P-^1H)$ = 3.7 Hz).

NMR data are consistent with our previous report.[4]

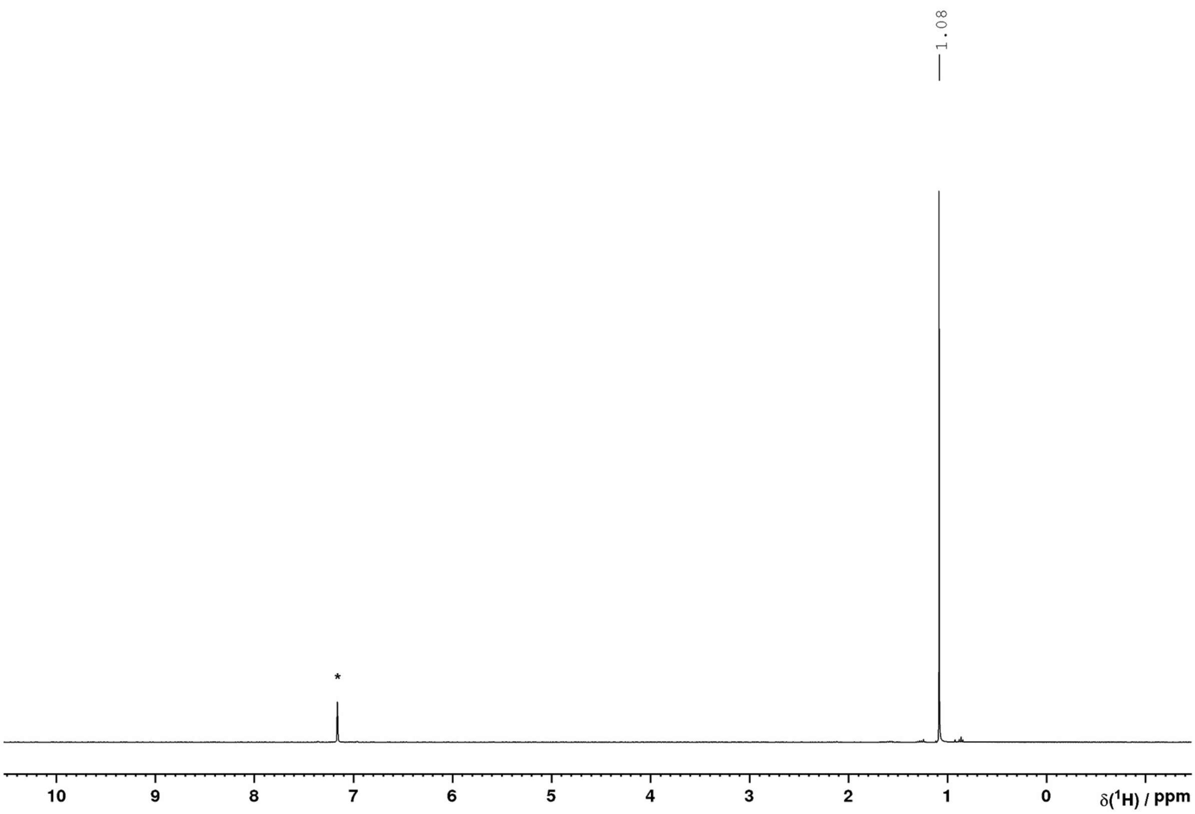

Figure S41. ^{1}H NMR spectrum of P(C(O)*t*Bu)$_3$ (**8b**) in C_6D_6 (*).

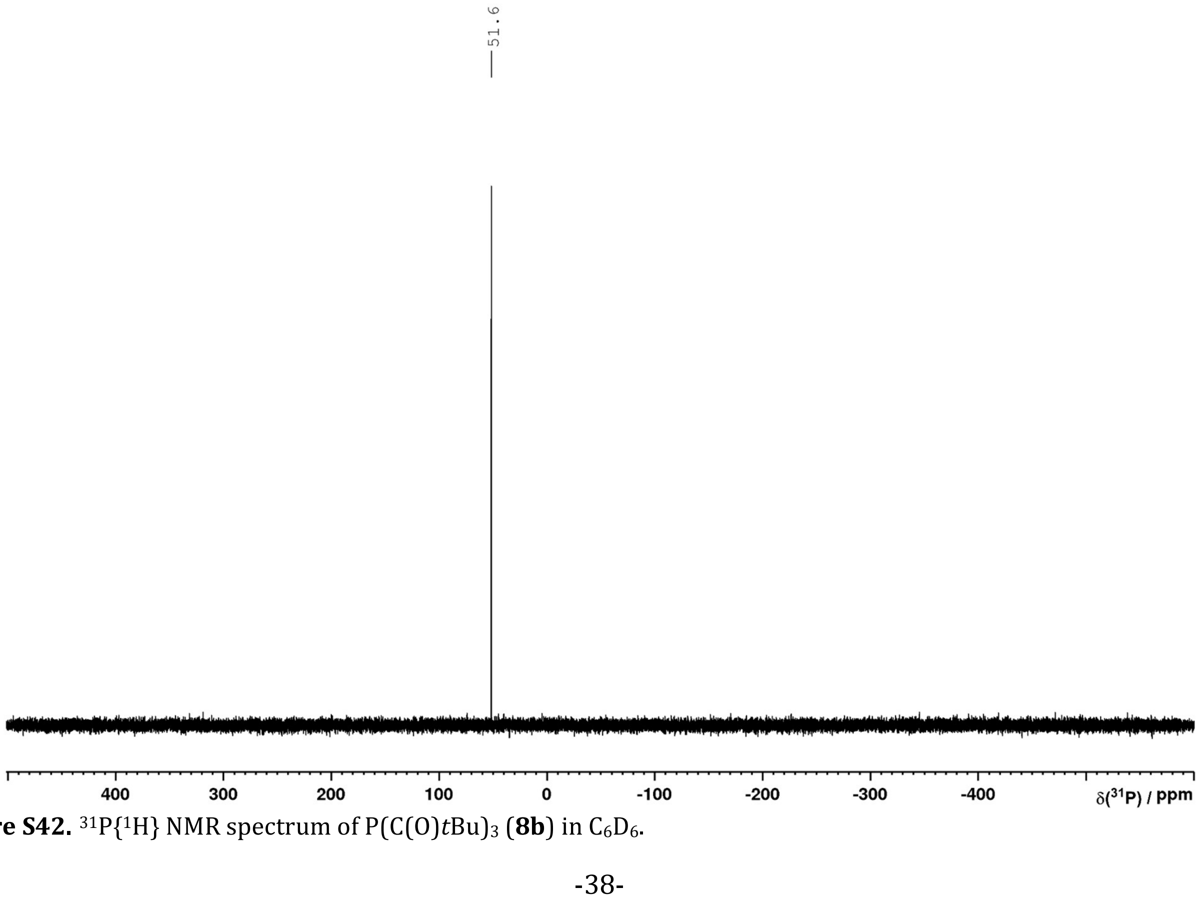

Figure S42. $^{31}P\{^{1}H\}$ NMR spectrum of P(C(O)*t*Bu)$_3$ (**8b**) in C_6D_6.

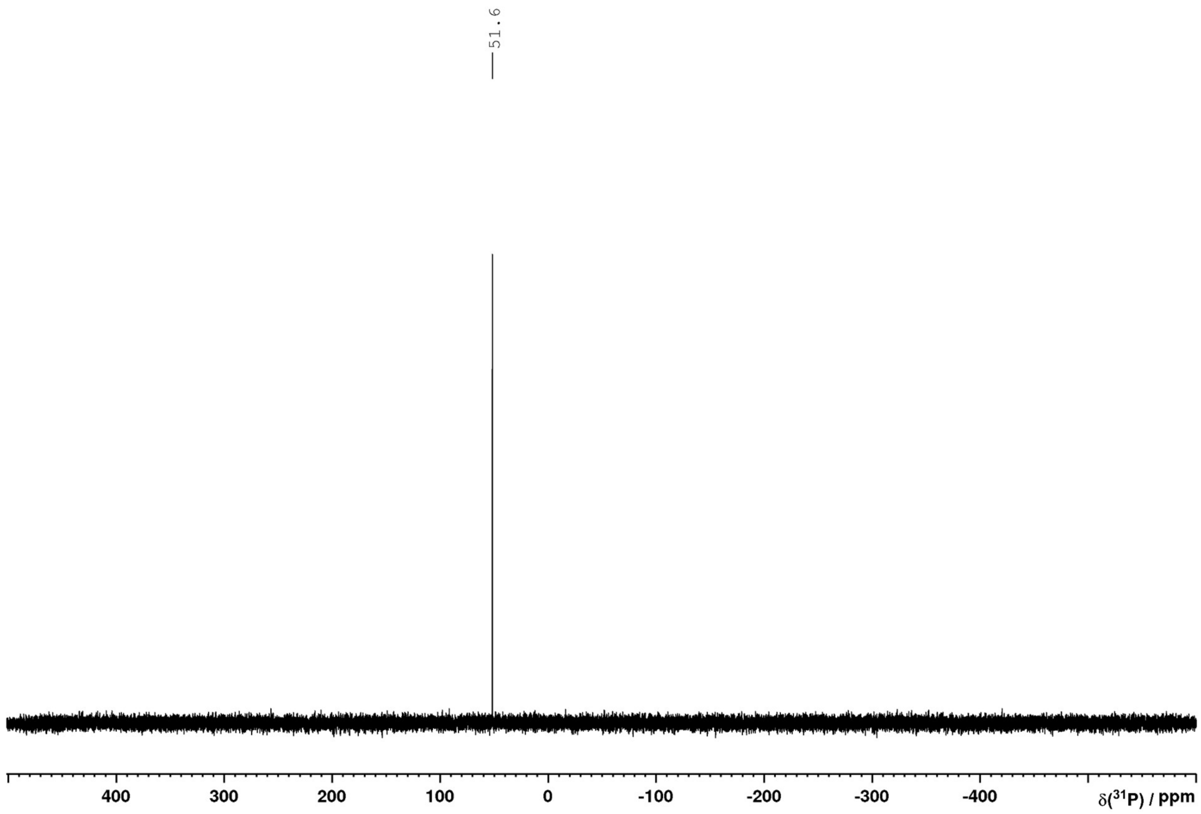

Figure S43. ^{31}P NMR spectrum of $P(C(O)tBu)_3$ (**8b**) in C_6D_6.

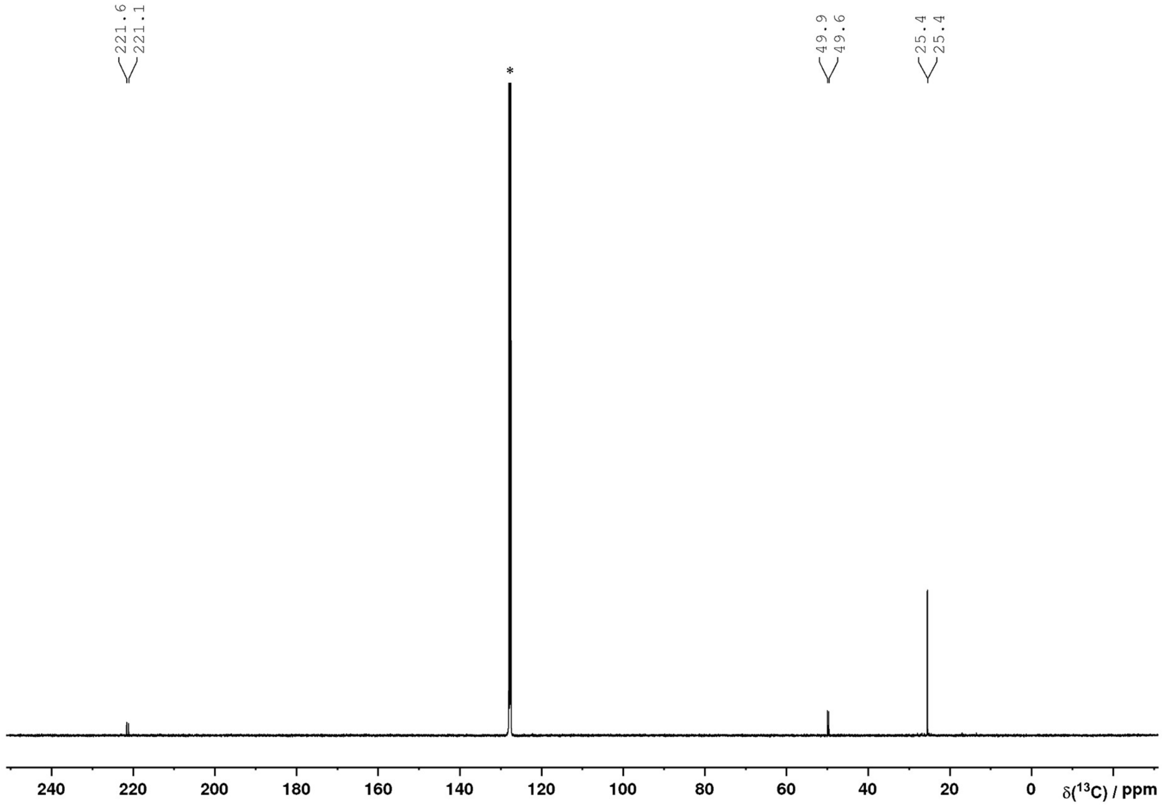

Figure S44. $^{13}C\{^1H\}$ NMR spectrum of $P(C(O)tBu)_3$ (**8b**) in C_6D_6 (*).

4. Stannylation of Red Phosphorus (P_{red}) without a glovebox

4.1. General procedures for the stannylation of P_{red} using Bu_3SnH and Bu_3SnOMe under near-UV LED irradiation without using a glovebox

The following procedures are initially prepared under open air conditions and without the use of a glovebox.

Standard Schlenk technique:

P_{red} (0.4 mmol, 12.4 mg) was weighed on the open bench and added to a 10 mL, flat-bottomed Schlenk tube. The atmosphere of the system was exchanged to N_2(g), with the help of a Schlenk line, by applying vacuum and then back-filling with N_2(g). This cycle was repeated three times. Under a flow of N_2(g), Bu_3SnOMe (17.3 µL, 0.06 mmol), undried 'bench' PhMe (50 µL) and Bu_3SnH (16.1 µL, 0.06 mmol) were added. The Schlenk tube was sealed, placed in a water-cooled block to maintain near-ambient temperature, and irradiated with UV light (365 nm, 4.3 V, 700 mA, Osram OSLON SSL 80) for 4 days. The reaction was opened to air, and Ph_3PO (12.6 mg, 0.0453 mmol) and 'bench' PhMe (500 µL) were added. The resulting mixture analysed by ^{1}H, $^{31}P\{^{1}H\}$, and ^{31}P NMR spectroscopy.

Freeze-pump-thaw:

To a 10 mL, flat-bottomed, stoppered Schlenk tube on the open bench were added P_{red} (0.4 mmol, 12.4 mg), undried 'bench' PhMe (50 µL), Bu_3SnH (16.1 µL, 0.06 mmol) and Bu_3SnOMe (17.3 µL, 0.06 mmol). The mixture was freeze-pump-thaw degassed three times using a Schlenk line in order to exchange the atmosphere of the system to N_2(g). The tube was sealed and placed in a water-cooled block to maintain near-ambient temperature, and irradiated with UV light (365 nm, 4.3 V, 700 mA, Osram OSLON SSL 80) for 4 days. The reaction was opened to air, and Ph_3PO (9.5 mg, 0.034 mmol) and 'bench' PhMe (500 µL) were added. The resulting mixture analysed by ^{1}H, $^{31}P\{^{1}H\}$, and ^{31}P NMR spectroscopy.

Open air:

To a 10 mL, flat-bottomed, stoppered tube on the open bench were added P_{red} (0.4 mmol, 12.4 mg), PhMe (50 µL), Bu_3SnH (16.1 µL, 0.06 mmol) and Bu_3SnOMe (17.3 µL, 0.06 mmol). The tube was sealed and placed in a water-cooled block to maintain near-ambient temperature, and irradiated with UV light (365 nm, 4.3 V, 700 mA, Osram OSLON SSL 80) for 4 days. The reaction was opened to air, and Ph_3PO (9.7 mg, 0.0349 mmol) and 'bench' PhMe (500 µL) were added. The resulting mixture analysed by ^{1}H, $^{31}P\{^{1}H\}$, and ^{31}P NMR spectroscopy.

Table S5. Stannylation of P_{red} using Bu_3SnH and Bu_3SnOMe under near UV LED irradiation, **without using a glove box**[a]

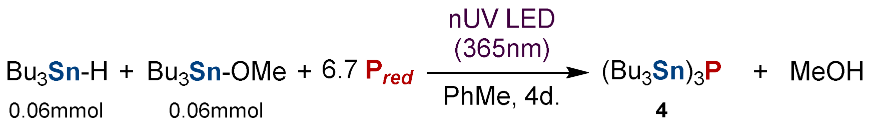

Entry	Procedure	Full conv. of Bu_3SnH?[b]	Conv. to **4** (%)[c]
1	Glovebox (dry PhMe)	✓	96
2	Glovebox ('bench' PhMe)	✓	95
3	Standard Schlenk technique	✓	92
4	Freeze-pump-thaw	✓	91
5	Open air (dry PhMe)	✓	33
6	Open air ('bench' PhMe)	✓	<5

[a] The general procedures for the stannylation of P_{red} described in this section or in section 2.1 ("glovebox") were used. [b] The full consumption of Bu_3SnH was assessed by ^1H NMR spectroscopy and the disappearance of the Sn*H* resonance that would otherwise be observed at *ca.* 5 ppm. [c] Conversions were calculated by integration of the ^{31}P resonance of **4** relative to an internal standard, which was then normalized relative to Table S1, entry 12 (defined as 99%) as described in section 1.1.

4.2. Synthesis and isolation of $(Bu_3Sn)_3P$ (4) without using a glovebox

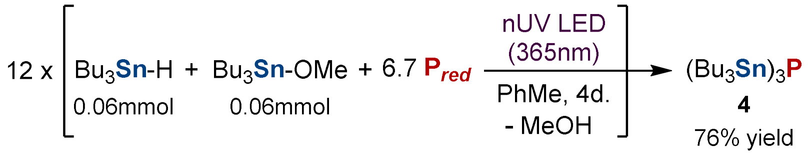

A total of twelve reactions were performed in parallel using the following procedure: P_{red} (0.4 mmol, 12.4 mg) was weighed and added on the open bench to a 10 mL, flat-bottomed, Schlenk tube. The atmosphere of the system was exchanged to N_2(g) using standard Schlenk line techniques (three cycles of vacuum and N_2(g)). Under a flow of N_2(g), Bu_3SnOMe (17.3 µL, 0.06 mmol), undried 'bench' PhMe (50 µL) and Bu_3SnH (16.1 µL, 0.06 mmol) were added. The Schlenk tube was sealed, placed in a water-cooled block to maintain near-ambient temperature, and irradiated with UV light (365 nm, 4.3 V, 700 mA, Osram OSLON SSL 80) for 4 days. The twelve reactions were opened to air and combined in a 100 mL Schlenk using 'bench' PhMe (3 x 0.5mL) to transfer and wash each tube. The combined red suspension was filtered on the open bench and the remaining solid extracted with additional 'bench' PhMe (2 x 10 mL), followed by removal of volatiles under vacuum. After distillation of the resulting yellowish oil under vacuum (*ca.* 105 °C, 10^{-5} mbar), the product (**4**) was obtained as a colourless oil (329.1 mg, 76%).

NMR data are identical to those given in section 2.2.

5. (Hydro)Stannylation of Red Phosphorus (P_{red}) promoted by chemical radical initiators

5.1. General procedure and optimisation for the hydrostannylation of P_{red} using Bu_3SnH and AIBN (0.06 mmol scale)

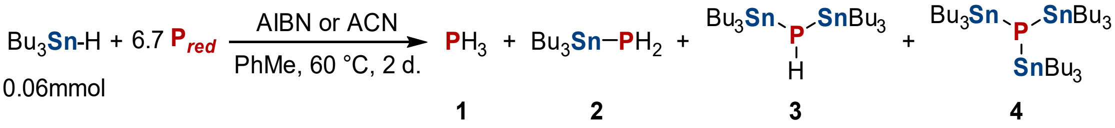

To a 10 mL, flat-bottomed, stoppered tube were added P_{red} (0.4 mmol, 12.4 mg), PhMe (50 µL), AIBN (azo*bis*(isobutyronitrile), 0.006 mmol, as a stock solution in PhH) and Bu_3SnH (16.1 µL, 0.06 mmol). The tube was sealed, wrapped in Al foil to exclude light, and heated to 60 °C for 2 days. Ph_3PO (0.02 mmol, stock solution in benzene) was subsequently added to act as an internal standard. The resulting mixture was analysed by ^{1}H, $^{31}P\{^{1}H\}$, and ^{31}P NMR spectroscopy, as shown in Figures S45 and S46, below.

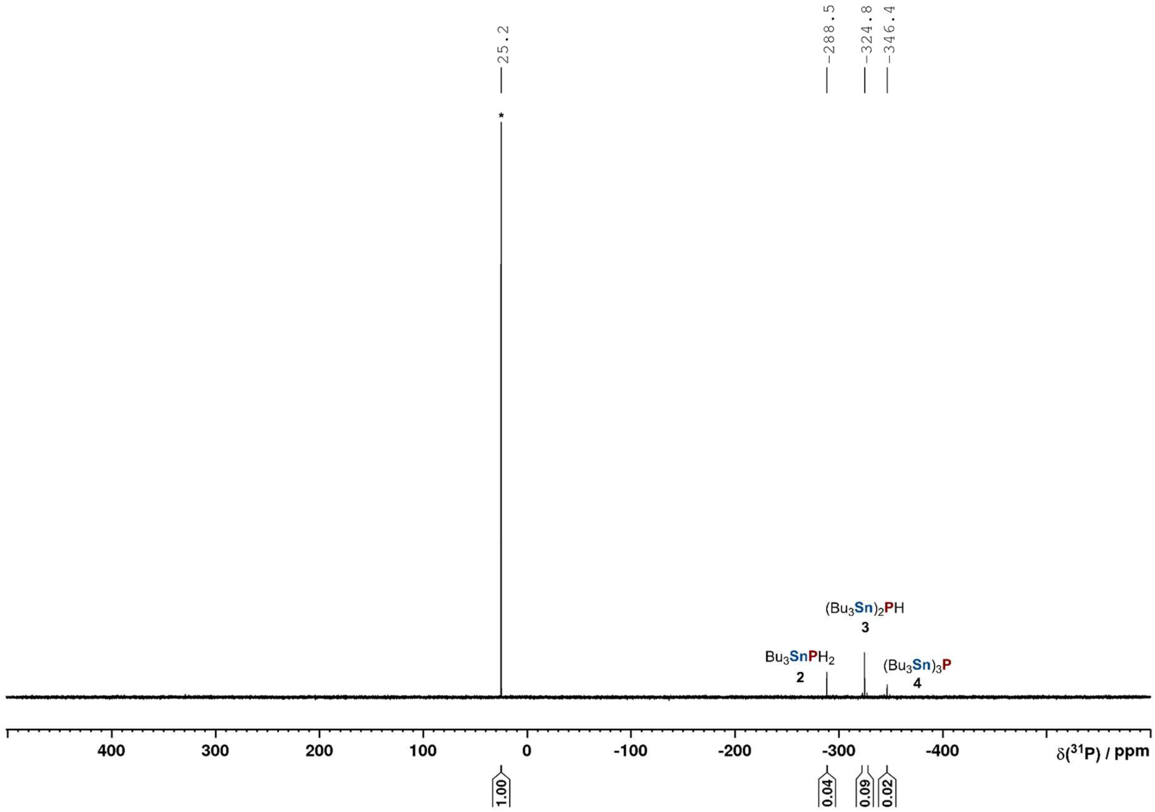

Figure S45. $^{31}P\{^{1}H\}$ NMR spectrum for the reaction of P_{red} with Bu_3SnH (0.06 mmol) and AIBN (0.006 mmol) in PhMe, heated to 60 °C for 2 days. * marks the internal standard Ph_3PO (0.04 mmol).

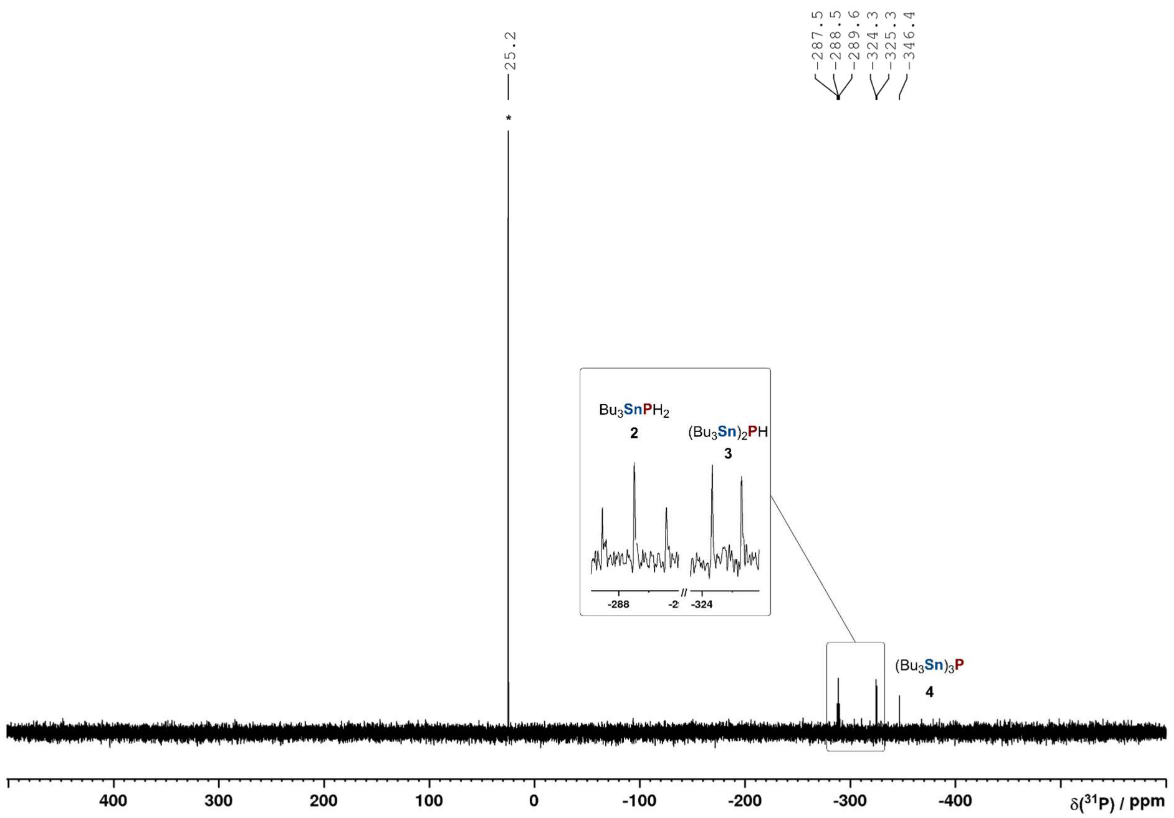

Figure S46. ^{31}P NMR spectrum for the reaction of P_{red} with Bu_3SnH (0.06 mmol) and AIBN (0.006 mmol) in PhMe, heated to 60 °C for 2 days. The insets show expansions of the signals attributed to Bu_3SnPH_2 (**2**) and $(Bu_3Sn)_2PH$ (**3**), highlighting their multiplicity due to $^1J(^{31}P-^1H)$ couplings. * marks the internal standard Ph_3PO (0.04 mmol).

Table S6. Optimization of hydrostannylation of P_{red} using Bu_3SnH and AIBN or ACN[a]

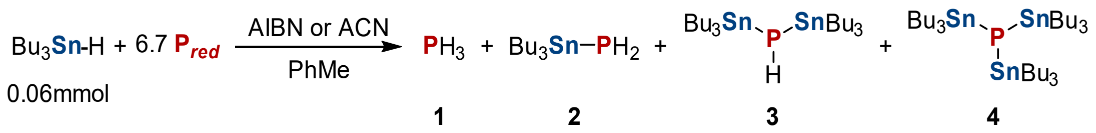

Entry	Radical initiator (mmol)	Temperature (°C)	Time (days)	Full conv. of Bu_3SnH?[b]	Relative total conv. to **1-4** (%)[c]
1	AIBN (0.006)	60	1	X	12
2	AIBN (0.006)	60	2	X	15
3	AIBN (0.012)	80	3	X	10
4	AIBN (0.012)	60	4	X	9[d]
5	AIBN (0.06)	80	4	X	5[d,e]
6	ACN (0.006)[f]	60	1	X	traces
7	ACN (0.006)[f]	60	2	X	traces
8	ACN (0.012)[f]	60	4	X	8
9	ACN (0.06)[f]	80	4	X	5[d]

[a] The general procedure described in this section was modified to use the indicated amount radical initiator, temperature and time. [b] The full consumption of Bu_3SnH was assessed by ^1H NMR spectroscopy and the disappearance of the Sn*H* resonance that would otherwise be observed at *ca.* 5 ppm. [c] Conversions were calculated by integration of the ^{31}P resonances of **1**-**4** relative to an internal standard, which was then normalized relative to Table S1, entry 12 (defined as 99%) as described in section 1.1.[d] Only product $(Bu_3Sn)_3P$ (**4**) was observed. [e] traces of P_4 were observed. [f] ACN (1,1'-azo*bis*(cyclohexanecarbonitrile)) was used instead of AIBN.

5.2. General procedure and optimisation for the stannylation of P_{red} using Bu_3SnH, Bu_3SnOMe and AIBN (0.06 mmol scale)

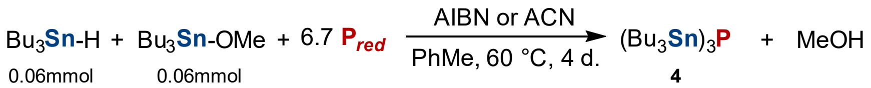

To a 10 mL, flat-bottomed, stoppered tube were added P_{red} (0.4 mmol, 12.4 mg), PhMe (50 µL), AIBN (azo*bis*(isobutyronitrile), 0.012 mmol, as a stock solution in PhH), Bu_3SnH (16.1 µL, 0.06 mmol) and Bu_3SnOMe (17.3 µL, 0.06 mmol). The tube was sealed, wrapped in Al foil to exclude light, and heated to 60 °C for 4 days. Ph_3PO (0.02 mmol, stock solution in benzene) was subsequently added to act as an internal standard. The resulting mixture was analysed by ^1H, $^{31}P\{^1H\}$, and ^{31}P NMR spectroscopy, as shown in Figures S47 and S48, below.

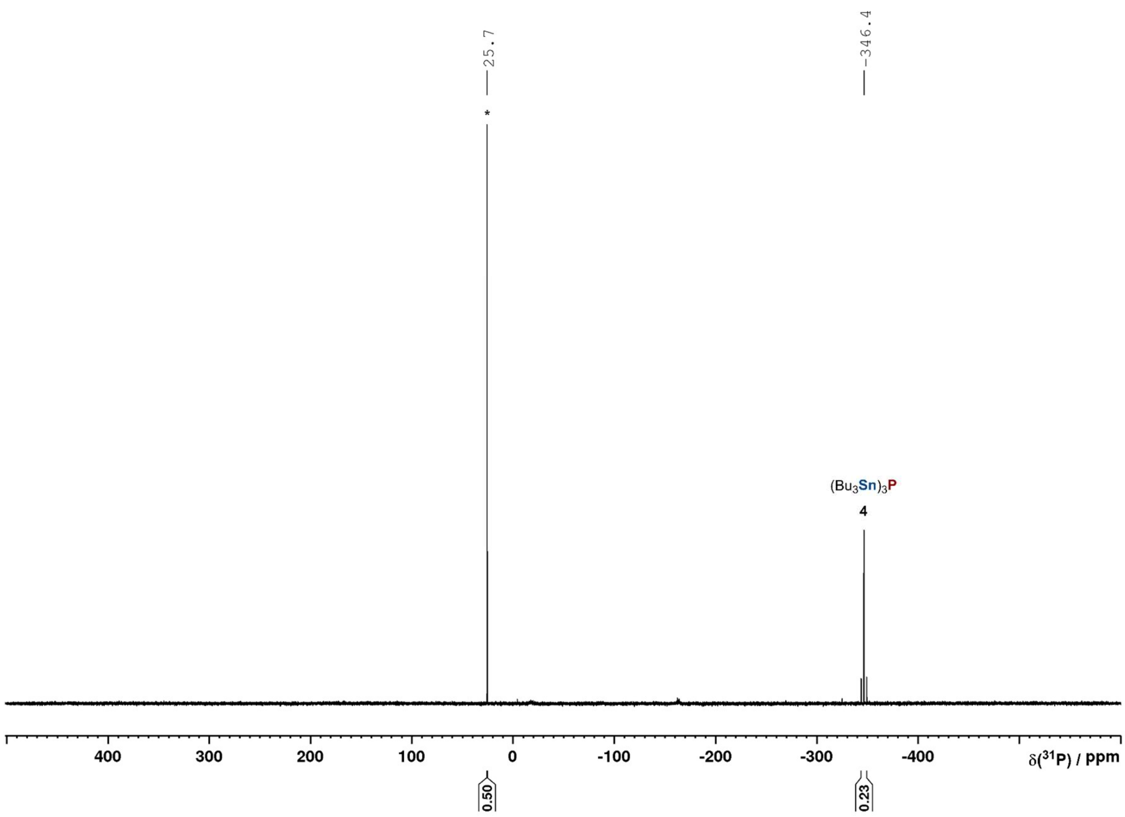

Figure S47. $^{31}P\{^1H\}$ NMR spectrum for the reaction of P_{red} with Bu_3SnH (0.06 mmol), Bu_3SnOMe (0.06 mmol) and AIBN (0.012 mmol) in PhMe, heated to 60 °C for 4 days. * marks the internal standard Ph_3PO (0.04 mmol).

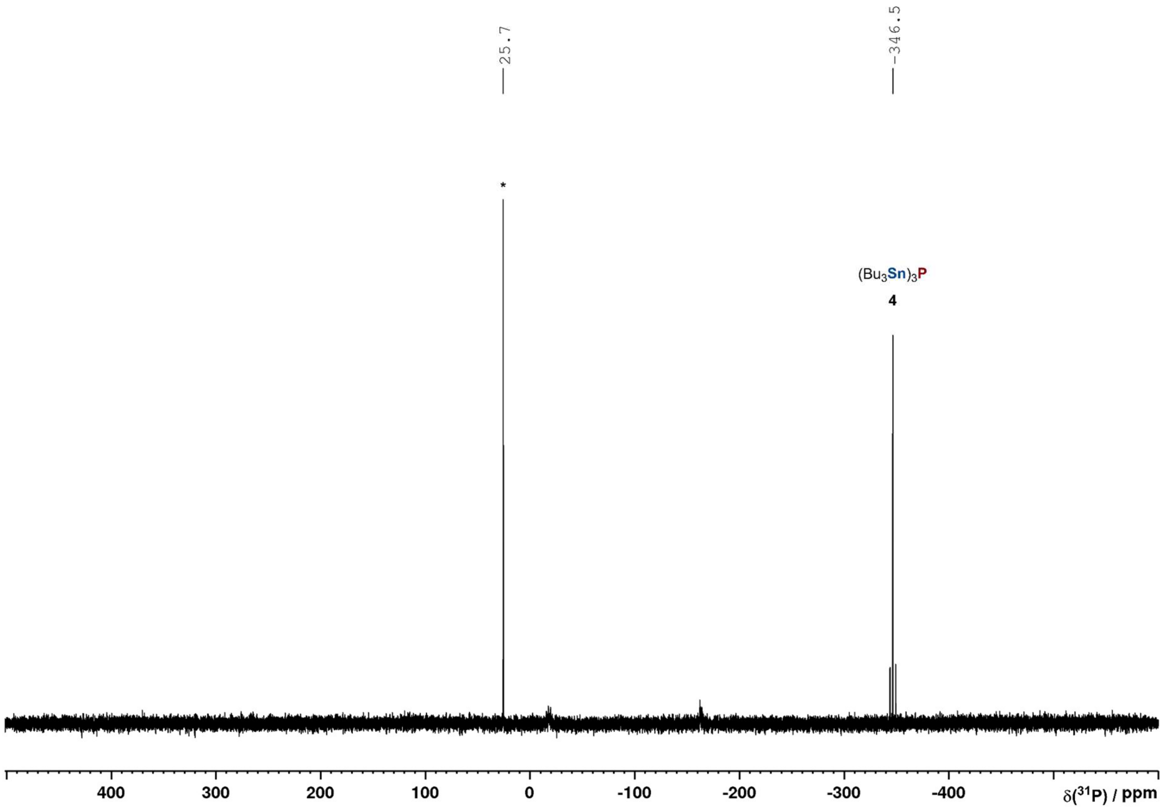

Figure S48. $^{31}P\{^{1}H\}$ NMR spectrum for the reaction of P_{red} with Bu_3SnH (0.06 mmol), Bu_3SnOMe (0.06 mmol) and AIBN (0.012 mmol) in PhMe, heated to 60 °C for 4 days. * marks the internal standard Ph_3PO (0.04 mmol).

Table S7. Optimization of stannylation of P_{red} using Bu_3SnH, Bu_3SnOMe and AIBN or ACN[a]

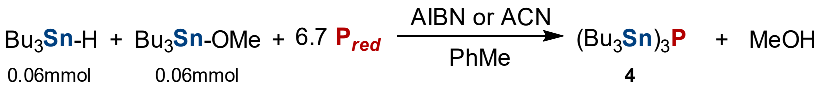

Entry	Radical initiator (mmol)	Temperature (°C)	Time (days)	Full conv. of Bu_3SnH?[b]	Conv. to **4** (%)[c]
1	AIBN (0.006)	60	2	X	20[d,e]
2	AIBN (0.006)	60	3	X	15[d]
3	AIBN (0.012)	40	2	X	8[d]
4	AIBN (0.012)	60	2	X	12[d]
5	AIBN (0.012)	60	3	X	17
6	AIBN (0.012)	60	4	X	23[d,e]
7	AIBN (0.012)	80	2	X	4
8	AIBN (0.12)	60	2	✓	traces
9	AIBN (012)	80	2	✓	traces
10	ACN (0.012)[f]	60	2	X	2[d]
11	ACN (0.12)[f]	60	2	✓	10

[a] The general procedure described in this section was modified to use the indicated amount radical initiator, temperature and time. [b] The full consumption of Bu_3SnH was assessed by ^{1}H NMR spectroscopy and the disappearance of the Sn*H* resonance that would otherwise be observed at *ca*. 5 ppm. [c] Conversions were calculated by integration of the ^{31}P resonance of **4** relative to an internal standard, which was then normalized relative to Table S1, entry 12 (defined as 99%) as described in section 1.1. [d] Product $(Bu_3Sn)_2PH$ (**3**) was observed in <5%. [e] Traces of signals attributed to $P_7(SnBu_3)_3$ were observed. [f] ACN (1,1'-azo*bis*(cyclohexanecarbonitrile)) was used instead of AIBN.

References

[1] U. Lennert, P. B. Arockiam, V. Streitferdt, D. J. Scott, C. Rödl, R. M. Gschwind, R. Wolf, *Nat. Catal.* **2019**, *2*, 1011–1106.

[2] M. Till, V. Streitferdt, D. J. Scott, M. Mende, R. M. Gschwind, R. Wolf, *Chem. Commun.* **2022**, *58*, 1100–1103.

[3] G. Fritz, K. D. Hoppe, W. Hönle, D. Weber, C. Mujica, V. Manriquez, H. G. v. Schnering, *J. Organomet. Chem.* **1983**, *249*, 63–80.

[4] D. J. Scott, J. Cammarata, M. Schimpf, R. Wolf, *Nat. Chem.* **2021**, *13*, 458–464.
